# Difference on cone size preferences between two coniferous species by Great Spotted Woodpecker (*Dendrocopos major*)

**DOI:** 10.7717/peerj.3288

**Published:** 2017-05-31

**Authors:** Łukasz Dylewski, Reuven Yosef, Łukasz Myczko

**Affiliations:** 1 Institute of Zoology, Poznań University of Life Sciences, Poznań, Poland; 2 Eilat Campus, Ben-Gurion University of the Negev, Eilat, Israel

**Keywords:** Pre-dispersal seed predation, Phenotypic selection, Norway spruce, Great Spotted Woodpecker, Anvils, Scots pine

## Abstract

The number of species that specialize in pre-dispersal seed predation is relatively small. Examples of specialized pre-dispersal seed predators adapted to feeding on closed cones include vertebrate species like Crossbills, Squirrels, Nutcrackers and Woodpeckers. Seed predation selects against certain phenotypic features of cones and favors another phenotypic features. In this study, we document preferences of the Great Spotted Woodpecker (*Dendrocopos major*) for specific traits in the cones of Norway spruce (*Picea abies*) and Scots pine (*Pinus sylvestris*). We found that the Great Spotted Woodpecker prefers to feed on medium sized Norway spruce cones. The results suggest a disruptive selection that favors the extreme cone lengths in Norway spruce. In Scots pine, the woodpeckers avoided cones with large apophyses. Further, the selectivity for the specific characteristics of the cones is probably related to the configuration of the anvil, a place at which woodpeckers extract seeds from the cones. We think that the Great Spotted Woodpecker preferences in relation to the morphological characteristics of cones are a key to the design of the anvil in order to maximize the use of it as a tool for processing cones of both the Norway spruce and the Scots pine.

## Introduction

Natural selection is an essential process in the ecology and evolution of ecosystems which arises from both biotic and biotic-interaction (Brockhurst et al., 2014). The adaptation in one of the interacting organisms creates an increasing selection pressure on the opposite species which results in co-evolution. The interspecific interactions can lead to the establishment of specific characteristics. The interactions between seed-eating animals and plants this may result in a mutualistic and/or antagonistic relationship. Coniferous species have evolved many morphological and chemical defenses to protect their propagules (Lewinsohn et al., 1991; Coffey, Benkman & Milligan, 1999; Phillips & Croteau, 1999). In coniferous cones, the creation of defense mechanisms in the form of increased wood tissue is the result of selection pressures exerted by specialized conifer-seed-eating animals (Leslie, 2011). However, this investment is not without cost. There is a tradeoff between resources allocated to defense and the seeds. A greater investment in seed defense in the form of thicker cone scales, seed coat or resin negatively influences the seeds, the number of seeds produced and the amount of energy reserves deposited in each of the seeds (Benkman, 1995). The appearance of the first birds and arboreal mammals in the late Jurassic, and their subsequent radiation in the early Cenozoic, probably favored an increase in seed defenses (Leslie, 2011). Fossil evidence suggests a radiation of seed-eating organisms (insects, birds, and mammals) that resulted in the cones of that period developing thicker cone scales and spiny apophyses to protect against seed predation (Molinari et al., 2006; Leslie, 2011). Currently, only a few groups of animals are able to forage on conifer seeds before their dispersal. These groups include Crossbills (*Loxia* spp.), Squirrels (*Sciuridae* spp.), Nutcrackers (*Nucifraga* spp.), Woodpeckers (*Picidae* spp.) and some insects (e.g., *Dioryctria abietella, Pissodes validrostris*).

Several researchers have demonstrated that selective pressure exerted by pre-dispersal seed predators on conifer species results in the confers increasing their energy investment in seed protection (e.g., Benkman, Holimon & Smith, 2001; Siepielski & Benkman, 2008; Benkman, Fetz & Talluto, 2012). The presence of pre-dispersal seed predators usually results in the coniferous species producing cones with larger and thicker scales (Benkman et al., 2003; Mezquida & Benkman, 2005). This is especially evident in isolated populations where only one dominant pre-dispersal seed predator may occur, and that can induce a particularly strong selection pressure on cone morphology (Benkman, Holimon & Smith, 2001). Also, in the isolated areas where conifer-seed-eating animals selected different features of cone, it may eventually lead to the uniformity of some features (Benkman & Parchman, 2013). An example is that of an isolated population of Crossbills (*Loxia megaplaga*) that occur on the Hispaniola islands that have developed a large and massive beak in response to the cones of the co-evolved Hispaniolan pine (*Pinus occidentalis*) that have thicker scales and spiky apophyses, especially when compared to the Cuban pine (*Pinus cubensis*) on the island of Cuba which lacks Crossbills (Parchman, Benkman & Mezquida, 2007).

Generally, pre-dispersal seed predators should prefer areas, where the investment of the coniferous species in protective mechanisms is relatively low. In the case of the presence of a sedentary specialized predator, over generations, this could lead to an increase in the predator-specific defense of the cone (Benkman & Siepielski, 2004).

The Great Spotted Woodpecker (*Dendrocopos major*) is a species that specializes in feeding on seed from closed conifer cones, especially in the winter (Hogstad, 1971; Osiejuk, 1998; Kędra & Mazgajski, 2001). However, the foraging success of an individual is dependent greatly on its ability to prepare an appropriate anvil for processing the cones and extracting the seeds. An anvil is usually a natural crack in the trunk of a tree or branches that are then modified by the woodpecker. Previous research on the phenotypic selection exerted by the Great Spotted Woodpecker showed a significant pressure on this species to specific cone-traits in lowland forests where they forage only on Scots pine seeds (Myczko & Benkman, 2011).

The aim of our study is to elucidate as to whether two morphologically different characteristics of Norway spruce (*Picea abies*) and Scots pine (*Pinus sylvestris*) cones is related to predation probability by the Great Spotted Woodpecker.

## Materials and Methods

### Study area

Our study was conducted in mixed mountain forests in West Sudetes (50°55′N 15°46′E), southwest Poland. The study area consists of managed forests dominated by mature Norway spruce (60%) with co-occurring Scots pine (25%) and Larch (*Larix decidua*, 5%), and a mixture of deciduous species, mainly European beech (*Fagus sylvatica*), Pedunculate oak (*Quercus robur*) and Silver birch (*Betula pendula*).

### Data collection

In the winter of 2011/12, we attempted to locate as many woodpecker anvils as possible in the forest. Field work was performed during winter with good cone crops of Norway spruce and Scots pine. We selected the 20 most regularly used anvils, which were at a distance of at least 200 m from each other, and assumed were used by different individuals. We collected foraged cones during March–April 2012, and in September 2012 collected unforaged Norway spruce and Scots pine cones from a 15 m radius of each of the anvils, as compared to a 10 m radius in the lowland studies (Kędra & Mazgajski, 2001; Myczko & Benkman, 2011), because the forest stand structure and number of individuals of coniferous trees in the mountains differed from the lowlands, where there are mainly managed Scots pine monocultures. We randomly collected 100–300 foraged cones from each of the 20 anvils, and 100–300 unforaged cones of both coniferous species from within the woodpecker’s known foraging area. Cones were transported to the laboratory and submerged in water for 24 h. We then measured cone characteristics (cf. Benkman et al., 2003; Myczko & Benkman, 2011). In both spruce and pine cones we measured the length of each cone, but only in pine cones did we also note two apophyses categories on the scales (small or large). In order to check the probability of the occurrence of cones with large apophyses next year (August 2013) we collected 80 additional unforaged Scots pine cones from a 15 m radius each of the anvil. The cones were also treated as previous described.

### Data analysis

We used a general linear mixed model (GLMM) with a logit link function with binomial errors for all the analyses. In the case of Norway spruce, we tested the probability of predation compared to cone length. In Scots pine, we tested the probability of predation compared to cone length, apophyses characteristics, and interaction between cone length and apophyses characteristics. For cones collected in 2013, we tested the relation of apophyses characteristics with cone length. We assigned 0 for small apophyses and 1 for larger apophyses. In all GLMM analysis, the anvils were treated as a random variable. We use a cubic spline to visualize the probability of woodpecker predation to the variables studied. All GLMM’s and cubic splines were performed in R Core Team (2016) using R-package nlme (Pinheiro et al., 2016), lme4 (Bates & Maechler, 2010) and mgcv (Wood, 2015).

## Results

**Figure 1:**
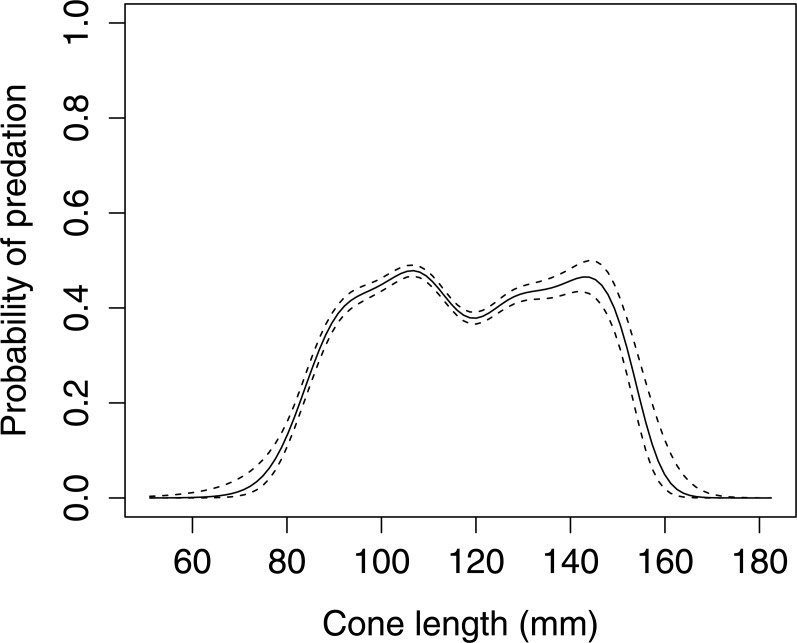
The probability of seed predation by Great-Spotted Woodpeckers (*Dendrocopos major*) in relation to Norway spruce cone length (*n*= 8,081 cones). The solid curves are based on cubic splines and the dashed lines represent +1SE.

Cone length significantly influenced woodpecker predation probability in Norway spruce (GLMM *F*_1,8080_ = 5.336 *P* = 0.021, Fig. 1). The graphical visualization (cubic spline) showed that the probability of foraging on the Norway spruce cones is highest between 90–150 mm length. Smaller or larger cones were generally avoided by the woodpeckers.

**Figure 2:**
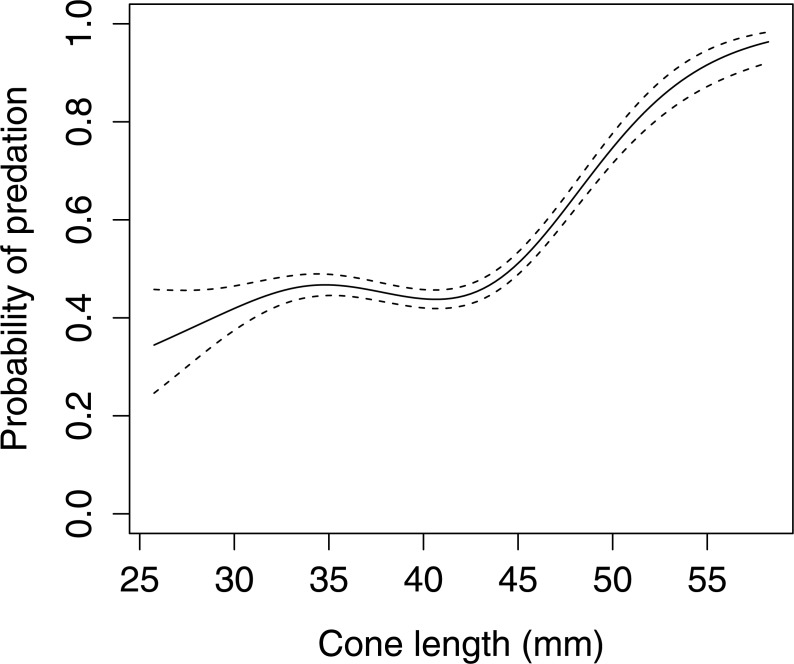
The probability of seed predation by Great-Spotted Woodpeckers (*Dendrocopos major*) on Scots pine cone with or without apophysis (*n*= 1,812). The solid curves are based on cubic splines and the dashed lines represent +1SE.

**Figure 3:**
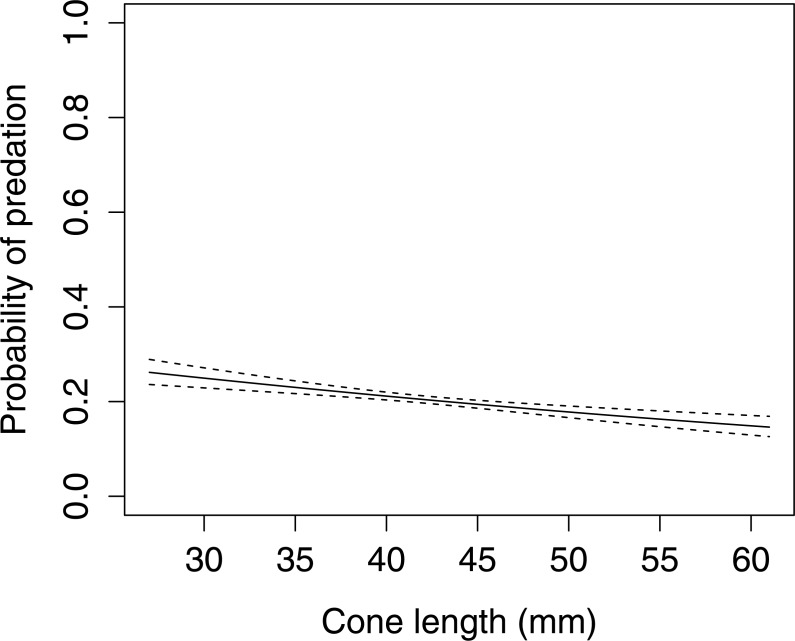
The probability of seed predation by Great-Spotted Woodpeckers (*Dendrocopos major*) on Scots pine cones with large apophysis (*n*= 3,584). The solid curves are based on cubic splines and the dashed lines represent +1SE.

**Figure 4:**
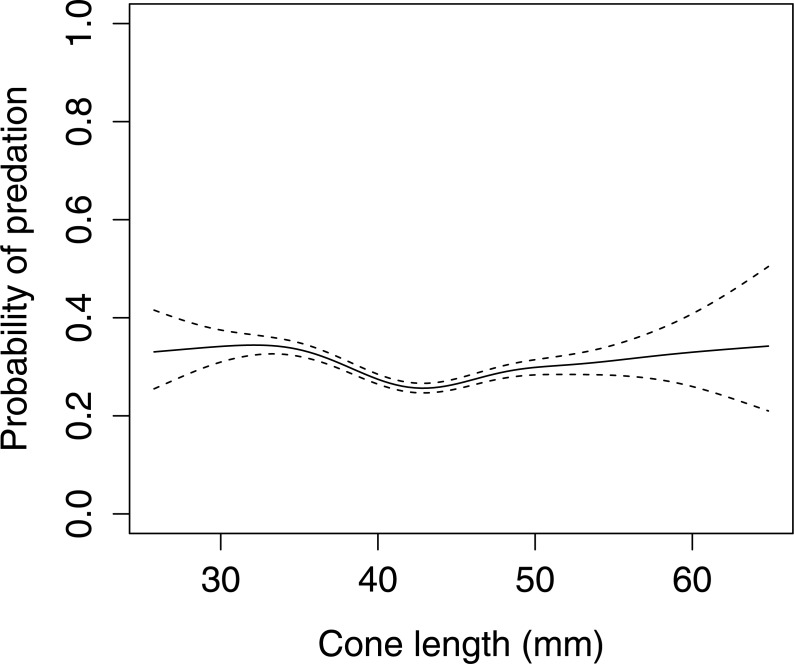
The overall probability of seed predation by Great-Spotted Woodpeckers (*Dendrocopos major*) on Scots pine cones (*n*= 5,396 cones). The solid curves are based on cubic splines and the dashed lines represent +1SE.

In Scots pine cones, the length of the cones (GLMM *F*_1,5381_ = 13.042 *P* < 0.001), category of apophyses (GLMM *F*_1,5381_ = 594.347 *P* < 0.001), and interaction between the length of cones and apophyses (GLMM *F*_1,5381_ = 16.756 *P* < 0.001) significantly affected the probability of predation by Great Spotted Woodpecker. When cones had small apophyses, the probability of predation increased with increasing cone length (Fig. 2), whereas when cones had large apophyses the probability of predation decreased with increased cone length (Fig. 3). Without dividing cones based on apophyses categories the probability of predation by Great Spotted Woodpecker is the highest at approximately 34 mm cone length (Fig. 4).

**Figure 5:**
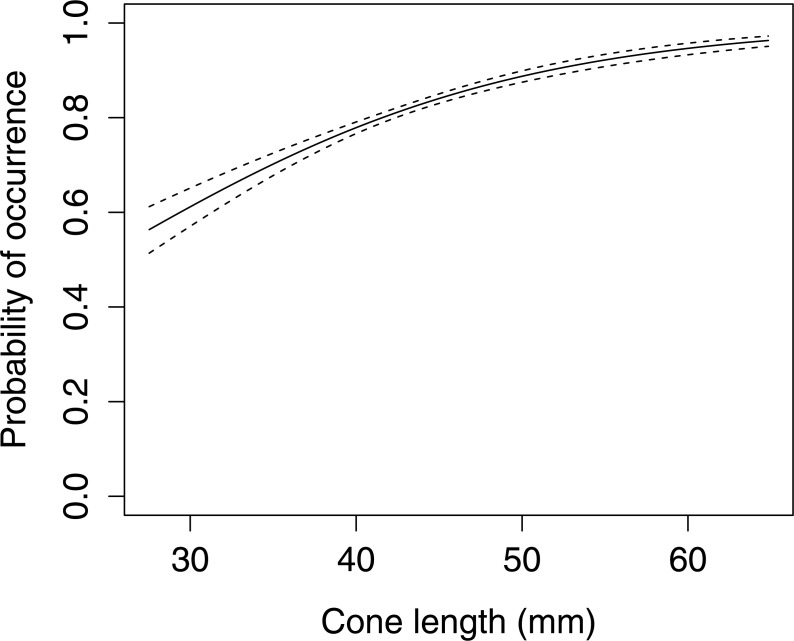
The probability of occurrence of large apophyses on Scots pine cone scales (*n*= 1,600). The solid curves are based on cubic splines and the dashed lines represent +1SE.

The occurrence of large apophyses increased with increasing cone length (Fig. 5, GLMM *F*_1,1598_ = 39.439 *P* < 0.001). A comparison of cone characteristics showed that there were no significant differences between anvils.

## Discussion

Our data suggests that the Great Spotted Woodpecker prefers foraging on medium size Norway spruce cones, i.e., those between 90 and 150 mm. It appears that the selection pressure is constant in spite of a very wide range of cone lengths (Fig. 1). This phenotypic selection exerted by the woodpeckers on the size of the cones of Norway spruce suggests that the trees that produce either extremely short or long cones will be favored by selection exerted by the woodpeckers. Large cones may be more difficult to transport and to position in the anvils. Our findings corroborate previous studies that demonstrated that cone length was a key factor that determined the probability of pre-dispersal seed predation by the granivorous animals (Molinari et al., 2006; Myczko & Benkman, 2011). Red squirrel (*Sciurus vulgaris*), conform to the principles of optimal foraging (Charnov, 1976) and prefer to feed on long spruce cones that contain a greater number of seeds, because they are not limited by the use of tools such as the anvil, and feed wherever convenient whether on the ground or in the trees. Molinari et al. (2006) suggested that the Red Squirrel probably evaluate cone quality based on cone length. The Great Spotted Woodpecker also avoid small cones because they have proportionately more underdeveloped seeds (Ehrenberg et al., 1955). Madsen (1972) found that spruce cones processed by Great Spotted Woodpecker contained a considerable amount of seeds which can be the result of seed dispersion.

The probability of predation by Great Spotted Woodpecker on Scots pine cones was stable when only length was taken into consideration. Nevertheless, it changed dramatically when we considered the apophyses forms. In cases when cones were without, or with small apophyses, predation increased considerably. However, Great Spotted Woodpecker, in general, avoided small cones and the probability of predation increased in cones ≥45 mm length. Significant differences in probability of predation depending on the length of cones was observed for cone without and with small apophyses. Cones with large apophyses were avoided by the woodpeckers and probability of predation decreased with increasing cone length above a certain threshold. These findings are similar to Myczko & Benkman (2011) who showed that Great Spotted Woodpecker avoids cones with large apophyses and preferred medium-sized cones with smaller apophyses.

We did not find a clear preference for Scots pine cone length and is different from the lowland forest where the most preferred cone length was 43 mm (Kędra & Mazgajski, 2001), and 44 mm for most common cone apophyses type, with a strong preference for small cones without apophyses (Myczko & Benkman, 2011).

However, on the differences in the choice of pine cones by the Great Spotted Woodpecker in this study, compared to previous studies from the lowlands, showed that it will most likely affect the co-occurrence of seeds in their diet from cones of Norway spruce and more common Scots pine cones with large apophyses than in the lowlands (Skrzyszewski, 2001). This difference can be probably explained by the adaptation of the use of an anvil to process the better protected Norway spruce cones.

Both Norway spruce and Scots pine cones with small apophyses have smooth exteriors. It appears that this feature is preferred and not only allows a better fit of the cone in the anvil hole but also provides stability during the extraction of seeds. In the case of a cone with large apophyses, the diameter increases with the length of the cones making it difficult to handle and place in the crevice of the anvil. One must also take into account that in certain interval lengths the diameter of the pine cones with large apophyses is greater than the diameter of the spruce cone and which could also explain why cones bigger than a given diameter/length are excluded by the woodpecker. Future studies should stress the cone-handling limitations of the woodpeckers in order to elucidate if handling-costs or anvil size are the limiting factor.

Apophyses length is heritable in various cone traits (cf. Benkman, Parchman & Mezquida, 2010) and is constant for a given individual Scots pine (Sevik & Topaçoğlu, 2015). Great Spotted Woodpecker selects cones without or with small apophyses of preferred length. Previous studies have shown that selection pressure caused by the pre-dispersal seed predators favors these features (e.g., Mezquida & Benkman, 2010; Benkman & Parchman, 2009). In contrast, longer pine cones with thicker scales and many seeds appear to be preferred by the Lodgepole Pinecone Borer Moth (*Eucosma recissoriana*). Moths (mainly with family Pyralidae and Tortricidae) preferentially oviposit eggs in longer cones with more seeds because a greater amount of seed kernel mass in the cone ensures that there will be enough food to support multiple larvae (Siepielski & Benkman, 2004).

Different seed predators have different cone and tree preferences (Summers & Proctor, 1999). Previous studies indicate that animals can exert selection on different cone characteristics such as cone size, thickness of scale, presence of apophyses (Benkman et al., 2003; Siepielski & Benkman, 2007; Mezquida & Benkman, 2010; Myczko & Benkman, 2011). However, we demonstrate that the presence of two food sources, Norway spruce and Scots pine seeds, appears to modify the preferences of Scots pine cones by the Great Spotted Woodpecker.

In conclusion, we have demonstrated that cone length and apophyses size are subject to selective pressure by the Great Spotted Woodpecker. Selection pressure exerted by the Great Spotted Woodpecker favored trees that produced small and large cones in the case of Norway spruce. However, no clear cone-size preference was obvious in Scots pine. Additionally, we show that Great Spotted Woodpecker avoided Scots pine cones with large apophyses. The choice of cone size on Norway spruce is probably conducive to selecting for keeping the large variation in cone-length, owing to the preferential feeding pressures exerted on medium length cones. Hence, we assume that the Great Spotted Woodpecker optimizes the shape of its anvil for medium size Norway spruce cones, and this significantly affects the preferred size of the apophyses on the cones of the Scots pine. It is probable that the Great Spotted Woodpecker preferences in relation to the morphological characteristics of cones is a key to the design of the anvil in order to maximize the use of it as a tool for processing cones of both the Norway spruce and the Scots pine.

##  Supplemental Information

10.7717/peerj.3288/supp-1 Data S1 Raw data

## Additional Information and Declarations

### Data Availability

The following information was supplied regarding data availability: The raw data has been supplied as Data S1.

## References

[ref-1] Bates D, Maechler M (2010). http://CRAN.R-project.org/package=lme4.

[ref-2] Benkman CW (1995). The impact of tree squirrels (Tamiasciurus) on limber pine seed dispersal adaptations. Evolution.

[ref-3] Benkman CW, Fetz T, Talluto MV (2012). Variable resource availability when resource replenishment is constant: the coupling of predators and prey. Auk.

[ref-4] Benkman CW, Holimon WC, Smith JW (2001). The influence of a competitor on the geographic mosaic of coevolution between crossbills and lodgepole pine. Evolution.

[ref-5] Benkman CW, Parchman TL (2009). Coevolution between crossbills and black pine: the importance of competitors, forest area, and resource stability. Journal of Evolutionary Biology.

[ref-6] Benkman CW, Parchman TL (2013). When directional selection reduces geographic variation in traits mediating species interactions. Ecology and Evolution.

[ref-7] Benkman CW, Parchman TL, Favis A, Siepielski AM (2003). Reciprocal selection causes a coevolutionary arms race between crossbills and lodgepole pine. American Naturalist.

[ref-8] Benkman CW, Parchman TL, Mezquida ET (2010). Patterns of coevolution in the adaptive radiation of crossbills. Annals of the New York Academy of Sciences.

[ref-9] Benkman CW, Siepielski AM (2004). A keystone selective agent? Pine squirrels and the frequency of serotiny in lodgepole pine. Ecology.

[ref-10] Brockhurst MA, Chapman T, King KC, Mank JE, Paterson S, Hurst GD (2014). Running with the Red Queen: the role of biotic conflicts in evolution. Proceedings of the Royal Society B: Biological Sciences.

[ref-11] Charnov EL (1976). Optimal foraging, the marginal value theorem. Theoretical Population Biology.

[ref-12] Coffey K, Benkman CW, Milligan BG (1999). The adaptive significance of spines on pine cones. Ecology.

[ref-13] Ehrenberg C, Gustafsson Å, Forshell C, Simak M (1955). Seed quality and the principles of forest genetics. Hereditas.

[ref-14] Hogstad O (1971). Notes on the winter food of the Great Spotted Woodpecker, *Dendrocopos major*. Sterna.

[ref-15] Kędra AH, Mazgajski TD (2001). Factors affecting anvils utilization by Great Spotted Woodpecker *Dendrocopos major*. Polish Journal of Ecology.

[ref-16] Leslie AB (2011). Predation and protection in the macroevolutionary history of conifer cones. Proceedings of the Royal Society of London.

[ref-17] Lewinsohn E, Gijzen M, Savage TJ, Croteau R (1991). Defense mechanisms of conifers relationship of monoterpene cyclase activity to anatomical specialization and oleoresin monoterpene content. Plant Physiology.

[ref-18] Madsen G (1972). Stor flaggspattes fouragering pa kogel. Flora Og Fauna.

[ref-19] Mezquida ET, Benkman CW (2005). The geographic selection mosaic for squirrels, crossbills and Aleppo pine. Journal of Evolutionary Biology.

[ref-20] Mezquida ET, Benkman CW (2010). Habitat area and structure affect the impact of seed predators and the potential for coevolutionary arms races. Ecology.

[ref-21] Molinari A, Wauters LA, Airoldi G, Cerinotti F, Martinoli A, Tosi G (2006). Cone selection by Eurasian red squirrels in mixed conifer forests in the Italian Alps. Acta Oecologica.

[ref-22] Myczko Ł, Benkman CW (2011). Great Spotted Woodpeckers *Dendrocopos major* exert multiple forms of phenotypic selection on Scots pine *Pinus sylvestris*. Journal of Avian Biology.

[ref-23] Osiejuk TS (1998). Study on the intersexual differentiation of foraging niche in relation to abundance of winter food in Great Spotted Woodpecker *Dendrocopos major*. Acta Ornithologica.

[ref-24] Parchman TL, Benkman CW, Mezquida ET (2007). Coevolution between Hispaniolan crossbills and pine: does more time allow for greater phenotypic escalation at lower latitude?. Evolution.

[ref-25] Phillips MA, Croteau RB (1999). Resin-based defenses in conifers. Trends in Plant Sciences.

[ref-26] Pinheiro J, Bates D, DebRoy S, Sarkar D, R Core Team (2016). https://CRAN.R-project.org/package=nlme.

[ref-27] R Core Team (2016). https://r-project.org/foundation/.

[ref-28] Sevik H, Topaçoğlu O (2015). Variation and inheritance pattern in cone and seed characteristics of Scots pine (*Pinus sylvestris* L.) for evaluation of genetic diversity. Journal of Environmental Biology.

[ref-29] Siepielski AM, Benkman CW (2004). Interactions among moths, crossbills, squirrels and lodgepole pine in a geographic selection mosaic. Evolution.

[ref-30] Siepielski AM, Benkman CW (2007). Selection by a pre-dispersal seed predator constrains the evolution of avian seed dispersal in pines. Functional Ecology.

[ref-31] Siepielski AM, Benkman CW (2008). A seed predator drives the evolution of a seed dispersal mutualism. Proceedings of the Royal Society of London B: Biological Sciences.

[ref-32] Skrzyszewski J (2001). Variability of morphological characteristics of cones in pine stand in carpathian and sudetes. Acta Agraria et Silvestria. Series Agraria.

[ref-33] Summers RW, Proctor R (1999). Tree and cone selection by crossbills *Loxia* sp. and red squirrels *Sciurus vulgaris* at Abernathy forest, Strathespy. Forest Ecology and Management.

[ref-34] Wood S (2015). http://cran.r-project.org/web/packages=mgcv.

