# Supplementary material for: Difference on cone size preferences between two coniferous species by Great Spotted Woodpecker (Dendrocopos major)
Source: PeerJ. 2017 May 31;5:e3288. doi: 10.7717/peerj.3288 (PMC5455344; doi:10.7717/peerj.3288)
Supplement: Data S1 [file peerj-05-3288-s001.pdf]

Spruce cone measurement

anvil	prob.predation	length
1	0	63.22
1	0	64.34
1	0	69.62
1	0	76
1	0	76.02
1	0	85.85
1	0	86.79
1	0	86.88
1	0	87.08
1	0	88.08
1	0	88.21
1	0	89.45
1	0	89.66
1	0	92.99
1	0	96.29
1	0	96.36
1	0	97.02
1	0	97.26
1	0	97.36
1	0	97.82
1	0	98.22
1	0	98.57
1	0	98.79
1	0	99.04
1	0	100.12
1	0	101.05
1	0	101.27
1	0	101.44
1	0	101.82
1	0	101.83
1	0	102.19
1	0	102.88
1	0	102.9
1	0	103.1
1	0	103.14
1	0	103.49
1	0	103.71
1	0	104
1	0	104.35
1	0	105.13
1	0	105.14
1	0	105.43
1	0	105.47
1	0	105.53

1	0	105.63
1	0	105.68
1	0	106.87
1	0	106.95
1	0	106.96
1	0	106.98
1	0	107.19
1	0	107.43
1	0	107.48
1	0	107.74
1	0	107.8
1	0	107.81
1	0	108.14
1	0	108.22
1	0	108.36
1	0	108.37
1	0	108.37
1	0	108.9
1	0	108.95
1	0	109.09
1	0	109.25
1	0	109.7
1	0	109.91
1	0	110.07
1	0	110.09
1	0	110.14
1	0	110.31
1	0	110.35
1	0	110.49
1	0	110.69
1	0	111.09
1	0	111.39
1	0	111.42
1	0	111.42
1	0	111.45
1	0	111.7
1	0	111.73
1	0	111.73
1	0	111.89
1	0	112.04
1	0	112.34
1	0	112.37
1	0	112.77
1	0	112.9
1	0	113.63
1	0	113.89
1	0	116.08

1	0	117.7
1	0	117.77
1	0	117.92
1	0	118.83
1	0	119.2
1	0	119.39
1	0	119.44
1	0	119.49
1	0	119.61
1	0	119.89
1	0	119.95
1	0	120.4
1	0	120.56
1	0	120.92
1	0	121.17
1	0	121.39
1	0	121.64
1	0	121.8
1	0	122.02
1	0	122.07
1	0	122.44
1	0	122.5
1	0	122.96
1	0	123.11
1	0	123.31
1	0	123.36
1	0	123.36
1	0	123.74
1	0	123.77
1	0	124.6
1	0	124.85
1	0	124.91
1	0	125.03
1	0	125.08
1	0	125.15
1	0	125.62
1	0	125.95
1	0	126.07
1	0	126.88
1	0	126.95
1	0	127.06
1	0	127.6
1	0	127.75
1	0	127.8
1	0	128
1	0	128.35
1	0	128.43

1	0	128.62
1	0	128.71
1	0	129
1	0	130
1	0	130
1	0	130.99
1	0	131
1	0	131
1	0	131
1	0	133
1	0	134.18
1	0	134.21
1	0	134.87
1	0	135
1	0	135
1	0	137
1	0	139.13
1	0	139.57
1	0	144
1	0	145
1	0	79
1	0	83.14
1	1	84.28
1	1	89.57
1	1	91.24
1	1	91.8
1	1	92.74
1	1	92.76
1	1	93.48
1	1	93.74
1	1	93.98
1	1	94.83
1	1	94.83
1	1	94.98
1	1	95.05
1	1	95.62
1	1	96.7
1	1	97.18
1	1	97.23
1	1	97.29
1	1	98.34
1	1	98.73
1	1	98.98
1	1	99.22
1	1	99.27
1	1	99.43
1	1	100.01

1	1	100.57
1	1	100.82
1	1	101.16
1	1	101.18
1	1	101.24
1	1	101.41
1	1	101.75
1	1	102.6
1	1	102.67
1	1	102.94
1	1	103.04
1	1	103.5
1	1	103.63
1	1	103.74
1	1	103.76
1	1	103.92
1	1	103.94
1	1	104
1	1	104.1
1	1	104.31
1	1	104.63
1	1	104.76
1	1	104.85
1	1	104.9
1	1	106.11
1	1	106.27
1	1	106.32
1	1	106.4
1	1	106.44
1	1	106.51
1	1	106.51
1	1	106.61
1	1	106.69
1	1	106.93
1	1	107.21
1	1	107.39
1	1	108.05
1	1	108.1
1	1	108.17
1	1	108.23
1	1	108.49
1	1	108.54
1	1	108.64
1	1	108.74
1	1	109.41
1	1	109.47
1	1	110.09

1	1	110.34
1	1	110.37
1	1	110.54
1	1	110.76
1	1	110.92
1	1	110.92
1	1	110.96
1	1	111.26
1	1	111.67
1	1	112.35
1	1	112.48
1	1	112.54
1	1	112.68
1	1	113.35
1	1	113.45
1	1	113.67
1	1	113.7
1	1	113.71
1	1	113.89
1	1	113.91
1	1	113.98
1	1	114.05
1	1	114.16
1	1	114.27
1	1	114.29
1	1	114.37
1	1	114.39
1	1	114.42
1	1	114.43
1	1	114.47
1	1	114.54
1	1	114.76
1	1	114.97
1	1	115.01
1	1	115.03
1	1	115.06
1	1	115.1
1	1	115.13
1	1	115.18
1	1	115.29
1	1	115.34
1	1	115.43
1	1	115.5
1	1	115.61
1	1	115.63
1	1	115.71
1	1	115.8

1	1	115.85
1	1	115.86
1	1	115.93
1	1	115.95
1	1	116.01
1	1	116.14
1	1	116.21
1	1	116.29
1	1	116.73
1	1	116.83
1	1	116.91
1	1	116.97
1	1	117.06
1	1	117.07
1	1	117.14
1	1	117.24
1	1	117.41
1	1	118.16
1	1	119.05
1	1	119.28
1	1	119.35
1	1	119.42
1	1	119.59
1	1	119.62
1	1	119.82
1	1	120.75
1	1	121.16
1	1	121.87
1	1	121.91
1	1	121.92
1	1	122.05
1	1	122.49
1	1	123
1	1	123.26
1	1	123.48
1	1	123.74
1	1	124.65
1	1	124.71
1	1	124.91
1	1	125.61
1	1	127.15
1	1	127.46
1	1	128.03
1	1	128.75
1	1	128.91
1	1	129
1	1	129.06

1	1	129.13
1	1	129.34
1	1	132.15
1	1	135.06
1	1	136.04
1	1	137.21
1	1	137.51
2	0	74.97
2	0	76
2	0	76.86
2	0	80.41
2	0	81.75
2	0	81.87
2	0	82.09
2	0	82.12
2	0	82.28
2	0	82.38
2	0	82.72
2	0	83.8
2	0	84.77
2	0	85.17
2	0	85.81
2	0	85.84
2	0	85.92
2	0	85.92
2	0	85.95
2	0	86.14
2	0	86.34
2	0	87.07
2	0	87.09
2	0	87.21
2	0	87.25
2	0	87.5
2	0	87.57
2	0	87.58
2	0	87.69
2	0	87.71
2	0	87.78
2	0	87.83
2	0	87.95
2	0	87.98
2	0	88.41
2	0	92.73
2	0	93.12
2	0	93.2
2	0	93.63
2	0	93.64

2	0	93.75
2	0	93.77
2	0	93.95
2	0	94.71
2	0	95.18
2	0	95.56
2	0	95.57
2	0	95.74
2	0	96.11
2	0	96.32
2	0	96.72
2	0	96.74
2	0	96.77
2	0	96.83
2	0	96.83
2	0	96.95
2	0	97.4
2	0	97.85
2	0	97.89
2	0	97.91
2	0	98
2	0	98.08
2	0	98.18
2	0	98.25
2	0	98.91
2	0	98.98
2	0	99.05
2	0	99.15
2	0	99.45
2	0	99.75
2	0	100.16
2	0	100.21
2	0	100.32
2	0	100.4
2	0	100.43
2	0	100.55
2	0	100.87
2	0	100.95
2	0	101.11
2	0	101.12
2	0	101.19
2	0	101.25
2	0	101.3
2	0	101.34
2	0	101.38
2	0	101.38
2	0	101.67

2	0	101.96
2	0	102.22
2	0	102.3
2	0	102.36
2	0	102.68
2	0	102.75
2	0	103.04
2	0	103.08
2	0	103.32
2	0	103.39
2	0	103.43
2	0	103.47
2	0	103.92
2	0	104.3
2	0	104.31
2	0	104.43
2	0	104.55
2	0	104.66
2	0	104.73
2	0	104.77
2	0	105.12
2	0	105.15
2	0	105.15
2	0	105.24
2	0	105.31
2	0	105.32
2	0	105.37
2	0	105.38
2	0	105.39
2	0	105.65
2	0	105.71
2	0	105.76
2	0	105.81
2	0	105.84
2	0	105.87
2	0	105.89
2	0	106.68
2	0	106.7
2	0	106.94
2	0	107.03
2	0	107.11
2	0	107.13
2	0	107.27
2	0	107.44
2	0	107.46
2	0	107.54
2	0	107.56

2	0	107.73
2	0	107.74
2	0	107.88
2	0	107.92
2	0	108.01
2	0	108.26
2	0	108.4
2	0	108.59
2	0	108.59
2	0	108.6
2	0	108.72
2	0	108.89
2	0	108.91
2	0	109.02
2	0	109.15
2	0	109.3
2	0	109.4
2	0	109.5
2	0	109.51
2	0	109.53
2	0	109.56
2	0	109.68
2	0	109.92
2	0	110.28
2	0	110.39
2	0	110.47
2	0	110.61
2	0	111.42
2	0	111.58
2	0	111.62
2	0	111.62
2	0	111.75
2	0	111.81
2	0	111.85
2	0	111.92
2	0	112.18
2	0	112.55
2	0	112.59
2	0	112.97
2	0	113.16
2	0	113.71
2	0	113.73
2	0	113.85
2	0	113.86
2	0	117.46
2	0	117.95
2	0	118.29

2	0	119.04
2	0	119.1
2	0	119.16
2	0	119.21
2	0	119.33
2	0	121.24
2	0	121.35
2	0	121.66
2	0	121.81
2	0	121.86
2	0	121.97
2	0	122.01
2	0	122.81
2	0	122.86
2	0	123.86
2	0	123.86
2	0	124
2	0	124.43
2	0	125.13
2	0	126.24
2	0	126.82
2	0	132.24
2	0	150.6
2	1	89.36
2	1	90.11
2	1	90.13
2	1	90.28
2	1	91.76
2	1	91.95
2	1	92.31
2	1	93
2	1	79.05
2	1	79.8
2	1	80
2	1	80
2	1	80.41
2	1	81.36
2	1	83.41
2	1	83.56
2	1	83.78
2	1	84.01
2	1	84.15
2	1	84.99
2	1	85.41
2	1	85.45
2	1	85.52
2	1	85.57

2	1	88.76
2	1	88.94
2	1	88.98
2	1	89.25
2	1	89.28
2	1	89.48
2	1	89.66
2	1	89.7
2	1	90.27
2	1	90.39
2	1	90.42
2	1	90.45
2	1	90.48
2	1	90.61
2	1	90.91
2	1	90.92
2	1	90.98
2	1	91.23
2	1	91.24
2	1	91.47
2	1	91.51
2	1	91.93
2	1	92.18
2	1	92.28
2	1	92.37
2	1	92.44
2	1	92.46
2	1	92.82
2	1	93.2
2	1	93.22
2	1	93.26
2	1	93.26
2	1	93.28
2	1	93.3
2	1	93.41
2	1	93.46
2	1	93.66
2	1	93.76
2	1	94.2
2	1	94.3
2	1	94.57
2	1	94.68
2	1	94.75
2	1	94.81
2	1	94.83
2	1	94.88
2	1	95.12

2	1	95.33
2	1	95.49
2	1	95.77
2	1	95.85
2	1	95.97
2	1	96.19
2	1	96.21
2	1	96.31
2	1	96.4
2	1	96.53
2	1	96.85
2	1	96.89
2	1	96.96
2	1	97.2
2	1	97.44
2	1	97.69
2	1	97.84
2	1	97.95
2	1	98.01
2	1	98.05
2	1	98.11
2	1	98.5
2	1	98.58
2	1	98.79
2	1	99.06
2	1	99.65
2	1	99.68
2	1	99.76
2	1	99.82
2	1	99.86
2	1	99.93
2	1	100.06
2	1	100.1
2	1	100.3
2	1	100.77
2	1	100.79
2	1	100.8
2	1	101.41
2	1	101.58
2	1	101.58
2	1	101.67
2	1	101.75
2	1	101.97
2	1	102.24
2	1	102.41
2	1	102.85
2	1	102.85

2	1	102.96
2	1	103.07
2	1	103.44
2	1	103.57
2	1	104.12
2	1	104.91
2	1	105.96
2	1	106.21
2	1	106.28
2	1	106.57
2	1	106.73
2	1	106.88
2	1	106.98
2	1	107.14
2	1	107.68
2	1	108.39
2	1	109.61
2	1	109.62
2	1	109.98
2	1	110.58
2	1	111.2
2	1	111.39
2	1	112.12
2	1	112.16
2	1	112.32
2	1	112.4
2	1	112.52
2	1	112.77
2	1	112.93
2	1	113.19
2	1	113.34
2	1	113.62
2	1	113.71
2	1	114.29
2	1	114.43
2	1	114.74
2	1	114.85
2	1	114.9
2	1	114.91
2	1	115.58
2	1	116.05
2	1	116.2
2	1	116.33
2	1	116.39
2	1	116.62
2	1	116.73
2	1	116.74

2	1	116.96
2	1	117.07
2	1	117.17
2	1	118.04
2	1	118.21
2	1	118.83
2	1	119.56
2	1	119.64
2	1	120.89
2	1	121.88
2	1	122.4
2	1	124.01
2	1	126.61
2	1	128
2	1	134.16
2	1	135.26
3	0	74.73
3	0	76.81
3	0	82.64
3	0	82.73
3	0	82.86
3	0	84.54
3	0	86.3
3	0	86.63
3	0	86.97
3	0	87.5
3	0	87.54
3	0	87.89
3	0	88.6
3	0	89.6
3	0	91.27
3	0	93.86
3	0	95.19
3	0	95.82
3	0	98.71
3	0	99.12
3	0	100.06
3	0	100.18
3	0	102
3	0	102.02
3	0	102.74
3	0	103.2
3	0	103.6
3	0	105.04
3	0	105.1
3	0	105.2
3	0	105.36

3	0	105.44
3	0	105.59
3	0	106.99
3	0	108.38
3	0	109.19
3	0	112.24
3	0	112.61
3	0	112.9
3	0	116.03
3	0	117.43
3	0	117.71
3	0	117.75
3	0	124.38
3	1	76.87
3	1	77
3	1	77
3	1	78.11
3	1	78.31
3	1	78.91
3	1	79.02
3	1	79.36
3	1	79.58
3	1	83.12
3	1	83.19
3	1	83.26
3	1	83.71
3	1	84.44
3	1	84.79
3	1	85.41
3	1	85.59
3	1	89.15
3	1	89.83
3	1	89.89
3	1	90.47
3	1	90.65
3	1	90.67
3	1	91.13
3	1	91.2
3	1	91.86
3	1	92.25
3	1	92.56
3	1	92.59
3	1	92.89
3	1	93.03
3	1	93.03
3	1	93.85
3	1	94.15

3	1	94.17
3	1	94.38
3	1	94.68
3	1	94.72
3	1	95.2
3	1	95.2
3	1	95.47
3	1	95.62
3	1	96.4
3	1	96.72
3	1	96.96
3	1	97.6
3	1	97.6
3	1	97.93
3	1	98.02
3	1	98.45
3	1	98.52
3	1	98.65
3	1	98.85
3	1	99.34
3	1	99.47
3	1	99.77
3	1	100.25
3	1	100.37
3	1	100.38
3	1	100.76
3	1	100.95
3	1	100.98
3	1	101
3	1	101.24
3	1	101.43
3	1	102.09
3	1	102.25
3	1	102.41
3	1	102.5
3	1	102.67
3	1	102.74
3	1	103.03
3	1	103.4
3	1	103.5
3	1	103.67
3	1	103.69
3	1	103.75
3	1	103.75
3	1	103.94
3	1	103.94
3	1	104.15

3	1	104.37
3	1	104.37
3	1	104.39
3	1	104.47
3	1	104.49
3	1	104.61
3	1	104.77
3	1	104.82
3	1	105.98
3	1	106.15
3	1	106.3
3	1	106.46
3	1	106.49
3	1	106.78
3	1	106.83
3	1	106.88
3	1	107
3	1	107.11
3	1	107.13
3	1	107.21
3	1	107.43
3	1	107.62
3	1	107.7
3	1	108.26
3	1	108.33
3	1	108.51
3	1	108.6
3	1	109.22
3	1	109.51
3	1	109.64
3	1	110.12
3	1	110.38
3	1	110.82
3	1	111.1
3	1	111.15
3	1	111.32
3	1	111.37
3	1	111.4
3	1	112.2
3	1	112.23
3	1	112.3
3	1	112.33
3	1	112.5
3	1	112.6
3	1	112.68
3	1	112.84
3	1	113.06

3	1	113.15
3	1	113.25
3	1	113.53
3	1	113.78
3	1	113.93
3	1	113.95
3	1	114.2
3	1	114.51
3	1	114.52
3	1	114.55
3	1	114.59
3	1	114.65
3	1	114.99
3	1	115.03
3	1	115.79
3	1	115.97
3	1	116.12
3	1	116.59
3	1	116.66
3	1	116.76
3	1	116.83
3	1	117.05
3	1	117.34
3	1	117.35
3	1	117.4
3	1	117.45
3	1	117.92
3	1	118.05
3	1	118.24
3	1	118.35
3	1	118.37
3	1	118.38
3	1	118.82
3	1	118.94
3	1	119.07
3	1	119.32
3	1	119.37
3	1	119.52
3	1	119.83
3	1	120.1
3	1	120.6
3	1	120.6
3	1	121.27
3	1	121.66
3	1	122.27
3	1	122.95
3	1	125.31

3	1	126.41
3	1	127.33
3	1	134.41
3	1	136.4
4	0	91.97
4	0	94.27
4	0	96.11
4	0	97.11
4	0	98.31
4	0	99.54
4	0	100.09
4	0	100.89
4	0	102.42
4	0	103.96
4	0	104.16
4	0	104.32
4	0	105.14
4	0	105.31
4	0	105.61
4	0	105.63
4	0	105.66
4	0	107.3
4	0	107.44
4	0	108.06
4	0	108.15
4	0	108.27
4	0	108.4
4	0	108.67
4	0	109.02
4	0	109.04
4	0	109.18
4	0	109.23
4	0	109.89
4	0	110.09
4	0	110.81
4	0	110.96
4	0	111.07
4	0	111.4
4	0	112.63
4	0	112.76
4	0	113.27
4	0	113.39
4	0	113.42
4	0	113.56
4	0	113.87
4	0	117.5
4	0	117.61

4	0	117.72
4	0	117.73
4	0	117.78
4	0	117.9
4	0	117.96
4	0	117.99
4	0	118.16
4	0	118.19
4	0	118.6
4	0	118.84
4	0	118.96
4	0	119.13
4	0	119.31
4	0	119.35
4	0	119.54
4	0	119.71
4	0	119.75
4	0	119.78
4	0	119.9
4	0	120.42
4	0	120.71
4	0	121.04
4	0	121.27
4	0	121.97
4	0	122.08
4	0	122.67
4	0	123.05
4	0	123.38
4	0	123.5
4	0	123.6
4	0	123.76
4	0	124.78
4	0	124.8
4	0	125.17
4	0	125.4
4	0	126.15
4	0	126.2
4	0	126.33
4	0	127.18
4	0	127.43
4	0	127.63
4	0	127.72
4	0	128.13
4	0	129
4	0	129
4	0	130
4	0	130.54

4	0	131.5
4	0	132
4	0	133.26
4	0	133.57
4	0	136
4	0	136.4
4	0	136.63
4	0	136.68
4	0	137
4	0	153
4	1	92.68
4	1	93.84
4	1	94.53
4	1	95.33
4	1	96.12
4	1	96.18
4	1	96.62
4	1	98
4	1	98.55
4	1	99.05
4	1	99.76
4	1	100.3
4	1	100.35
4	1	100.59
4	1	101.27
4	1	101.29
4	1	101.77
4	1	102.27
4	1	102.61
4	1	102.83
4	1	102.87
4	1	103.02
4	1	103.33
4	1	103.7
4	1	104.08
4	1	104.7
4	1	105.92
4	1	106.25
4	1	106.27
4	1	107.86
4	1	107.92
4	1	108.36
4	1	109.06
4	1	109.33
4	1	109.5
4	1	109.56
4	1	110.16

4	1	110.22
4	1	110.37
4	1	110.97
4	1	111.02
4	1	111.37
4	1	111.41
4	1	112.19
4	1	112.25
4	1	112.52
4	1	113.09
4	1	113.26
4	1	113.8
4	1	113.84
4	1	114.37
4	1	114.63
4	1	114.66
4	1	114.71
4	1	114.79
4	1	114.92
4	1	115.07
4	1	115.22
4	1	115.47
4	1	115.65
4	1	115.85
4	1	115.94
4	1	116.15
4	1	116.2
4	1	116.28
4	1	116.46
4	1	116.63
4	1	116.77
4	1	116.83
4	1	117.04
4	1	117.19
4	1	117.33
4	1	117.4
4	1	117.41
4	1	118.56
4	1	118.66
4	1	118.84
4	1	119.53
4	1	119.65
4	1	119.75
4	1	119.75
4	1	121.58
4	1	122.22
4	1	122.59

4	1	129.32
4	1	130.97
4	1	134.1
5	0	83.1
5	0	83.28
5	0	86.61
5	0	87.41
5	0	87.57
5	0	87.98
5	0	90.11
5	0	90.13
5	0	90.48
5	0	91.02
5	0	91.24
5	0	92.08
5	0	93.92
5	0	94.26
5	0	94.6
5	0	94.9
5	0	94.99
5	0	95.85
5	0	96.02
5	0	96.24
5	0	96.46
5	0	97.73
5	0	97.96
5	0	98.53
5	0	98.72
5	0	99.03
5	0	99.45
5	0	99.75
5	0	100.46
5	0	101.38
5	0	101.82
5	0	101.98
5	0	102.61
5	0	103.09
5	0	103.21
5	0	103.26
5	0	103.53
5	0	103.67
5	0	103.76
5	0	103.98
5	0	104.53
5	0	104.64
5	0	105.02
5	0	105.07

5	0	105.11
5	0	105.17
5	0	105.21
5	0	105.23
5	0	105.33
5	0	105.37
5	0	105.41
5	0	105.81
5	0	105.83
5	0	105.83
5	0	105.9
5	0	105.9
5	0	105.91
5	0	105.91
5	0	106.29
5	0	106.59
5	0	107.12
5	0	107.43
5	0	107.43
5	0	107.7
5	0	108.74
5	0	108.8
5	0	108.93
5	0	108.97
5	0	109.07
5	0	109.36
5	0	109.69
5	0	110.64
5	0	110.85
5	0	110.9
5	0	110.93
5	0	111.21
5	0	111.34
5	0	111.56
5	0	111.75
5	0	111.85
5	0	112.02
5	0	112.3
5	0	112.72
5	0	113.03
5	0	113.06
5	0	117.42
5	0	117.47
5	0	117.67
5	0	117.91
5	0	118.38
5	0	118.84

5	0	118.9
5	0	119.06
5	0	119.22
5	0	119.53
5	0	119.6
5	0	119.79
5	0	120.06
5	0	120.35
5	0	120.43
5	0	120.54
5	0	120.63
5	0	121.54
5	0	121.56
5	0	121.63
5	0	121.86
5	0	121.86
5	0	121.91
5	0	122.14
5	0	122.17
5	0	122.27
5	0	122.44
5	0	122.82
5	0	122.97
5	0	122.99
5	0	123.12
5	0	123.81
5	0	123.82
5	0	123.91
5	0	124.37
5	0	124.65
5	0	125.18
5	0	125.32
5	0	125.9
5	0	126.13
5	0	127.28
5	0	127.47
5	0	128
5	0	128
5	0	128
5	0	128.03
5	0	128.18
5	0	128.35
5	0	129.26
5	0	129.43
5	0	129.75
5	0	129.76
5	0	130.82

5	0	131
5	0	131
5	0	131.01
5	0	131.32
5	0	132.71
5	0	132.72
5	0	133.06
5	0	133.43
5	0	134.13
5	0	134.25
5	0	134.47
5	0	134.87
5	0	136
5	0	136
5	0	136.03
5	0	136.32
5	0	136.9
5	0	137
5	0	137.72
5	0	138.24
5	0	138.9
5	0	139.81
5	0	140
5	0	140
5	0	140
5	0	140.54
5	0	140.79
5	0	140.97
5	0	141
5	0	142.96
5	0	144
5	0	145.76
5	0	146.22
5	0	148.49
5	0	149.82
5	0	150.81
5	0	151
5	0	152.72
5	0	153.1
5	0	154.31
5	1	77.08
5	1	79.88
5	1	81
5	1	89.2
5	1	89.75
5	1	90.36
5	1	90.65

5	1	90.7
5	1	91.71
5	1	92.08
5	1	93.81
5	1	94.38
5	1	94.49
5	1	94.52
5	1	94.63
5	1	94.85
5	1	95
5	1	95.11
5	1	95.96
5	1	96.86
5	1	96.94
5	1	98.81
5	1	98.9
5	1	99.95
5	1	100.02
5	1	100.12
5	1	100.13
5	1	100.6
5	1	100.75
5	1	101.03
5	1	101.14
5	1	101.23
5	1	101.63
5	1	101.71
5	1	101.72
5	1	101.81
5	1	101.87
5	1	101.93
5	1	102.88
5	1	103.02
5	1	103.05
5	1	103.58
5	1	103.7
5	1	103.71
5	1	103.9
5	1	103.98
5	1	104.07
5	1	104.11
5	1	104.12
5	1	104.3
5	1	104.59
5	1	104.84
5	1	105.99
5	1	106.04

5	1	106.05
5	1	106.05
5	1	106.12
5	1	106.31
5	1	106.32
5	1	106.5
5	1	106.51
5	1	106.73
5	1	106.85
5	1	106.86
5	1	106.97
5	1	107.01
5	1	107.08
5	1	107.18
5	1	107.43
5	1	107.66
5	1	107.69
5	1	107.72
5	1	107.75
5	1	107.81
5	1	107.91
5	1	108
5	1	108.04
5	1	108.06
5	1	108.21
5	1	108.46
5	1	108.61
5	1	108.79
5	1	109.15
5	1	109.17
5	1	109.18
5	1	109.21
5	1	109.41
5	1	109.47
5	1	109.55
5	1	109.7
5	1	109.72
5	1	109.74
5	1	109.74
5	1	109.75
5	1	109.98
5	1	110.01
5	1	110.13
5	1	110.13
5	1	110.16
5	1	110.23
5	1	110.25

5	1	110.46
5	1	110.46
5	1	110.54
5	1	110.57
5	1	110.61
5	1	110.68
5	1	110.91
5	1	110.97
5	1	111.14
5	1	111.15
5	1	111.17
5	1	111.61
5	1	111.92
5	1	112.13
5	1	112.32
5	1	112.35
5	1	112.46
5	1	112.49
5	1	112.58
5	1	112.6
5	1	112.66
5	1	112.66
5	1	112.68
5	1	112.7
5	1	112.78
5	1	112.87
5	1	113.12
5	1	113.13
5	1	113.25
5	1	113.33
5	1	113.61
5	1	113.62
5	1	113.7
5	1	113.79
5	1	113.82
5	1	113.87
5	1	113.91
5	1	114.24
5	1	114.38
5	1	114.41
5	1	114.52
5	1	114.55
5	1	114.57
5	1	114.66
5	1	114.67
5	1	114.7
5	1	114.79

5	1	114.83
5	1	114.87
5	1	114.89
5	1	114.94
5	1	115.1
5	1	115.12
5	1	115.2
5	1	115.25
5	1	115.26
5	1	115.27
5	1	115.29
5	1	115.5
5	1	115.51
5	1	115.52
5	1	115.54
5	1	115.58
5	1	115.65
5	1	116.17
5	1	116.21
5	1	116.33
5	1	116.34
5	1	116.37
5	1	116.4
5	1	116.41
5	1	116.48
5	1	116.52
5	1	116.72
5	1	116.79
5	1	116.8
5	1	116.83
5	1	116.96
5	1	116.97
5	1	117.07
5	1	117.07
5	1	117.07
5	1	117.13
5	1	117.14
5	1	117.19
5	1	117.22
5	1	117.35
5	1	117.36
5	1	117.4
5	1	117.44
5	1	117.52
5	1	117.59
5	1	117.7
5	1	117.76

5	1	117.91
5	1	118.16
5	1	118.23
5	1	118.64
5	1	118.83
5	1	118.93
5	1	119.47
5	1	119.63
5	1	119.68
5	1	119.77
5	1	119.97
5	1	120.14
5	1	120.46
5	1	120.46
5	1	120.53
5	1	120.61
5	1	120.8
5	1	120.82
5	1	120.87
5	1	120.94
5	1	121.13
5	1	121.18
5	1	121.38
5	1	121.46
5	1	121.65
5	1	121.8
5	1	121.89
5	1	122.19
5	1	122.48
5	1	122.58
5	1	122.63
5	1	122.71
5	1	122.72
5	1	122.91
5	1	123.02
5	1	123.36
5	1	123.71
5	1	124.01
5	1	124.09
5	1	124.43
5	1	124.69
5	1	124.82
5	1	124.88
5	1	124.94
5	1	125.05
5	1	125.05
5	1	125.18

5	1	125.29
5	1	125.34
5	1	125.4
5	1	125.6
5	1	125.72
5	1	125.91
5	1	126.11
5	1	126.15
5	1	126.18
5	1	126.41
5	1	126.6
5	1	126.61
5	1	126.68
5	1	126.81
5	1	127.07
5	1	127.23
5	1	127.55
5	1	127.63
5	1	127.83
5	1	127.87
5	1	128
5	1	128
5	1	128
5	1	128.24
5	1	128.57
5	1	128.79
5	1	129
5	1	129.04
5	1	129.24
5	1	129.37
5	1	129.4
5	1	129.53
5	1	129.68
5	1	130.6
5	1	131
5	1	131
5	1	131.01
5	1	131.54
5	1	131.54
5	1	132
5	1	132.32
5	1	132.41
5	1	133
5	1	133
5	1	133.24
5	1	134
5	1	135

5	1	135.85
5	1	137
5	1	161
5	1	161
5	1	162.54
5	1	163.71
5	1	163.94
6	0	87.55
6	0	87.67
6	0	87.89
6	0	88.35
6	0	89.15
6	0	90.01
6	0	90.06
6	0	90.17
6	0	90.61
6	0	91.08
6	0	91.5
6	0	91.55
6	0	91.82
6	0	92.32
6	0	92.71
6	0	92.92
6	0	93.3
6	0	93.48
6	0	93.96
6	0	94.56
6	0	94.86
6	0	95.34
6	0	95.7
6	0	95.93
6	0	96.43
6	0	96.46
6	0	96.59
6	0	96.64
6	0	96.72
6	0	96.81
6	0	96.91
6	0	97.2
6	0	97.52
6	0	97.56
6	0	97.58
6	0	97.63
6	0	97.9
6	0	98.25
6	0	99.4
6	0	99.42

6	0	99.57
6	0	99.62
6	0	100.38
6	0	100.4
6	0	100.86
6	0	100.87
6	0	100.99
6	0	101.02
6	0	101.19
6	0	101.21
6	0	101.26
6	0	101.43
6	0	101.86
6	0	101.87
6	0	102.06
6	0	102.33
6	0	102.43
6	0	102.45
6	0	102.65
6	0	102.66
6	0	102.73
6	0	102.77
6	0	102.77
6	0	102.93
6	0	102.95
6	0	103.13
6	0	103.21
6	0	103.26
6	0	103.52
6	0	103.7
6	0	103.93
6	0	104.07
6	0	104.17
6	0	104.26
6	0	104.43
6	0	104.65
6	0	104.96
6	0	104.98
6	0	105.02
6	0	105.08
6	0	105.11
6	0	105.24
6	0	105.26
6	0	105.27
6	0	105.41
6	0	105.52
6	0	105.54

6	0	105.58
6	0	105.7
6	0	105.71
6	0	105.74
6	0	105.75
6	0	105.78
6	0	105.79
6	0	105.81
6	0	105.82
6	0	105.82
6	0	106.9
6	0	107.29
6	0	107.32
6	0	107.68
6	0	107.81
6	0	107.93
6	0	108.06
6	0	108.13
6	0	108.19
6	0	108.73
6	0	108.95
6	0	109.06
6	0	109.11
6	0	109.37
6	0	109.44
6	0	109.78
6	0	109.94
6	0	110.35
6	0	110.37
6	0	110.88
6	0	110.89
6	0	111.5
6	0	111.6
6	0	111.7
6	0	111.72
6	0	111.73
6	0	111.79
6	0	112.33
6	0	112.44
6	0	112.54
6	0	113.14
6	0	113.33
6	0	113.48
6	0	113.5
6	0	114.16
6	0	117.5
6	0	117.63

6	0	117.71
6	0	118.1
6	0	118.36
6	0	118.46
6	0	118.58
6	0	119.93
6	0	120.08
6	0	120.11
6	0	121.04
6	0	121.41
6	0	121.69
6	0	123.52
6	0	123.78
6	0	124.12
6	0	124.27
6	0	124.82
6	0	124.97
6	0	125.34
6	0	125.4
6	0	126.78
6	0	127.15
6	0	127.63
6	0	127.68
6	0	129
6	0	129.71
6	0	130
6	0	130.4
6	0	131
6	0	136.88
6	0	138.38
6	0	138.62
6	0	139.65
6	0	140.12
6	0	141
6	0	141.07
6	0	141.96
6	0	144.31
6	0	152.15
6	1	79
6	1	79.48
6	1	83.31
6	1	84.97
6	1	85.5
6	1	89.17
6	1	89.24
6	1	90.28
6	1	90.53

6	1	90.53
6	1	90.54
6	1	90.82
6	1	90.95
6	1	91.02
6	1	91.51
6	1	92.52
6	1	92.56
6	1	93.76
6	1	93.79
6	1	93.97
6	1	94.07
6	1	94.38
6	1	95.19
6	1	95.85
6	1	96.28
6	1	96.4
6	1	96.42
6	1	96.61
6	1	96.69
6	1	97.49
6	1	98.19
6	1	98.36
6	1	98.55
6	1	98.64
6	1	99.3
6	1	99.32
6	1	99.33
6	1	99.62
6	1	99.62
6	1	99.97
6	1	100.21
6	1	100.38
6	1	100.41
6	1	100.48
6	1	100.72
6	1	101.11
6	1	101.12
6	1	101.5
6	1	101.51
6	1	101.95
6	1	102.15
6	1	102.27
6	1	102.29
6	1	102.29
6	1	102.48
6	1	102.49

6	1	102.66
6	1	102.71
6	1	102.93
6	1	102.98
6	1	103.03
6	1	103.21
6	1	103.24
6	1	103.25
6	1	103.39
6	1	103.74
6	1	103.86
6	1	103.92
6	1	104.14
6	1	104.15
6	1	104.19
6	1	104.37
6	1	104.5
6	1	104.58
6	1	104.74
6	1	104.76
6	1	104.84
6	1	104.86
6	1	106.03
6	1	106.03
6	1	106.03
6	1	106.11
6	1	106.12
6	1	106.22
6	1	106.3
6	1	106.31
6	1	106.38
6	1	106.45
6	1	106.48
6	1	106.48
6	1	106.5
6	1	106.53
6	1	106.53
6	1	106.58
6	1	106.61
6	1	106.65
6	1	106.73
6	1	106.86
6	1	107.24
6	1	107.26
6	1	107.48
6	1	107.93
6	1	107.95

6	1	108.3
6	1	108.57
6	1	108.84
6	1	108.89
6	1	108.99
6	1	109.09
6	1	109.15
6	1	109.16
6	1	109.62
6	1	109.76
6	1	109.82
6	1	109.84
6	1	110.36
6	1	110.36
6	1	110.36
6	1	110.48
6	1	110.69
6	1	110.92
6	1	110.99
6	1	111.26
6	1	111.38
6	1	111.4
6	1	111.49
6	1	111.68
6	1	111.77
6	1	111.79
6	1	111.82
6	1	111.9
6	1	111.95
6	1	112.36
6	1	112.71
6	1	112.73
6	1	112.74
6	1	112.87
6	1	113.01
6	1	113.13
6	1	113.18
6	1	113.42
6	1	113.45
6	1	113.5
6	1	113.52
6	1	113.58
6	1	113.85
6	1	113.89
6	1	113.92
6	1	114.1
6	1	114.1

6	1	114.12
6	1	114.23
6	1	114.28
6	1	114.28
6	1	114.53
6	1	114.53
6	1	114.54
6	1	114.56
6	1	114.68
6	1	114.71
6	1	114.73
6	1	114.78
6	1	114.83
6	1	114.91
6	1	115.03
6	1	115.09
6	1	115.22
6	1	115.44
6	1	115.45
6	1	115.49
6	1	115.55
6	1	115.56
6	1	115.65
6	1	116.01
6	1	116.05
6	1	116.2
6	1	116.27
6	1	116.28
6	1	116.33
6	1	116.35
6	1	116.4
6	1	116.5
6	1	116.74
6	1	116.74
6	1	116.8
6	1	116.83
6	1	116.9
6	1	116.99
6	1	117.06
6	1	117.28
6	1	117.33
6	1	117.35
6	1	117.38
6	1	117.43
6	1	117.45
6	1	117.52
6	1	117.56

6	1	117.66
6	1	117.75
6	1	117.77
6	1	117.81
6	1	118
6	1	118.01
6	1	118.03
6	1	118.12
6	1	118.12
6	1	118.34
6	1	118.36
6	1	118.45
6	1	118.52
6	1	118.55
6	1	118.59
6	1	118.63
6	1	119.02
6	1	119.15
6	1	119.28
6	1	119.38
6	1	119.4
6	1	119.48
6	1	119.57
6	1	119.62
6	1	119.69
6	1	119.7
6	1	119.97
6	1	120
6	1	120.01
6	1	120.51
6	1	120.71
6	1	120.71
6	1	120.75
6	1	120.85
6	1	120.91
6	1	120.97
6	1	121
6	1	121.26
6	1	121.81
6	1	122.34
6	1	122.49
6	1	122.5
6	1	122.52
6	1	122.6
6	1	122.91
6	1	123.04
6	1	123.17

6	1	123.34
6	1	123.48
6	1	123.55
6	1	124.47
6	1	124.54
6	1	125.59
6	1	125.73
6	1	125.75
6	1	125.94
6	1	125.95
6	1	126.17
6	1	126.28
6	1	126.69
6	1	126.79
6	1	126.91
6	1	126.95
6	1	127.28
6	1	127.32
6	1	127.36
6	1	127.39
6	1	127.5
6	1	127.58
6	1	127.72
6	1	128
6	1	128
6	1	128.82
6	1	129
6	1	129.18
6	1	129.71
6	1	129.93
6	1	130
6	1	130
6	1	130.18
6	1	130.43
6	1	130.46
6	1	130.71
6	1	131
6	1	131.82
6	1	131.88
6	1	132.09
6	1	132.56
6	1	133
6	1	133.66
6	1	134
6	1	134.01
6	1	134.76
6	1	135.32

6	1	136
6	1	136
6	1	136.1
6	1	137
6	1	137.35
6	1	137.56
7	0	82.31
7	0	82.53
7	0	82.57
7	0	82.71
7	0	82.99
7	0	83.04
7	0	83.17
7	0	85.87
7	0	86.02
7	0	86.2
7	0	86.21
7	0	86.21
7	0	86.26
7	0	86.35
7	0	86.59
7	0	86.64
7	0	86.81
7	0	86.92
7	0	86.96
7	0	87.17
7	0	87.23
7	0	87.37
7	0	87.75
7	0	88.03
7	0	88.2
7	0	88.44
7	0	88.48
7	0	90.07
7	0	90.11
7	0	90.19
7	0	90.87
7	0	91.01
7	0	91.42
7	0	91.54
7	0	92.36
7	0	92.46
7	0	92.48
7	0	92.64
7	0	92.78
7	0	93.37
7	0	93.57

7	0	93.6
7	0	93.63
7	0	93.92
7	0	94.24
7	0	94.24
7	0	94.28
7	0	94.28
7	0	94.32
7	0	94.5
7	0	94.58
7	0	95.49
7	0	96.14
7	0	96.15
7	0	96.18
7	0	96.24
7	0	96.66
7	0	96.74
7	0	96.74
7	0	96.88
7	0	96.97
7	0	97.37
7	0	97.45
7	0	97.46
7	0	97.58
7	0	97.68
7	0	97.7
7	0	97.74
7	0	98.07
7	0	98.13
7	0	98.41
7	0	99.05
7	0	99.47
7	0	99.82
7	0	99.85
7	0	99.93
7	0	100.1
7	0	100.15
7	0	100.25
7	0	100.3
7	0	100.35
7	0	100.37
7	0	100.54
7	0	100.61
7	0	100.66
7	0	100.89
7	0	101.35
7	0	101.41

7	0	101.61
7	0	101.79
7	0	101.84
7	0	101.85
7	0	102.02
7	0	102.15
7	0	102.32
7	0	102.33
7	0	102.36
7	0	102.49
7	0	102.69
7	0	102.84
7	0	103.15
7	0	103.26
7	0	103.27
7	0	103.36
7	0	103.4
7	0	103.4
7	0	103.59
7	0	103.83
7	0	103.85
7	0	103.98
7	0	104.24
7	0	104.27
7	0	104.55
7	0	104.61
7	0	104.8
7	0	105.02
7	0	105.07
7	0	105.08
7	0	105.11
7	0	105.17
7	0	105.22
7	0	105.22
7	0	105.23
7	0	105.27
7	0	105.33
7	0	105.43
7	0	105.46
7	0	105.46
7	0	105.47
7	0	105.55
7	0	105.55
7	0	105.6
7	0	105.61
7	0	105.63
7	0	105.69

7	0	105.73
7	0	105.83
7	0	105.85
7	0	105.91
7	0	106.23
7	0	106.27
7	0	106.63
7	0	106.63
7	0	106.64
7	0	106.66
7	0	106.69
7	0	106.73
7	0	106.77
7	0	106.96
7	0	107.05
7	0	107.13
7	0	107.13
7	0	107.24
7	0	107.28
7	0	107.29
7	0	107.32
7	0	107.33
7	0	107.42
7	0	107.43
7	0	107.7
7	0	107.73
7	0	107.73
7	0	107.75
7	0	107.88
7	0	107.88
7	0	107.99
7	0	108.35
7	0	108.5
7	0	108.56
7	0	108.6
7	0	108.87
7	0	109.03
7	0	109.11
7	0	109.11
7	0	109.12
7	0	109.12
7	0	109.12
7	0	109.18
7	0	109.21
7	0	109.4
7	0	109.41
7	0	109.47

7	0	109.68
7	0	109.79
7	0	109.83
7	0	109.83
7	0	109.98
7	0	110.21
7	0	110.25
7	0	110.29
7	0	110.36
7	0	110.65
7	0	110.71
7	0	110.74
7	0	110.85
7	0	110.9
7	0	110.9
7	0	110.97
7	0	111
7	0	111.03
7	0	111.17
7	0	111.27
7	0	111.34
7	0	111.42
7	0	111.47
7	0	111.48
7	0	111.48
7	0	111.67
7	0	111.68
7	0	111.71
7	0	111.72
7	0	111.78
7	0	111.81
7	0	111.87
7	0	111.99
7	0	112
7	0	112
7	0	112
7	0	112.24
7	0	112.37
7	0	112.63
7	0	112.83
7	0	112.83
7	0	112.88
7	0	112.92
7	0	112.96
7	0	112.99
7	0	113.01
7	0	113.12

7	0	113.38
7	0	113.65
7	0	113.78
7	0	113.83
7	0	117.41
7	0	117.43
7	0	117.58
7	0	117.6
7	0	117.61
7	0	117.64
7	0	117.79
7	0	117.79
7	0	117.82
7	0	117.83
7	0	117.87
7	0	117.9
7	0	117.93
7	0	118.07
7	0	118.2
7	0	118.35
7	0	118.37
7	0	118.57
7	0	118.57
7	0	118.6
7	0	118.72
7	0	118.76
7	0	118.87
7	0	119.02
7	0	119.21
7	0	119.42
7	0	119.46
7	0	119.49
7	0	119.49
7	0	119.58
7	0	119.9
7	0	119.95
7	0	120.05
7	0	120.24
7	0	120.38
7	0	120.53
7	0	120.59
7	0	120.69
7	0	120.69
7	0	120.71
7	0	120.74
7	0	120.82
7	0	120.83

7	0	120.95
7	0	121.15
7	0	121.75
7	0	121.79
7	0	122.21
7	0	122.21
7	0	122.45
7	0	122.45
7	0	122.65
7	0	122.75
7	0	122.83
7	0	122.87
7	0	122.87
7	0	122.87
7	0	122.93
7	0	123.15
7	0	123.21
7	0	123.47
7	0	123.5
7	0	123.58
7	0	123.6
7	0	123.62
7	0	123.73
7	0	123.86
7	0	124.05
7	0	124.2
7	0	124.26
7	0	124.35
7	0	124.69
7	0	124.83
7	0	124.85
7	0	124.85
7	0	124.96
7	0	125.09
7	0	125.35
7	0	125.35
7	0	125.47
7	0	125.61
7	0	125.87
7	0	125.95
7	0	126.12
7	0	126.27
7	0	126.55
7	0	126.74
7	0	126.89
7	0	127.12
7	0	127.2

7	0	127.26
7	0	127.26
7	0	127.51
7	0	127.68
7	0	128
7	0	129
7	0	129
7	0	129.49
7	0	130
7	0	130
7	0	130.16
7	0	130.26
7	0	130.4
7	0	131
7	0	131
7	0	131.1
7	0	132.13
7	0	132.79
7	0	134
7	0	134
7	0	134.91
7	0	135.84
7	0	136.66
7	0	136.96
7	0	137
7	0	137.04
7	0	137.06
7	0	137.6
7	0	137.69
7	0	138
7	0	138.13
7	0	138.35
7	0	138.4
7	0	139
7	0	139.62
7	0	139.9
7	0	140.1
7	0	140.24
7	0	140.41
7	0	140.68
7	0	140.72
7	0	141
7	0	141.32
7	0	141.44
7	0	142
7	0	142
7	0	142.31

7	0	143
7	0	143
7	0	145
7	0	147.74
7	0	150.38
7	0	151
7	0	151.22
7	0	152.93
7	0	153
7	0	153
7	0	153.41
7	1	77
7	1	78
7	1	78.73
7	1	79
7	1	79
7	1	79.45
7	1	79.84
7	1	79.95
7	1	80
7	1	80
7	1	80.05
7	1	81
7	1	81
7	1	81.5
7	1	83.25
7	1	83.93
7	1	84.28
7	1	84.45
7	1	84.71
7	1	85.08
7	1	85.11
7	1	85.13
7	1	85.29
7	1	85.45
7	1	88.9
7	1	89.35
7	1	89.42
7	1	89.65
7	1	89.82
7	1	89.91
7	1	90.76
7	1	91.24
7	1	91.28
7	1	91.28
7	1	91.48
7	1	91.57

7	1	91.63
7	1	91.65
7	1	91.83
7	1	92.01
7	1	92.23
7	1	92.24
7	1	92.56
7	1	92.74
7	1	92.83
7	1	93.19
7	1	93.33
7	1	94.06
7	1	94.21
7	1	94.21
7	1	94.27
7	1	95.14
7	1	95.38
7	1	95.54
7	1	95.95
7	1	95.96
7	1	95.97
7	1	96.29
7	1	96.48
7	1	96.55
7	1	96.8
7	1	96.85
7	1	96.9
7	1	97.3
7	1	97.51
7	1	97.58
7	1	97.63
7	1	97.77
7	1	97.78
7	1	97.88
7	1	98.36
7	1	98.47
7	1	98.58
7	1	98.67
7	1	98.81
7	1	98.84
7	1	99.21
7	1	99.24
7	1	99.98
7	1	100.4
7	1	100.4
7	1	100.56
7	1	100.82

7	1	100.83
7	1	101.24
7	1	101.36
7	1	101.39
7	1	101.42
7	1	101.63
7	1	101.69
7	1	102.1
7	1	102.21
7	1	102.22
7	1	102.42
7	1	102.47
7	1	102.62
7	1	103
7	1	103.04
7	1	103.27
7	1	103.33
7	1	103.34
7	1	103.36
7	1	103.41
7	1	103.46
7	1	103.67
7	1	103.81
7	1	103.88
7	1	103.94
7	1	104
7	1	104.06
7	1	104.16
7	1	104.19
7	1	104.34
7	1	104.59
7	1	104.72
7	1	104.76
7	1	104.82
7	1	104.86
7	1	104.87
7	1	104.92
7	1	104.93
7	1	105.92
7	1	105.95
7	1	106.04
7	1	106.06
7	1	106.08
7	1	106.09
7	1	106.17
7	1	106.24
7	1	106.38

7	1	106.44
7	1	106.53
7	1	106.55
7	1	106.56
7	1	106.58
7	1	106.6
7	1	106.81
7	1	106.84
7	1	106.97
7	1	107.07
7	1	107.09
7	1	107.18
7	1	107.37
7	1	107.37
7	1	107.39
7	1	107.4
7	1	107.73
7	1	107.77
7	1	107.94
7	1	107.98
7	1	108
7	1	108.07
7	1	108.08
7	1	108.11
7	1	108.33
7	1	108.34
7	1	108.44
7	1	108.48
7	1	108.81
7	1	109.01
7	1	109.06
7	1	109.12
7	1	109.21
7	1	109.22
7	1	109.22
7	1	109.23
7	1	109.38
7	1	109.41
7	1	109.69
7	1	109.76
7	1	109.84
7	1	110.03
7	1	110.06
7	1	110.07
7	1	110.09
7	1	110.09
7	1	110.18

7	1	110.34
7	1	110.43
7	1	110.43
7	1	110.53
7	1	110.56
7	1	110.6
7	1	110.61
7	1	110.65
7	1	110.66
7	1	110.69
7	1	110.71
7	1	110.94
7	1	110.97
7	1	111.07
7	1	111.26
7	1	111.28
7	1	111.3
7	1	111.31
7	1	111.5
7	1	111.68
7	1	111.9
7	1	111.94
7	1	111.97
7	1	112
7	1	112.01
7	1	112.14
7	1	112.42
7	1	112.43
7	1	112.52
7	1	112.53
7	1	112.62
7	1	112.63
7	1	112.7
7	1	112.73
7	1	112.74
7	1	112.82
7	1	112.82
7	1	112.86
7	1	113.1
7	1	113.18
7	1	113.21
7	1	113.27
7	1	113.3
7	1	113.35
7	1	113.45
7	1	113.52
7	1	113.53

7	1	113.54
7	1	113.61
7	1	113.67
7	1	113.7
7	1	113.7
7	1	113.76
7	1	113.78
7	1	113.83
7	1	113.91
7	1	113.92
7	1	113.93
7	1	114
7	1	114.01
7	1	114.03
7	1	114.13
7	1	114.13
7	1	114.19
7	1	114.27
7	1	114.41
7	1	114.47
7	1	114.49
7	1	114.5
7	1	114.54
7	1	114.6
7	1	114.63
7	1	114.65
7	1	114.66
7	1	114.72
7	1	114.77
7	1	114.79
7	1	114.86
7	1	114.97
7	1	114.97
7	1	115.03
7	1	115.08
7	1	115.09
7	1	115.15
7	1	115.2
7	1	115.21
7	1	115.21
7	1	115.22
7	1	115.22
7	1	115.23
7	1	115.31
7	1	115.35
7	1	115.45
7	1	115.46

7	1	115.53
7	1	115.54
7	1	115.55
7	1	115.57
7	1	115.58
7	1	115.6
7	1	115.6
7	1	115.6
7	1	115.64
7	1	115.64
7	1	115.64
7	1	115.74
7	1	115.81
7	1	115.89
7	1	115.89
7	1	115.89
7	1	115.92
7	1	115.93
7	1	116
7	1	116.02
7	1	116.03
7	1	116.11
7	1	116.12
7	1	116.14
7	1	116.17
7	1	116.2
7	1	116.22
7	1	116.25
7	1	116.26
7	1	116.27
7	1	116.28
7	1	116.31
7	1	116.31
7	1	116.36
7	1	116.41
7	1	116.42
7	1	116.45
7	1	116.45
7	1	116.52
7	1	116.56
7	1	116.59
7	1	116.59
7	1	116.61
7	1	116.62
7	1	116.71
7	1	116.73
7	1	116.73

7	1	116.74
7	1	116.76
7	1	116.79
7	1	116.82
7	1	116.85
7	1	116.92
7	1	116.92
7	1	116.93
7	1	116.93
7	1	116.93
7	1	116.96
7	1	116.98
7	1	117.01
7	1	117.04
7	1	117.1
7	1	117.14
7	1	117.14
7	1	117.14
7	1	117.21
7	1	117.29
7	1	117.31
7	1	117.33
7	1	117.35
7	1	117.38
7	1	117.39
7	1	117.39
7	1	117.4
7	1	117.4
7	1	117.4
7	1	117.46
7	1	117.49
7	1	117.55
7	1	117.56
7	1	117.61
7	1	117.62
7	1	117.66
7	1	117.89
7	1	117.97
7	1	118.03
7	1	118.04
7	1	118.11
7	1	118.11
7	1	118.14
7	1	118.21
7	1	118.36
7	1	118.41
7	1	118.5

7	1	118.56
7	1	118.7
7	1	118.79
7	1	118.82
7	1	118.97
7	1	119.05
7	1	119.13
7	1	119.14
7	1	119.18
7	1	119.19
7	1	119.34
7	1	119.38
7	1	119.56
7	1	119.66
7	1	119.9
7	1	119.92
7	1	119.95
7	1	120.07
7	1	120.19
7	1	120.35
7	1	120.36
7	1	120.36
7	1	120.59
7	1	120.73
7	1	120.86
7	1	120.98
7	1	121.01
7	1	121.04
7	1	121.28
7	1	121.34
7	1	121.38
7	1	121.47
7	1	121.48
7	1	121.61
7	1	121.68
7	1	121.76
7	1	121.95
7	1	121.95
7	1	121.98
7	1	122.06
7	1	122.29
7	1	122.4
7	1	122.54
7	1	122.76
7	1	122.77
7	1	122.87
7	1	123.13

7	1	123.25
7	1	123.38
7	1	123.43
7	1	123.71
7	1	123.73
7	1	123.88
7	1	124.19
7	1	124.25
7	1	124.31
7	1	124.45
7	1	124.6
7	1	124.62
7	1	124.78
7	1	125.04
7	1	125.09
7	1	125.2
7	1	125.46
7	1	125.5
7	1	125.55
7	1	125.7
7	1	125.72
7	1	125.78
7	1	125.92
7	1	126.04
7	1	126.37
7	1	126.38
7	1	126.43
7	1	126.43
7	1	126.52
7	1	126.58
7	1	126.81
7	1	126.94
7	1	126.96
7	1	126.97
7	1	127.06
7	1	127.23
7	1	127.26
7	1	127.3
7	1	127.35
7	1	127.47
7	1	127.61
7	1	127.67
7	1	127.74
7	1	127.81
7	1	127.9
7	1	127.97
7	1	128

7	1	128.25
7	1	128.37
7	1	128.46
7	1	128.51
7	1	128.59
7	1	128.84
7	1	128.88
7	1	129
7	1	129
7	1	129
7	1	129.04
7	1	129.07
7	1	129.43
7	1	129.49
7	1	129.49
7	1	129.56
7	1	129.81
7	1	129.91
7	1	130
7	1	130.03
7	1	130.15
7	1	130.69
7	1	131
7	1	131
7	1	131
7	1	131.1
7	1	131.35
7	1	131.94
7	1	132
7	1	133
7	1	133.32
7	1	134
7	1	134.44
7	1	134.65
7	1	134.71
7	1	134.76
7	1	134.82
7	1	135
7	1	135.06
7	1	135.13
7	1	135.26
7	1	136
7	1	137
7	1	137
7	1	162.91
8	0	87.61
8	0	88.11

8	0	88.3
8	0	88.36
8	0	88.47
8	0	88.98
8	0	89.43
8	0	89.73
8	0	89.97
8	0	90.18
8	0	90.19
8	0	91.17
8	0	91.29
8	0	91.32
8	0	92.74
8	0	92.91
8	0	93.1
8	0	93.6
8	0	93.69
8	0	93.74
8	0	93.94
8	0	94.18
8	0	94.47
8	0	94.54
8	0	94.58
8	0	94.6
8	0	94.71
8	0	94.82
8	0	94.93
8	0	95.12
8	0	95.45
8	0	95.52
8	0	95.78
8	0	95.91
8	0	96.25
8	0	96.28
8	0	96.9
8	0	97.18
8	0	97.37
8	0	97.37
8	0	97.42
8	0	97.43
8	0	97.45
8	0	97.48
8	0	97.52
8	0	97.68
8	0	97.69
8	0	97.82
8	0	97.86

8	0	97.87
8	0	98.04
8	0	98.08
8	0	98.11
8	0	98.23
8	0	98.44
8	0	98.45
8	0	98.47
8	0	98.58
8	0	99.07
8	0	99.18
8	0	99.27
8	0	99.34
8	0	99.41
8	0	99.47
8	0	99.59
8	0	99.82
8	0	99.93
8	0	99.93
8	0	100
8	0	100.06
8	0	100.09
8	0	100.15
8	0	100.22
8	0	100.28
8	0	100.33
8	0	100.5
8	0	100.54
8	0	100.62
8	0	100.69
8	0	101.01
8	0	101.2
8	0	101.22
8	0	101.24
8	0	101.53
8	0	101.58
8	0	101.62
8	0	101.67
8	0	101.73
8	0	101.88
8	0	101.97
8	0	101.97
8	0	102.03
8	0	102.07
8	0	102.14
8	0	102.17
8	0	102.25

8	0	102.27
8	0	102.27
8	0	102.34
8	0	102.41
8	0	102.56
8	0	102.89
8	0	102.98
8	0	103
8	0	103.09
8	0	103.22
8	0	103.24
8	0	103.28
8	0	103.36
8	0	103.37
8	0	103.38
8	0	103.43
8	0	103.46
8	0	103.56
8	0	103.65
8	0	103.69
8	0	103.82
8	0	103.88
8	0	103.9
8	0	103.9
8	0	103.91
8	0	103.91
8	0	103.93
8	0	103.94
8	0	104
8	0	104.05
8	0	104.1
8	0	104.12
8	0	104.22
8	0	104.24
8	0	104.3
8	0	104.31
8	0	104.31
8	0	104.58
8	0	104.61
8	0	104.62
8	0	104.64
8	0	104.65
8	0	104.95
8	0	105.05
8	0	105.07
8	0	105.1
8	0	105.16

8	0	105.16
8	0	105.16
8	0	105.31
8	0	105.34
8	0	105.52
8	0	105.55
8	0	105.63
8	0	105.64
8	0	105.65
8	0	105.66
8	0	105.67
8	0	105.67
8	0	105.71
8	0	105.75
8	0	105.87
8	0	105.87
8	0	105.87
8	0	105.91
8	0	106.12
8	0	106.12
8	0	106.64
8	0	106.65
8	0	106.65
8	0	106.78
8	0	106.79
8	0	106.81
8	0	106.93
8	0	106.97
8	0	106.97
8	0	106.98
8	0	107.09
8	0	107.14
8	0	107.17
8	0	107.27
8	0	107.29
8	0	107.34
8	0	107.39
8	0	107.43
8	0	107.5
8	0	107.52
8	0	107.6
8	0	107.67
8	0	107.71
8	0	107.71
8	0	107.75
8	0	107.81
8	0	107.84

8	0	107.84
8	0	107.93
8	0	107.96
8	0	107.97
8	0	108.02
8	0	108.08
8	0	108.08
8	0	108.09
8	0	108.1
8	0	108.12
8	0	108.17
8	0	108.25
8	0	108.29
8	0	108.32
8	0	108.34
8	0	108.39
8	0	108.44
8	0	108.6
8	0	108.68
8	0	108.69
8	0	108.71
8	0	108.77
8	0	108.85
8	0	108.9
8	0	108.9
8	0	108.91
8	0	108.92
8	0	108.99
8	0	109.04
8	0	109.22
8	0	109.29
8	0	109.3
8	0	109.35
8	0	109.44
8	0	109.47
8	0	109.48
8	0	109.5
8	0	109.51
8	0	109.52
8	0	109.67
8	0	109.73
8	0	109.79
8	0	109.81
8	0	109.9
8	0	109.94
8	0	110.02
8	0	110.03

8	0	110.06
8	0	110.09
8	0	110.09
8	0	110.15
8	0	110.19
8	0	110.21
8	0	110.34
8	0	110.37
8	0	110.4
8	0	110.41
8	0	110.43
8	0	110.5
8	0	110.52
8	0	110.56
8	0	110.58
8	0	110.67
8	0	110.71
8	0	110.72
8	0	110.72
8	0	110.73
8	0	110.73
8	0	110.76
8	0	110.76
8	0	110.79
8	0	110.79
8	0	110.86
8	0	110.87
8	0	110.9
8	0	110.93
8	0	110.97
8	0	111.05
8	0	111.07
8	0	111.07
8	0	111.09
8	0	111.12
8	0	111.16
8	0	111.16
8	0	111.16
8	0	111.17
8	0	111.18
8	0	111.19
8	0	111.2
8	0	111.23
8	0	111.26
8	0	111.29
8	0	111.31
8	0	111.34

8	0	111.39
8	0	111.41
8	0	111.49
8	0	111.76
8	0	111.87
8	0	111.92
8	0	111.96
8	0	111.97
8	0	112
8	0	112.02
8	0	112.29
8	0	112.37
8	0	112.38
8	0	112.39
8	0	112.43
8	0	112.46
8	0	112.47
8	0	112.55
8	0	112.56
8	0	112.65
8	0	112.71
8	0	112.72
8	0	112.8
8	0	112.81
8	0	112.82
8	0	112.82
8	0	112.83
8	0	112.85
8	0	112.86
8	0	113.05
8	0	113.08
8	0	113.1
8	0	113.26
8	0	113.29
8	0	113.35
8	0	113.37
8	0	113.38
8	0	113.48
8	0	113.61
8	0	113.61
8	0	113.62
8	0	113.63
8	0	113.67
8	0	113.73
8	0	113.85
8	0	114.01
8	0	114.02

8	0	114.15
8	0	114.16
8	0	117.51
8	0	117.56
8	0	117.65
8	0	117.68
8	0	117.78
8	0	117.8
8	0	118
8	0	118.06
8	0	118.07
8	0	118.07
8	0	118.15
8	0	118.15
8	0	118.16
8	0	118.21
8	0	118.25
8	0	118.38
8	0	118.46
8	0	118.46
8	0	118.47
8	0	118.48
8	0	118.55
8	0	118.59
8	0	118.6
8	0	118.64
8	0	118.73
8	0	118.79
8	0	118.86
8	0	119.02
8	0	120.75
8	0	120.8
8	0	120.82
8	0	120.83
8	0	120.99
8	0	121.02
8	0	121.11
8	0	121.26
8	0	121.3
8	0	121.33
8	0	121.34
8	0	121.38
8	0	121.38
8	0	121.49
8	0	121.51
8	0	121.51
8	0	121.61

8	0	121.64
8	0	121.73
8	0	121.79
8	0	121.79
8	0	121.9
8	0	121.91
8	0	122.2
8	0	122.22
8	0	122.34
8	0	122.38
8	0	122.38
8	0	122.45
8	0	122.46
8	0	122.61
8	0	122.72
8	0	122.73
8	0	122.78
8	0	122.83
8	0	122.89
8	0	123.02
8	0	123.05
8	0	123.08
8	0	123.16
8	0	123.32
8	0	123.32
8	0	123.36
8	0	123.38
8	0	123.46
8	0	123.51
8	0	123.54
8	0	123.61
8	0	123.74
8	0	123.9
8	0	123.94
8	0	124
8	0	124.03
8	0	124.11
8	0	124.21
8	0	124.31
8	0	124.35
8	0	125.07
8	0	125.11
8	0	125.24
8	0	125.28
8	0	125.38
8	0	125.38
8	0	125.39

8	0	125.4
8	0	125.4
8	0	125.42
8	0	125.47
8	0	125.53
8	0	125.58
8	0	125.7
8	0	125.72
8	0	125.96
8	0	126.19
8	0	126.43
8	0	126.43
8	0	126.5
8	0	126.7
8	0	126.89
8	0	126.97
8	0	127.41
8	0	127.45
8	0	127.46
8	0	127.59
8	0	127.6
8	0	127.7
8	0	127.96
8	0	128
8	0	128
8	0	128.03
8	0	128.04
8	0	128.29
8	0	128.47
8	0	128.56
8	0	128.87
8	0	128.88
8	0	128.9
8	0	128.99
8	0	129
8	0	129
8	0	129.15
8	0	129.34
8	0	129.59
8	0	129.78
8	0	129.9
8	0	129.91
8	0	129.94
8	0	130.03
8	0	130.03
8	0	130.18
8	0	130.5

8	0	130.63
8	0	130.76
8	0	130.91
8	0	131.12
8	0	131.15
8	0	131.34
8	0	131.41
8	0	131.49
8	0	132
8	0	132
8	0	132
8	0	132.12
8	0	132.32
8	0	132.62
8	0	132.91
8	0	133
8	0	133
8	0	133.22
8	0	133.31
8	0	133.37
8	0	133.66
8	0	133.82
8	0	134.09
8	0	134.31
8	0	134.44
8	0	134.76
8	0	134.91
8	0	135.35
8	0	135.65
8	0	135.87
8	0	136
8	0	136
8	0	136
8	0	136.07
8	0	136.25
8	0	136.41
8	0	136.49
8	0	136.59
8	0	136.62
8	0	136.74
8	0	137
8	0	137
8	0	137.29
8	0	137.82
8	0	138
8	0	138
8	0	138.07

8	0	138.19
8	0	138.29
8	0	138.34
8	0	138.5
8	0	138.74
8	0	138.94
8	0	139.31
8	0	139.34
8	0	139.46
8	0	139.62
8	0	140
8	0	140
8	0	140
8	0	140
8	0	140
8	0	140.04
8	0	140.28
8	0	140.41
8	0	140.51
8	0	140.99
8	0	141.01
8	0	141.37
8	0	141.65
8	0	142.07
8	0	142.79
8	0	143
8	0	143
8	0	143
8	0	143.56
8	0	144.21
8	0	144.44
8	0	144.44
8	0	145.16
8	0	145.46
8	0	145.71
8	0	145.82
8	0	146.51
8	0	146.87
8	0	147
8	0	147.09
8	0	147.13
8	0	147.46
8	0	148
8	0	148
8	0	148.63
8	0	149.01
8	0	149.9

8	0	149.97
8	0	150
8	0	150
8	0	152.6
8	0	153
8	1	119.52
8	1	119.68
8	1	119.75
8	1	119.79
8	1	120.1
8	1	120.34
8	1	120.45
8	1	120.5
8	1	120.53
8	1	120.55
8	1	120.66
8	1	90.91
8	1	94.34
8	1	95.34
8	1	95.68
8	1	96.51
8	1	96.61
8	1	96.84
8	1	97.24
8	1	97.75
8	1	98.69
8	1	98.85
8	1	100.08
8	1	100.51
8	1	101.46
8	1	102.72
8	1	103.47
8	1	103.93
8	1	104.21
8	1	104.5
8	1	105.93
8	1	105.95
8	1	105.98
8	1	106.1
8	1	106.1
8	1	106.16
8	1	106.21
8	1	106.28
8	1	106.32
8	1	106.36
8	1	106.36
8	1	106.45

8	1	106.54
8	1	106.81
8	1	106.85
8	1	106.92
8	1	107.05
8	1	107.37
8	1	108.19
8	1	108.65
8	1	108.84
8	1	109.26
8	1	109.87
8	1	110.8
8	1	111.03
8	1	111.24
8	1	111.71
8	1	111.91
8	1	112.02
8	1	112.53
8	1	113.3
8	1	113.91
8	1	113.92
8	1	113.93
8	1	114
8	1	114.05
8	1	114.09
8	1	114.12
8	1	114.19
8	1	114.2
8	1	114.27
8	1	114.3
8	1	114.34
8	1	114.34
8	1	114.34
8	1	114.38
8	1	114.41
8	1	114.43
8	1	114.51
8	1	114.57
8	1	114.7
8	1	114.72
8	1	114.75
8	1	114.77
8	1	114.79
8	1	114.83
8	1	114.84
8	1	114.86
8	1	114.89

8	1	114.9
8	1	114.96
8	1	115.17
8	1	115.2
8	1	115.49
8	1	115.52
8	1	115.59
8	1	115.6
8	1	115.6
8	1	115.72
8	1	115.75
8	1	115.75
8	1	115.82
8	1	115.83
8	1	115.88
8	1	115.88
8	1	115.91
8	1	115.99
8	1	116.12
8	1	116.14
8	1	116.18
8	1	116.23
8	1	116.26
8	1	116.63
8	1	116.64
8	1	116.7
8	1	116.73
8	1	116.73
8	1	116.77
8	1	116.8
8	1	116.81
8	1	116.85
8	1	116.85
8	1	116.94
8	1	116.98
8	1	117.06
8	1	117.12
8	1	117.18
8	1	117.2
8	1	117.3
8	1	117.34
8	1	117.36
8	1	117.38
8	1	117.41
8	1	118.1
8	1	118.13
8	1	118.68

8	1	118.7
8	1	118.94
8	1	118.99
8	1	119.05
8	1	119.31
8	1	119.37
8	1	119.48
8	1	119.67
8	1	119.68
8	1	120.4
8	1	120.42
8	1	120.83
8	1	120.87
8	1	120.93
8	1	121.21
8	1	121.42
8	1	121.45
8	1	121.56
8	1	121.58
8	1	121.85
8	1	122.12
8	1	122.91
8	1	123
8	1	123.05
8	1	123.16
8	1	123.26
8	1	123.34
8	1	123.45
8	1	123.63
8	1	123.68
8	1	123.82
8	1	124.01
8	1	124.07
8	1	124.6
8	1	124.68
8	1	124.73
8	1	124.76
8	1	125.03
8	1	125.26
8	1	125.36
8	1	125.66
8	1	125.77
8	1	125.8
8	1	126.22
8	1	126.25
8	1	126.47
8	1	126.58

8	1	126.77
8	1	126.85
8	1	126.87
8	1	126.94
8	1	127.15
8	1	127.53
8	1	127.59
8	1	127.62
8	1	127.84
8	1	128
8	1	128.4
8	1	128.59
8	1	128.96
8	1	129
8	1	129.07
8	1	129.87
8	1	129.97
8	1	130
8	1	131
8	1	131
8	1	131
8	1	131.88
8	1	132
8	1	132.29
8	1	132.29
8	1	132.46
8	1	132.57
8	1	132.57
8	1	133
8	1	133
8	1	133
8	1	133.5
8	1	133.53
8	1	133.57
8	1	134
8	1	134.32
8	1	134.51
8	1	134.57
8	1	134.82
8	1	134.88
8	1	135
8	1	135
8	1	135.93
8	1	136
8	1	161.44
9	0	76
9	0	92.65

9	0	95.96
9	0	97.13
9	0	97.51
9	0	98.64
9	0	98.72
9	0	99.49
9	0	100.59
9	0	102.03
9	0	102.89
9	0	104.26
9	0	105.16
9	0	105.56
9	0	105.64
9	0	105.75
9	0	107.37
9	0	107.73
9	0	108.14
9	0	108.46
9	0	109.75
9	0	110.87
9	0	112.27
9	0	113.05
9	0	113.28
9	0	118.12
9	0	118.15
9	0	119.17
9	0	119.46
9	0	120.72
9	0	120.9
9	0	121.45
9	0	121.68
9	0	122.14
9	0	122.73
9	0	122.96
9	0	123.57
9	0	125.29
9	0	125.57
9	0	125.97
9	0	126.18
9	0	126.3
9	0	126.47
9	0	126.66
9	0	126.73
9	0	127.06
9	0	127.28
9	0	127.42
9	0	127.71

9	0	129.99
9	0	130.06
9	0	131.82
9	0	132.03
9	0	132.15
9	0	132.16
9	0	132.63
9	0	132.66
9	0	132.74
9	0	133
9	0	133.35
9	0	135
9	0	135.25
9	0	135.49
9	0	135.66
9	0	136.04
9	0	137.12
9	0	138
9	0	138
9	0	138.03
9	0	139.16
9	0	139.4
9	0	140.12
9	0	140.72
9	0	141
9	0	141.07
9	0	142.9
9	0	143.93
9	0	144.9
9	0	149.85
9	0	150
9	0	155
9	1	89.51
9	1	89.53
9	1	95.13
9	1	95.36
9	1	95.95
9	1	96.25
9	1	96.38
9	1	97.14
9	1	97.98
9	1	98.4
9	1	98.87
9	1	99.81
9	1	100.79
9	1	100.81
9	1	102.1

9	1	102.52
9	1	102.74
9	1	102.77
9	1	103.25
9	1	103.83
9	1	103.92
9	1	104.09
9	1	104.44
9	1	104.58
9	1	107.22
9	1	107.97
9	1	108.68
9	1	108.94
9	1	109.24
9	1	109.9
9	1	110.01
9	1	110.25
9	1	110.26
9	1	111.04
9	1	111.41
9	1	111.48
9	1	111.65
9	1	112.01
9	1	113.46
9	1	114.19
9	1	114.69
9	1	114.8
9	1	114.81
9	1	115.2
9	1	115.25
9	1	115.31
9	1	115.34
9	1	115.76
9	1	116.01
9	1	116.08
9	1	116.32
9	1	116.54
9	1	116.57
9	1	116.78
9	1	116.89
9	1	116.93
9	1	116.97
9	1	117.07
9	1	117.65
9	1	118.08
9	1	118.14
9	1	118.37

9	1	118.72
9	1	118.78
9	1	119.13
9	1	119.89
9	1	120.24
9	1	120.36
9	1	120.46
9	1	120.69
9	1	120.78
9	1	121.01
9	1	121.28
9	1	121.34
9	1	121.76
9	1	123.99
9	1	123.99
9	1	124.24
9	1	125.34
9	1	125.77
9	1	126.64
9	1	126.88
9	1	127.37
9	1	127.54
9	1	128
9	1	128.49
9	1	128.74
9	1	129
9	1	129
9	1	130
9	1	130.51
9	1	132
9	1	134.32
9	1	135.28
9	1	137
9	1	137
10	0	79.06
10	0	81.08
10	0	81.73
10	0	82.03
10	0	83.06
10	0	84.16
10	0	86.07
10	0	86.95
10	0	87.39
10	0	87.47
10	0	87.58
10	0	88.09
10	0	88.14

10	0	88.53
10	0	88.62
10	0	89.32
10	0	89.34
10	0	89.63
10	0	89.84
10	0	90.94
10	0	90.95
10	0	91.01
10	0	91.02
10	0	91.9
10	0	92.02
10	0	92.49
10	0	92.56
10	0	92.71
10	0	92.73
10	0	92.91
10	0	93.01
10	0	93.1
10	0	93.12
10	0	93.35
10	0	93.4
10	0	93.41
10	0	93.54
10	0	93.68
10	0	94.27
10	0	94.53
10	0	94.83
10	0	95.09
10	0	95.37
10	0	95.38
10	0	95.55
10	0	95.66
10	0	95.77
10	0	95.78
10	0	95.8
10	0	96.27
10	0	96.45
10	0	96.55
10	0	96.55
10	0	97.17
10	0	97.24
10	0	97.83
10	0	97.89
10	0	98.06
10	0	98.57
10	0	98.68

10	0	98.86
10	0	98.91
10	0	99.16
10	0	99.34
10	0	99.59
10	0	99.85
10	0	100.08
10	0	100.12
10	0	100.26
10	0	100.64
10	0	100.86
10	0	100.93
10	0	101.1
10	0	101.19
10	0	101.46
10	0	101.51
10	0	101.79
10	0	101.81
10	0	101.98
10	0	102.11
10	0	102.26
10	0	102.55
10	0	103.08
10	0	103.36
10	0	103.37
10	0	103.45
10	0	103.66
10	0	103.66
10	0	103.71
10	0	103.75
10	0	103.83
10	0	103.88
10	0	103.97
10	0	104.14
10	0	104.15
10	0	104.17
10	0	104.18
10	0	104.29
10	0	104.34
10	0	104.37
10	0	104.48
10	0	104.55
10	0	105.09
10	0	105.1
10	0	105.24
10	0	105.24
10	0	105.29

10	0	105.33
10	0	105.35
10	0	105.35
10	0	105.43
10	0	105.45
10	0	105.53
10	0	105.66
10	0	105.72
10	0	105.74
10	0	105.87
10	0	106.26
10	0	106.59
10	0	107.71
10	0	107.85
10	0	107.86
10	0	108.12
10	0	108.15
10	0	108.18
10	0	108.2
10	0	108.25
10	0	108.26
10	0	108.54
10	0	108.78
10	0	108.95
10	0	109.1
10	0	109.13
10	0	109.63
10	0	109.76
10	0	109.83
10	0	109.95
10	0	110.01
10	0	110.1
10	0	110.19
10	0	110.3
10	0	110.36
10	0	110.41
10	0	110.56
10	0	110.93
10	0	111.02
10	0	111.15
10	0	111.19
10	0	111.27
10	0	111.42
10	0	112.1
10	0	112.7
10	0	112.83
10	0	113.29

10	0	113.33
10	0	113.51
10	0	113.64
10	0	114.03
10	0	117.76
10	0	117.78
10	0	117.81
10	0	117.93
10	0	118.32
10	0	118.36
10	0	118.6
10	0	118.81
10	0	119.09
10	0	119.8
10	0	119.89
10	0	120.21
10	0	120.68
10	0	120.7
10	0	120.83
10	0	121.21
10	0	121.26
10	0	121.48
10	0	122.29
10	0	123.34
10	0	123.45
10	0	123.78
10	0	123.86
10	0	124.3
10	0	124.66
10	0	124.84
10	0	125.08
10	0	125.3
10	0	125.44
10	0	125.77
10	0	126.18
10	0	127.46
10	0	128
10	0	128.46
10	0	130.26
10	0	130.66
10	0	149.53
10	1	83.78
10	1	84.42
10	1	88.83
10	1	89.09
10	1	90.61
10	1	90.66

10	1	91.07
10	1	91.66
10	1	91.95
10	1	92
10	1	93.41
10	1	93.95
10	1	96.06
10	1	96.63
10	1	97.42
10	1	97.48
10	1	97.54
10	1	98.02
10	1	98.81
10	1	98.84
10	1	99.24
10	1	99.91
10	1	100.22
10	1	100.66
10	1	100.7
10	1	101.12
10	1	101.21
10	1	101.73
10	1	102.16
10	1	102.73
10	1	103.02
10	1	103.13
10	1	103.45
10	1	103.46
10	1	104
10	1	104.3
10	1	104.55
10	1	104.85
10	1	105.99
10	1	106.03
10	1	106.04
10	1	106.06
10	1	106.1
10	1	106.11
10	1	106.24
10	1	106.3
10	1	106.35
10	1	106.53
10	1	106.57
10	1	106.58
10	1	106.83
10	1	106.92
10	1	107.35

10	1	107.7
10	1	107.76
10	1	107.88
10	1	109.19
10	1	110.34
10	1	112.4
10	1	113.94
10	1	113.99
10	1	114.21
10	1	114.72
10	1	114.81
10	1	114.93
10	1	114.97
10	1	115.06
10	1	115.09
10	1	115.18
10	1	115.83
10	1	115.87
10	1	116.75
10	1	117.14
10	1	117.23
10	1	117.25
10	1	117.28
10	1	117.4
10	1	117.49
10	1	118.98
10	1	119.98
10	1	128.75
10	1	135.88
10	1	137.09
11	0	73.45
11	0	75
11	0	82.67
11	0	82.9
11	0	87.46
11	0	93.68
11	0	94.49
11	0	95.58
11	0	96.38
11	0	97.13
11	0	98.18
11	0	98.3
11	0	98.49
11	0	99.75
11	0	99.82
11	0	101.08
11	0	101.09

11	0	102.17
11	0	104.82
11	0	105.07
11	0	105.17
11	0	105.34
11	0	105.59
11	0	105.7
11	0	105.82
11	0	106.86
11	0	108.29
11	0	108.34
11	0	108.69
11	0	111.53
11	0	112.6
11	0	113.18
11	0	113.56
11	0	113.72
11	0	118.19
11	0	118.87
11	0	119.7
11	0	119.9
11	0	121.3
11	0	121.33
11	0	121.45
11	0	121.91
11	0	122.42
11	0	122.78
11	0	123.25
11	0	123.61
11	0	124.11
11	0	124.21
11	0	124.4
11	0	124.9
11	0	125.59
11	0	125.64
11	0	125.68
11	0	125.86
11	0	126.12
11	0	126.68
11	0	126.81
11	0	127.35
11	0	127.73
11	0	128.38
11	0	128.63
11	0	128.99
11	0	129
11	0	129

11	0	130
11	0	131
11	0	131.31
11	0	131.76
11	0	132
11	0	132
11	0	132.87
11	0	132.87
11	0	132.91
11	0	133
11	0	133
11	0	133.34
11	0	133.49
11	0	133.59
11	0	133.62
11	0	133.82
11	0	134
11	0	134
11	0	134.12
11	0	134.21
11	0	134.74
11	0	135.03
11	0	135.5
11	0	135.81
11	0	136
11	0	136
11	0	136
11	0	136.13
11	0	137
11	0	137
11	0	137.74
11	0	137.79
11	0	137.82
11	0	138
11	0	138
11	0	139.35
11	0	140.04
11	0	140.31
11	0	140.43
11	0	141
11	0	142
11	0	142
11	0	143.19
11	0	144
11	0	145.07
11	0	146
11	0	151.32

11	0	152.01
11	0	155.5
11	1	78.23
11	1	79.2
11	1	80
11	1	83.63
11	1	84.27
11	1	85.76
11	1	89.05
11	1	89.54
11	1	89.64
11	1	89.66
11	1	89.88
11	1	89.9
11	1	90.88
11	1	91.14
11	1	92.36
11	1	92.58
11	1	92.71
11	1	92.72
11	1	93.32
11	1	93.45
11	1	93.53
11	1	93.72
11	1	93.95
11	1	94.53
11	1	94.56
11	1	95.13
11	1	95.33
11	1	95.56
11	1	95.85
11	1	96.22
11	1	96.4
11	1	96.88
11	1	96.96
11	1	97.08
11	1	97.15
11	1	97.35
11	1	97.48
11	1	97.55
11	1	97.6
11	1	97.66
11	1	97.73
11	1	97.9
11	1	98.12
11	1	98.15
11	1	98.69

11	1	99.39
11	1	99.66
11	1	99.74
11	1	99.83
11	1	99.97
11	1	100.25
11	1	100.3
11	1	100.42
11	1	100.65
11	1	100.8
11	1	100.95
11	1	101.08
11	1	101.09
11	1	101.12
11	1	101.35
11	1	101.98
11	1	102.15
11	1	102.48
11	1	102.75
11	1	102.94
11	1	102.97
11	1	103.08
11	1	103.25
11	1	103.44
11	1	103.49
11	1	103.78
11	1	104.45
11	1	104.5
11	1	104.84
11	1	104.86
11	1	105.91
11	1	106.09
11	1	106.15
11	1	106.2
11	1	106.34
11	1	106.51
11	1	106.53
11	1	106.65
11	1	106.85
11	1	106.88
11	1	107.01
11	1	107.06
11	1	107.27
11	1	107.27
11	1	107.33
11	1	107.53
11	1	107.72

11	1	107.89
11	1	108.14
11	1	108.15
11	1	108.47
11	1	108.51
11	1	108.55
11	1	108.81
11	1	108.89
11	1	109.08
11	1	109.27
11	1	109.28
11	1	109.48
11	1	110
11	1	110.06
11	1	110.1
11	1	110.25
11	1	110.61
11	1	110.63
11	1	110.74
11	1	110.81
11	1	110.86
11	1	111.09
11	1	111.22
11	1	111.24
11	1	111.33
11	1	111.48
11	1	111.62
11	1	111.78
11	1	112
11	1	112.1
11	1	112.15
11	1	112.24
11	1	112.47
11	1	112.62
11	1	112.64
11	1	112.75
11	1	112.88
11	1	113.04
11	1	113.15
11	1	113.15
11	1	113.15
11	1	113.17
11	1	113.35
11	1	114.05
11	1	114.14
11	1	114.27
11	1	114.32

11	1	114.4
11	1	114.42
11	1	114.44
11	1	114.64
11	1	114.69
11	1	114.75
11	1	114.95
11	1	114.98
11	1	115.25
11	1	115.3
11	1	115.35
11	1	115.41
11	1	115.45
11	1	115.49
11	1	115.5
11	1	115.53
11	1	115.6
11	1	115.62
11	1	115.95
11	1	115.97
11	1	115.98
11	1	116.02
11	1	116.03
11	1	116.04
11	1	116.14
11	1	116.2
11	1	116.26
11	1	116.32
11	1	116.38
11	1	116.43
11	1	116.48
11	1	116.57
11	1	116.65
11	1	116.91
11	1	116.93
11	1	116.94
11	1	116.98
11	1	117
11	1	117.03
11	1	117.29
11	1	117.35
11	1	117.37
11	1	117.58
11	1	117.72
11	1	117.73
11	1	117.74
11	1	117.74

11	1	117.8
11	1	117.89
11	1	118.14
11	1	118.19
11	1	118.33
11	1	118.41
11	1	118.53
11	1	118.75
11	1	118.75
11	1	118.84
11	1	118.88
11	1	118.94
11	1	119
11	1	119.01
11	1	119.14
11	1	119.2
11	1	119.4
11	1	119.4
11	1	119.51
11	1	119.58
11	1	119.61
11	1	119.63
11	1	119.75
11	1	119.76
11	1	119.81
11	1	119.82
11	1	119.84
11	1	120.31
11	1	120.64
11	1	120.85
11	1	120.91
11	1	121.03
11	1	121.21
11	1	121.33
11	1	121.49
11	1	121.58
11	1	121.61
11	1	121.7
11	1	121.75
11	1	121.77
11	1	121.8
11	1	121.83
11	1	121.86
11	1	122.12
11	1	122.14
11	1	122.14
11	1	122.18

11	1	122.47
11	1	122.63
11	1	122.72
11	1	122.84
11	1	122.93
11	1	122.97
11	1	123.18
11	1	123.22
11	1	123.24
11	1	123.3
11	1	123.44
11	1	123.71
11	1	123.74
11	1	123.84
11	1	123.86
11	1	124.16
11	1	124.16
11	1	124.58
11	1	124.66
11	1	124.75
11	1	124.76
11	1	124.85
11	1	124.9
11	1	124.94
11	1	125.1
11	1	125.14
11	1	125.15
11	1	125.15
11	1	125.39
11	1	125.54
11	1	125.58
11	1	125.61
11	1	125.63
11	1	125.77
11	1	125.89
11	1	126
11	1	126.18
11	1	126.27
11	1	126.34
11	1	126.42
11	1	126.47
11	1	126.5
11	1	126.55
11	1	126.86
11	1	126.88
11	1	127.09
11	1	127.14

11	1	127.43
11	1	127.43
11	1	127.76
11	1	127.99
11	1	128
11	1	128
11	1	128.78
11	1	129
11	1	129.12
11	1	129.72
11	1	130.5
11	1	131.76
11	1	131.81
11	1	131.97
11	1	132
11	1	133
11	1	133
11	1	133.66
11	1	133.81
11	1	135
11	1	135
11	1	135.65
11	1	135.65
11	1	137.43
11	1	137.57
12	0	82.2
12	0	87.29
12	0	89.78
12	0	90.45
12	0	91.3
12	0	94.98
12	0	98.6
12	0	99.15
12	0	100.03
12	0	100.1
12	0	100.59
12	0	102.78
12	0	103.67
12	0	103.86
12	0	104.52
12	0	104.64
12	0	105.3
12	0	105.5
12	0	105.71
12	0	105.76
12	0	105.88
12	0	106.74

12	0	107.28
12	0	107.35
12	0	108.53
12	0	109.29
12	0	109.6
12	0	109.97
12	0	111.46
12	0	111.83
12	0	111.98
12	0	112.57
12	0	112.58
12	0	113.19
12	0	113.75
12	0	118.47
12	0	118.76
12	0	119.43
12	0	120.14
12	0	120.74
12	0	121.26
12	0	121.67
12	0	121.7
12	0	122.64
12	0	124.95
12	0	125.83
12	0	126.64
12	0	127.57
12	0	128
12	0	129.91
12	0	130
12	0	130.94
12	0	131.09
12	0	131.29
12	0	132.93
12	0	133
12	0	134.9
12	0	135
12	0	136
12	0	136
12	0	137.06
12	0	138.6
12	0	138.62
12	0	138.65
12	0	138.79
12	0	139
12	0	139.78
12	0	140.13
12	0	141.25

12	0	143
12	0	144
12	0	144.84
12	0	146.44
12	0	148.66
12	0	150
12	1	88.63
12	1	91.03
12	1	93.03
12	1	93.13
12	1	94.75
12	1	95.63
12	1	95.72
12	1	97.33
12	1	97.37
12	1	98.41
12	1	99.06
12	1	99.92
12	1	100.02
12	1	100.17
12	1	100.6
12	1	100.65
12	1	101.07
12	1	101.36
12	1	101.5
12	1	101.8
12	1	103.04
12	1	103.15
12	1	103.49
12	1	103.55
12	1	103.57
12	1	103.58
12	1	103.61
12	1	103.63
12	1	103.69
12	1	103.83
12	1	103.96
12	1	104.1
12	1	104.56
12	1	104.7
12	1	104.77
12	1	104.87
12	1	104.87
12	1	104.9
12	1	105.98
12	1	106.16
12	1	106.4

12	1	106.41
12	1	106.8
12	1	106.86
12	1	106.88
12	1	107.09
12	1	107.18
12	1	107.19
12	1	107.54
12	1	107.74
12	1	108.29
12	1	108.41
12	1	108.53
12	1	108.64
12	1	108.68
12	1	108.8
12	1	109.19
12	1	109.21
12	1	109.33
12	1	109.54
12	1	109.59
12	1	109.76
12	1	109.79
12	1	109.87
12	1	110.14
12	1	110.31
12	1	110.34
12	1	110.34
12	1	111.08
12	1	111.25
12	1	111.3
12	1	111.31
12	1	111.32
12	1	111.35
12	1	111.47
12	1	111.67
12	1	111.74
12	1	111.78
12	1	111.8
12	1	111.95
12	1	112.16
12	1	112.18
12	1	112.2
12	1	112.21
12	1	112.29
12	1	112.5
12	1	112.5
12	1	112.57

12	1	112.89
12	1	112.97
12	1	112.98
12	1	113.05
12	1	113.11
12	1	113.19
12	1	113.27
12	1	113.33
12	1	113.36
12	1	113.62
12	1	113.71
12	1	113.83
12	1	114.01
12	1	114.1
12	1	114.32
12	1	114.42
12	1	114.5
12	1	114.65
12	1	114.66
12	1	114.67
12	1	114.85
12	1	114.95
12	1	115.15
12	1	115.18
12	1	115.36
12	1	115.6
12	1	115.62
12	1	115.64
12	1	115.66
12	1	115.7
12	1	115.74
12	1	115.95
12	1	116.04
12	1	116.09
12	1	116.13
12	1	116.13
12	1	116.23
12	1	116.38
12	1	116.42
12	1	116.45
12	1	116.52
12	1	116.63
12	1	116.71
12	1	116.72
12	1	116.77
12	1	116.79
12	1	116.84

12	1	117.02
12	1	117.19
12	1	117.26
12	1	117.68
12	1	117.69
12	1	117.76
12	1	117.81
12	1	117.83
12	1	118.07
12	1	118.13
12	1	118.14
12	1	118.15
12	1	118.2
12	1	118.21
12	1	118.22
12	1	118.36
12	1	118.37
12	1	118.39
12	1	118.43
12	1	118.47
12	1	118.55
12	1	118.55
12	1	118.57
12	1	118.67
12	1	118.75
12	1	118.81
12	1	118.83
12	1	118.87
12	1	118.88
12	1	119.06
12	1	119.14
12	1	119.41
12	1	119.42
12	1	119.47
12	1	119.5
12	1	119.61
12	1	119.64
12	1	119.64
12	1	119.69
12	1	119.84
12	1	119.87
12	1	119.94
12	1	120.02
12	1	120.06
12	1	120.11
12	1	120.19
12	1	120.36

12	1	120.38
12	1	120.54
12	1	120.6
12	1	120.62
12	1	120.7
12	1	120.75
12	1	120.99
12	1	121.03
12	1	121.19
12	1	121.22
12	1	121.24
12	1	121.31
12	1	121.32
12	1	121.34
12	1	121.48
12	1	121.5
12	1	121.53
12	1	121.68
12	1	121.69
12	1	121.7
12	1	121.97
12	1	122.12
12	1	122.19
12	1	122.21
12	1	122.34
12	1	122.36
12	1	122.45
12	1	122.63
12	1	122.86
12	1	123.05
12	1	123.07
12	1	123.09
12	1	123.28
12	1	123.3
12	1	123.3
12	1	123.44
12	1	123.48
12	1	123.56
12	1	123.71
12	1	123.81
12	1	123.85
12	1	123.9
12	1	124.07
12	1	124.19
12	1	124.25
12	1	124.29
12	1	124.44

12	1	124.58
12	1	124.63
12	1	124.74
12	1	124.78
12	1	124.82
12	1	124.88
12	1	125.1
12	1	125.69
12	1	125.69
12	1	126.07
12	1	126.08
12	1	126.13
12	1	126.16
12	1	126.28
12	1	126.43
12	1	126.45
12	1	126.51
12	1	126.58
12	1	126.76
12	1	126.82
12	1	126.9
12	1	127
12	1	127.07
12	1	127.28
12	1	127.29
12	1	127.43
12	1	127.48
12	1	127.55
12	1	127.81
12	1	127.91
12	1	127.94
12	1	128
12	1	128
12	1	128
12	1	128
12	1	128
12	1	128.21
12	1	128.24
12	1	128.32
12	1	128.51
12	1	128.56
12	1	128.91
12	1	129
12	1	129
12	1	129.07
12	1	129.12
12	1	129.32

12	1	129.65
12	1	130
12	1	130
12	1	130
12	1	130.38
12	1	131
12	1	131.03
12	1	131.85
12	1	132
12	1	132
12	1	132.43
12	1	132.99
12	1	133.28
12	1	133.62
12	1	134.72
12	1	135
12	1	135.09
12	1	135.63
12	1	135.85
12	1	136
12	1	136
12	1	136
12	1	136.32
12	1	137
12	1	137
12	1	137.51
13	0	84.14
13	0	85.73
13	0	90.97
13	0	91.47
13	0	92.47
13	0	92.59
13	0	94.23
13	0	94.31
13	0	95.05
13	0	95.84
13	0	96.23
13	0	96.77
13	0	98.11
13	0	98.12
13	0	98.59
13	0	98.7
13	0	99.03
13	0	99.27
13	0	99.3
13	0	100.19
13	0	100.38

13	0	100.68
13	0	101.06
13	0	101.2
13	0	101.71
13	0	101.95
13	0	102.1
13	0	102.21
13	0	102.25
13	0	102.62
13	0	103.94
13	0	104.38
13	0	104.47
13	0	104.5
13	0	104.58
13	0	104.61
13	0	105.19
13	0	105.4
13	0	105.41
13	0	105.53
13	0	106.2
13	0	106.87
13	0	107.04
13	0	107.22
13	0	107.37
13	0	107.65
13	0	108.15
13	0	108.25
13	0	108.32
13	0	108.47
13	0	108.59
13	0	108.6
13	0	108.81
13	0	108.91
13	0	108.91
13	0	109.13
13	0	109.34
13	0	109.57
13	0	109.6
13	0	109.77
13	0	109.85
13	0	109.87
13	0	110.39
13	0	110.49
13	0	110.5
13	0	110.55
13	0	110.7
13	0	110.72

13	0	110.92
13	0	110.97
13	0	111.45
13	0	112.13
13	0	112.39
13	0	112.42
13	0	112.46
13	0	113.45
13	0	117.71
13	0	118.35
13	0	118.43
13	0	118.8
13	0	118.85
13	0	118.86
13	0	118.89
13	0	119
13	0	119.46
13	0	119.51
13	0	119.78
13	0	119.9
13	0	120.42
13	0	121.39
13	0	121.43
13	0	121.5
13	0	122.17
13	0	122.32
13	0	122.49
13	0	122.97
13	0	123.04
13	0	123.31
13	0	123.61
13	0	123.77
13	0	124.02
13	0	124.43
13	0	124.5
13	0	124.83
13	0	126.3
13	0	127.44
13	0	127.49
13	0	127.54
13	0	127.61
13	0	127.91
13	0	128.1
13	0	128.15
13	0	129
13	0	129.06
13	0	129.44

13	0	129.51
13	0	129.54
13	0	130
13	0	130.68
13	0	130.91
13	0	130.96
13	0	131
13	0	131
13	0	132.75
13	0	132.9
13	0	133
13	0	133.6
13	0	134
13	0	134.07
13	0	134.62
13	0	134.79
13	0	134.91
13	0	134.99
13	0	135.26
13	0	135.79
13	0	138
13	0	138.25
13	0	138.34
13	0	138.43
13	0	138.59
13	0	138.62
13	0	139
13	0	139.19
13	0	139.49
13	0	139.54
13	0	139.56
13	0	139.6
13	0	140.4
13	0	141.12
13	0	141.57
13	0	141.94
13	0	141.94
13	0	141.94
13	0	142
13	0	142.26
13	0	143
13	0	143.43
13	0	144.65
13	0	146
13	0	146
13	0	146.54
13	0	147

13	0	147
13	0	147.66
13	0	150
13	0	154
13	1	85.59
13	1	90.93
13	1	91.84
13	1	91.86
13	1	92.04
13	1	93.37
13	1	93.58
13	1	94.98
13	1	96.84
13	1	97.17
13	1	97.23
13	1	97.29
13	1	97.81
13	1	97.92
13	1	98.5
13	1	98.57
13	1	99.41
13	1	99.65
13	1	99.74
13	1	99.88
13	1	99.96
13	1	100.21
13	1	100.27
13	1	100.32
13	1	100.43
13	1	100.7
13	1	100.91
13	1	101.02
13	1	101.27
13	1	101.28
13	1	101.64
13	1	101.82
13	1	101.83
13	1	101.98
13	1	102.05
13	1	102.06
13	1	102.52
13	1	102.78
13	1	102.81
13	1	102.9
13	1	102.99
13	1	103.02
13	1	103.35

13	1	103.36
13	1	103.54
13	1	103.99
13	1	104.39
13	1	104.42
13	1	104.47
13	1	104.63
13	1	104.78
13	1	104.83
13	1	105.91
13	1	105.92
13	1	105.99
13	1	105.99
13	1	106.01
13	1	106.08
13	1	106.08
13	1	106.41
13	1	106.43
13	1	106.44
13	1	106.53
13	1	106.55
13	1	106.55
13	1	106.56
13	1	107.01
13	1	107.03
13	1	107.06
13	1	107.22
13	1	107.28
13	1	107.3
13	1	107.41
13	1	107.47
13	1	107.78
13	1	107.94
13	1	108.07
13	1	108.21
13	1	108.28
13	1	108.39
13	1	108.39
13	1	108.43
13	1	108.48
13	1	108.52
13	1	108.53
13	1	108.58
13	1	108.66
13	1	109.06
13	1	109.08
13	1	109.14

13	1	109.22
13	1	109.3
13	1	109.38
13	1	109.56
13	1	109.73
13	1	109.87
13	1	109.91
13	1	110.04
13	1	110.12
13	1	110.19
13	1	110.28
13	1	110.28
13	1	110.35
13	1	110.45
13	1	110.61
13	1	110.66
13	1	110.66
13	1	110.7
13	1	110.73
13	1	111.12
13	1	111.27
13	1	111.43
13	1	111.76
13	1	111.82
13	1	111.94
13	1	112.06
13	1	112.31
13	1	112.32
13	1	112.36
13	1	112.4
13	1	112.42
13	1	112.58
13	1	112.61
13	1	112.62
13	1	112.66
13	1	112.66
13	1	112.74
13	1	112.86
13	1	112.95
13	1	112.98
13	1	113.06
13	1	113.1
13	1	113.14
13	1	113.23
13	1	113.24
13	1	113.31
13	1	113.54

13	1	113.56
13	1	113.58
13	1	113.59
13	1	113.61
13	1	113.75
13	1	113.88
13	1	114.09
13	1	114.11
13	1	114.16
13	1	114.34
13	1	114.51
13	1	114.58
13	1	114.58
13	1	114.64
13	1	114.67
13	1	114.7
13	1	114.7
13	1	114.77
13	1	114.78
13	1	114.88
13	1	114.89
13	1	115.02
13	1	115.21
13	1	115.39
13	1	115.4
13	1	115.42
13	1	115.51
13	1	115.52
13	1	115.54
13	1	115.63
13	1	115.64
13	1	115.66
13	1	115.7
13	1	116.02
13	1	116.13
13	1	116.19
13	1	116.21
13	1	116.46
13	1	116.51
13	1	116.52
13	1	116.76
13	1	116.92
13	1	117.07
13	1	117.11
13	1	117.15
13	1	117.23
13	1	117.27

13	1	117.53
13	1	117.55
13	1	117.62
13	1	117.62
13	1	117.68
13	1	117.69
13	1	117.9
13	1	117.99
13	1	118.59
13	1	118.64
13	1	118.74
13	1	118.86
13	1	119
13	1	119.01
13	1	119.05
13	1	119.06
13	1	119.09
13	1	119.12
13	1	119.15
13	1	119.22
13	1	119.82
13	1	119.83
13	1	119.92
13	1	119.95
13	1	119.97
13	1	120.2
13	1	120.73
13	1	120.78
13	1	121.12
13	1	121.21
13	1	121.3
13	1	121.66
13	1	121.68
13	1	121.85
13	1	121.85
13	1	121.95
13	1	121.99
13	1	122.22
13	1	122.34
13	1	122.48
13	1	122.74
13	1	123.42
13	1	123.8
13	1	124.08
13	1	124.13
13	1	124.18
13	1	124.34

13	1	124.38
13	1	124.46
13	1	124.89
13	1	125.05
13	1	125.09
13	1	125.33
13	1	125.47
13	1	125.79
13	1	125.82
13	1	125.98
13	1	126.37
13	1	126.61
13	1	126.65
13	1	126.75
13	1	126.88
13	1	126.98
13	1	127.07
13	1	127.1
13	1	127.36
13	1	127.38
13	1	127.44
13	1	127.45
13	1	127.47
13	1	127.66
13	1	128
13	1	128
13	1	128
13	1	128
13	1	128
13	1	128
13	1	128.4
13	1	128.5
13	1	128.71
13	1	128.96
13	1	129
13	1	129
13	1	129.6
13	1	130
13	1	130.35
13	1	130.44
13	1	130.66
13	1	130.69
13	1	130.88
13	1	131
13	1	131
13	1	131.28
13	1	131.28

13	1	131.43
13	1	131.99
13	1	132
13	1	132.21
13	1	132.28
13	1	132.38
13	1	133.28
13	1	133.54
13	1	133.6
13	1	133.81
13	1	134
13	1	134
13	1	134
13	1	134
13	1	134.46
13	1	134.66
13	1	134.82
13	1	135.07
13	1	136
13	1	136
13	1	136.16
13	1	136.22
13	1	137
13	1	137.01
13	1	137.19
13	1	137.5
13	1	137.59
13	1	142.04
13	1	142.32
13	1	157.13
14	0	51
14	0	74
14	0	75.17
14	0	76
14	0	81.31
14	0	81.7
14	0	82.15
14	0	82.88
14	0	83.07
14	0	83.75
14	0	83.86
14	0	85.38
14	0	85.82
14	0	86.04
14	0	86.04
14	0	86.22
14	0	87.11

14	0	87.17
14	0	87.26
14	0	87.26
14	0	87.32
14	0	87.66
14	0	87.7
14	0	87.82
14	0	88.1
14	0	88.35
14	0	88.42
14	0	88.7
14	0	89.28
14	0	89.79
14	0	90.05
14	0	90.2
14	0	90.63
14	0	90.74
14	0	91.53
14	0	92.72
14	0	92.97
14	0	93.48
14	0	93.63
14	0	94.15
14	0	94.36
14	0	94.49
14	0	94.88
14	0	95.09
14	0	95.27
14	0	95.93
14	0	96.92
14	0	97.2
14	0	97.5
14	0	97.84
14	0	97.84
14	0	97.92
14	0	97.95
14	0	98.38
14	0	98.69
14	0	98.74
14	0	99.16
14	0	99.46
14	0	100.19
14	0	100.36
14	0	100.8
14	0	100.8
14	0	101.31
14	0	101.61

14	0	101.74
14	0	101.88
14	0	102.66
14	0	103
14	0	103.17
14	0	103.31
14	0	103.55
14	0	103.72
14	0	104.95
14	0	104.97
14	0	105
14	0	105.16
14	0	105.28
14	0	105.28
14	0	105.54
14	0	105.6
14	0	105.65
14	0	105.66
14	0	105.68
14	0	105.73
14	0	105.77
14	0	105.86
14	0	105.86
14	0	105.88
14	0	106.6
14	0	106.8
14	0	106.81
14	0	106.91
14	0	106.99
14	0	107.2
14	0	107.61
14	0	107.79
14	0	107.85
14	0	108.01
14	0	108.58
14	0	108.71
14	0	108.93
14	0	109.08
14	0	109.09
14	0	109.71
14	0	110.28
14	0	110.6
14	0	111.39
14	0	111.45
14	0	111.64
14	0	111.69
14	0	112.11

14	0	112.26
14	0	112.34
14	0	112.48
14	0	112.49
14	0	112.71
14	0	112.71
14	0	112.81
14	0	113.07
14	0	113.17
14	0	113.2
14	0	113.22
14	0	113.31
14	0	113.33
14	0	113.44
14	0	113.63
14	0	113.64
14	0	114.15
14	0	117.55
14	0	118.28
14	0	118.58
14	0	119.1
14	0	119.23
14	0	119.77
14	0	119.79
14	0	120.74
14	0	122.86
14	0	123.02
14	0	124.15
14	0	125.69
14	0	125.74
14	0	126.12
14	0	126.7
14	0	127.55
14	0	127.55
14	0	127.78
14	0	128
14	0	132.21
14	0	133
14	0	133.09
14	0	133.74
14	0	139.35
14	0	150
14	1	109.46
14	1	109.91
14	1	77.28
14	1	79.2
14	1	80

14	1	80
14	1	80.14
14	1	80.7
14	1	81.55
14	1	83.21
14	1	83.31
14	1	83.46
14	1	84.82
14	1	84.95
14	1	85.26
14	1	85.42
14	1	85.74
14	1	88.65
14	1	89.27
14	1	89.5
14	1	89.75
14	1	90.82
14	1	91.05
14	1	91.13
14	1	91.36
14	1	91.43
14	1	91.73
14	1	91.88
14	1	93.32
14	1	93.48
14	1	93.97
14	1	94.07
14	1	94.11
14	1	94.13
14	1	94.27
14	1	94.29
14	1	94.96
14	1	94.98
14	1	94.98
14	1	95.08
14	1	95.1
14	1	95.19
14	1	95.31
14	1	95.31
14	1	95.68
14	1	95.74
14	1	95.75
14	1	96.09
14	1	96.28
14	1	96.72
14	1	96.78
14	1	97.16

14	1	97.18
14	1	97.18
14	1	97.32
14	1	97.74
14	1	98.03
14	1	98.14
14	1	98.32
14	1	98.47
14	1	98.73
14	1	98.76
14	1	99.4
14	1	99.48
14	1	99.55
14	1	99.73
14	1	99.82
14	1	99.86
14	1	99.98
14	1	100.1
14	1	100.2
14	1	100.27
14	1	100.7
14	1	100.7
14	1	100.81
14	1	101.14
14	1	101.35
14	1	101.74
14	1	101.8
14	1	101.82
14	1	102.22
14	1	102.22
14	1	102.26
14	1	102.3
14	1	102.34
14	1	102.34
14	1	102.41
14	1	102.6
14	1	102.71
14	1	102.78
14	1	102.86
14	1	102.96
14	1	102.98
14	1	103.14
14	1	103.15
14	1	103.46
14	1	103.57
14	1	103.66
14	1	103.68

14	1	103.68
14	1	103.82
14	1	104.05
14	1	104.47
14	1	104.56
14	1	104.6
14	1	104.84
14	1	104.9
14	1	105.95
14	1	105.97
14	1	105.97
14	1	106.05
14	1	106.19
14	1	106.41
14	1	106.54
14	1	106.59
14	1	106.87
14	1	107.07
14	1	107.19
14	1	107.31
14	1	107.4
14	1	107.69
14	1	107.71
14	1	107.87
14	1	107.91
14	1	107.92
14	1	107.95
14	1	108.17
14	1	108.21
14	1	108.48
14	1	108.51
14	1	108.9
14	1	108.94
14	1	109.53
14	1	109.6
14	1	109.77
14	1	109.83
14	1	109.83
14	1	109.86
14	1	109.96
14	1	109.98
14	1	110.55
14	1	110.56
14	1	110.64
14	1	110.72
14	1	110.74
14	1	110.85

14	1	110.92
14	1	110.93
14	1	110.96
14	1	111.17
14	1	111.38
14	1	111.54
14	1	111.74
14	1	111.92
14	1	111.92
14	1	111.92
14	1	111.92
14	1	111.94
14	1	112.1
14	1	112.14
14	1	112.22
14	1	112.29
14	1	112.32
14	1	112.35
14	1	112.38
14	1	112.56
14	1	112.6
14	1	112.62
14	1	112.65
14	1	112.86
14	1	112.87
14	1	113.13
14	1	113.21
14	1	113.32
14	1	113.34
14	1	113.47
14	1	113.5
14	1	113.9
14	1	113.96
14	1	113.96
14	1	114.04
14	1	114.05
14	1	114.33
14	1	114.38
14	1	114.52
14	1	114.55
14	1	114.62
14	1	114.84
14	1	114.93
14	1	115.03
14	1	115.06
14	1	115.11
14	1	115.15

14	1	115.19
14	1	115.42
14	1	115.6
14	1	115.6
14	1	115.68
14	1	115.74
14	1	115.78
14	1	115.87
14	1	115.98
14	1	116.17
14	1	116.21
14	1	116.22
14	1	116.26
14	1	116.6
14	1	116.6
14	1	116.7
14	1	116.77
14	1	116.87
14	1	117.08
14	1	117.19
14	1	117.24
14	1	117.28
14	1	117.28
14	1	117.47
14	1	117.66
14	1	117.95
14	1	118.03
14	1	118.18
14	1	118.31
14	1	118.41
14	1	118.41
14	1	118.43
14	1	118.86
14	1	118.87
14	1	119.04
14	1	119.08
14	1	119.24
14	1	119.45
14	1	119.54
14	1	119.85
14	1	119.88
14	1	120.01
14	1	120.12
14	1	120.14
14	1	120.3
14	1	120.73
14	1	121.12

14	1	121.26
14	1	121.28
14	1	121.33
14	1	121.64
14	1	121.7
14	1	122.13
14	1	122.21
14	1	122.41
14	1	122.68
14	1	122.82
14	1	123.34
14	1	124.19
14	1	124.57
14	1	124.64
14	1	125.52
14	1	125.71
14	1	125.74
14	1	125.81
14	1	125.84
14	1	126.09
14	1	126.17
14	1	126.31
14	1	126.61
14	1	126.68
14	1	126.99
14	1	127.13
14	1	127.22
14	1	127.24
14	1	127.42
14	1	127.49
14	1	127.57
14	1	127.98
14	1	128
14	1	128
14	1	128
14	1	128.94
14	1	129.01
14	1	129.03
14	1	129.82
14	1	130
14	1	130
14	1	130.65
14	1	130.94
14	1	131.15
14	1	132.18
14	1	133
14	1	133

14	1	135.13
14	1	135.19
14	1	135.93
14	1	136
15	0	79.45
15	0	80.02
15	0	81
15	0	81
15	0	81.66
15	0	81.78
15	0	82.01
15	0	84.45
15	0	85.42
15	0	85.55
15	0	85.92
15	0	86.63
15	0	86.76
15	0	86.94
15	0	87.08
15	0	87.19
15	0	87.55
15	0	87.8
15	0	87.98
15	0	88.25
15	0	88.8
15	0	89.96
15	0	89.98
15	0	89.99
15	0	90.16
15	0	90.4
15	0	90.75
15	0	92.19
15	0	92.64
15	0	92.8
15	0	93.07
15	0	93.3
15	0	93.52
15	0	93.59
15	0	93.61
15	0	93.8
15	0	93.84
15	0	94.06
15	0	94.18
15	0	94.49
15	0	94.55
15	0	95.18
15	0	95.61

15	0	95.82
15	0	95.84
15	0	95.9
15	0	96.18
15	0	96.34
15	0	96.93
15	0	97.29
15	0	97.3
15	0	97.52
15	0	97.81
15	0	97.93
15	0	98.07
15	0	98.37
15	0	98.45
15	0	98.51
15	0	98.59
15	0	98.62
15	0	98.63
15	0	98.85
15	0	98.92
15	0	99.64
15	0	99.76
15	0	100.45
15	0	100.5
15	0	100.56
15	0	100.71
15	0	100.86
15	0	101.07
15	0	101.22
15	0	101.47
15	0	101.58
15	0	101.74
15	0	101.88
15	0	102.17
15	0	102.34
15	0	102.52
15	0	102.88
15	0	103.18
15	0	103.19
15	0	103.6
15	0	103.85
15	0	103.88
15	0	104.02
15	0	104.1
15	0	104.15
15	0	104.52
15	0	104.56

15	0	104.58
15	0	104.92
15	0	105.13
15	0	105.14
15	0	105.15
15	0	105.32
15	0	105.43
15	0	105.63
15	0	105.75
15	0	105.77
15	0	105.8
15	0	105.9
15	0	106.85
15	0	107.01
15	0	107.19
15	0	107.52
15	0	107.82
15	0	108.04
15	0	108.1
15	0	108.24
15	0	108.42
15	0	108.95
15	0	109.5
15	0	109.52
15	0	109.76
15	0	109.9
15	0	110
15	0	110.53
15	0	110.55
15	0	111.14
15	0	111.22
15	0	111.58
15	0	111.6
15	0	112.02
15	0	112.09
15	0	112.27
15	0	112.42
15	0	112.45
15	0	112.68
15	0	112.85
15	0	112.86
15	0	113.15
15	0	113.51
15	0	114.01
15	0	117.5
15	0	118.58
15	0	118.8

15	0	119.49
15	0	120
15	0	121.78
15	0	123.02
15	0	123.43
15	0	123.57
15	0	125.9
15	0	125.93
15	0	126.23
15	0	126.86
15	0	128.84
15	0	129
15	0	130.38
15	0	130.72
15	0	139.16
15	0	141.97
15	0	143.49
15	0	143.75
15	0	146.87
15	1	84.82
15	1	89.17
15	1	89.76
15	1	90.25
15	1	91.14
15	1	91.3
15	1	91.84
15	1	92.39
15	1	92.46
15	1	93.05
15	1	93.47
15	1	93.48
15	1	93.53
15	1	93.67
15	1	93.68
15	1	93.74
15	1	93.88
15	1	94.09
15	1	94.12
15	1	94.45
15	1	94.67
15	1	94.68
15	1	94.89
15	1	94.91
15	1	95.11
15	1	95.25
15	1	95.33
15	1	95.38

15	1	95.39
15	1	95.62
15	1	96.24
15	1	96.46
15	1	96.5
15	1	96.52
15	1	96.73
15	1	96.74
15	1	96.75
15	1	96.95
15	1	96.97
15	1	96.99
15	1	97.02
15	1	97.03
15	1	97.23
15	1	97.28
15	1	97.64
15	1	97.81
15	1	97.86
15	1	97.88
15	1	97.93
15	1	98.03
15	1	98.06
15	1	98.13
15	1	98.18
15	1	98.38
15	1	98.81
15	1	98.85
15	1	99.03
15	1	99.16
15	1	99.18
15	1	99.25
15	1	99.27
15	1	99.31
15	1	99.4
15	1	99.67
15	1	99.74
15	1	99.94
15	1	100.02
15	1	100.29
15	1	100.29
15	1	100.41
15	1	100.5
15	1	100.51
15	1	100.61
15	1	100.63
15	1	100.73

15	1	100.8
15	1	100.91
15	1	101.07
15	1	101.2
15	1	101.21
15	1	101.32
15	1	101.39
15	1	101.43
15	1	101.45
15	1	101.48
15	1	101.59
15	1	101.73
15	1	101.95
15	1	102
15	1	102.04
15	1	102.12
15	1	102.2
15	1	102.34
15	1	102.49
15	1	102.52
15	1	102.56
15	1	102.66
15	1	102.75
15	1	102.75
15	1	102.77
15	1	102.78
15	1	102.95
15	1	102.97
15	1	103.03
15	1	103.03
15	1	103.04
15	1	103.05
15	1	103.16
15	1	103.28
15	1	103.38
15	1	103.4
15	1	103.59
15	1	103.74
15	1	103.83
15	1	103.96
15	1	104.35
15	1	104.37
15	1	104.44
15	1	104.49
15	1	104.62
15	1	104.67
15	1	104.88

15	1	105.95
15	1	105.98
15	1	106.01
15	1	106.03
15	1	106.22
15	1	106.22
15	1	106.37
15	1	106.38
15	1	106.4
15	1	106.48
15	1	106.58
15	1	106.62
15	1	106.76
15	1	106.82
15	1	106.83
15	1	106.88
15	1	106.97
15	1	106.99
15	1	107.02
15	1	107.03
15	1	107.05
15	1	107.21
15	1	107.31
15	1	107.39
15	1	107.47
15	1	107.48
15	1	107.49
15	1	107.57
15	1	107.61
15	1	107.69
15	1	107.76
15	1	107.77
15	1	107.8
15	1	107.87
15	1	107.88
15	1	108
15	1	108.03
15	1	108.03
15	1	108.15
15	1	108.2
15	1	108.31
15	1	108.35
15	1	108.49
15	1	108.74
15	1	108.84
15	1	108.84
15	1	108.89

15	1	109.07
15	1	109.17
15	1	109.3
15	1	109.36
15	1	109.54
15	1	109.67
15	1	109.7
15	1	110.11
15	1	110.13
15	1	110.17
15	1	110.18
15	1	110.32
15	1	110.41
15	1	110.55
15	1	110.67
15	1	110.98
15	1	111.17
15	1	111.32
15	1	111.34
15	1	111.77
15	1	111.86
15	1	112.07
15	1	112.38
15	1	112.42
15	1	112.46
15	1	112.66
15	1	112.66
15	1	113.47
15	1	113.67
15	1	113.75
15	1	113.77
15	1	113.78
15	1	113.84
15	1	113.88
15	1	113.9
15	1	114.25
15	1	114.26
15	1	114.28
15	1	114.33
15	1	114.38
15	1	114.5
15	1	114.67
15	1	114.71
15	1	114.8
15	1	115
15	1	115.04
15	1	115.12

15	1	115.48
15	1	115.8
15	1	115.84
15	1	115.88
15	1	115.9
15	1	115.93
15	1	116.05
15	1	116.06
15	1	116.2
15	1	116.24
15	1	116.27
15	1	116.27
15	1	116.43
15	1	116.51
15	1	116.55
15	1	116.65
15	1	116.89
15	1	116.93
15	1	116.95
15	1	117.11
15	1	117.25
15	1	117.45
15	1	117.51
15	1	117.68
15	1	117.8
15	1	117.89
15	1	118.21
15	1	118.47
15	1	118.61
15	1	118.66
15	1	118.88
15	1	118.98
15	1	119.05
15	1	119.31
15	1	119.43
15	1	119.74
15	1	119.79
15	1	119.98
15	1	120.06
15	1	120.08
15	1	120.19
15	1	120.47
15	1	120.55
15	1	120.97
15	1	120.97
15	1	121.01
15	1	121.04

15	1	121.09
15	1	121.32
15	1	121.4
15	1	121.56
15	1	121.83
15	1	122.02
15	1	122.04
15	1	122.86
15	1	123.06
15	1	123.11
15	1	123.3
15	1	123.34
15	1	123.49
15	1	123.66
15	1	123.66
15	1	123.71
15	1	124
15	1	124.05
15	1	124.55
15	1	124.83
15	1	124.83
15	1	125.06
15	1	125.52
15	1	125.64
15	1	125.73
15	1	126.12
15	1	127.01
15	1	127.08
15	1	127.4
15	1	127.5
15	1	127.93
15	1	127.97
15	1	128
15	1	128
15	1	128.56
15	1	129.47
15	1	130
15	1	132
15	1	133.35
15	1	133.53
15	1	133.84
15	1	136
16	0	90.92
16	0	92.38
16	0	93.53
16	0	95.31
16	0	95.9

16	0	96.69
16	0	98.06
16	0	101.7
16	0	103.83
16	0	104.15
16	0	104.44
16	0	104.53
16	0	106.23
16	0	106.73
16	0	106.86
16	0	107.49
16	0	108
16	0	108.31
16	0	108.35
16	0	109.45
16	0	109.52
16	0	109.68
16	0	109.73
16	0	109.75
16	0	109.79
16	0	110
16	0	110.05
16	0	110.16
16	0	111
16	0	111.09
16	0	111.63
16	0	112.75
16	0	113.77
16	0	116.02
16	0	117.42
16	0	118.37
16	0	118.57
16	0	118.71
16	0	118.82
16	0	119.02
16	0	119.18
16	0	119.43
16	0	119.48
16	0	119.86
16	0	119.95
16	0	119.96
16	0	120
16	0	120.06
16	0	120.63
16	0	120.64
16	0	120.72
16	0	120.93

16	0	121.05
16	0	121.07
16	0	121.08
16	0	121.3
16	0	121.39
16	0	121.39
16	0	121.65
16	0	121.74
16	0	121.76
16	0	122.23
16	0	122.35
16	0	122.37
16	0	122.41
16	0	122.51
16	0	122.56
16	0	122.68
16	0	122.69
16	0	122.76
16	0	122.95
16	0	123.35
16	0	123.38
16	0	123.5
16	0	123.54
16	0	123.56
16	0	123.63
16	0	123.7
16	0	124.4
16	0	124.42
16	0	124.43
16	0	124.61
16	0	124.66
16	0	125
16	0	125.06
16	0	125.11
16	0	125.19
16	0	125.25
16	0	125.31
16	0	125.34
16	0	125.36
16	0	125.51
16	0	125.54
16	0	125.74
16	0	125.78
16	0	126.08
16	0	126.12
16	0	126.19
16	0	126.41

16	0	126.55
16	0	126.64
16	0	126.67
16	0	126.77
16	0	126.8
16	0	126.94
16	0	127
16	0	127.04
16	0	127.05
16	0	127.13
16	0	127.35
16	0	127.39
16	0	127.42
16	0	127.7
16	0	127.71
16	0	127.73
16	0	127.83
16	0	127.83
16	0	127.9
16	0	128
16	0	128
16	0	128
16	0	128
16	0	128
16	0	128
16	0	128.31
16	0	128.37
16	0	128.53
16	0	128.57
16	0	128.82
16	0	128.97
16	0	129
16	0	129
16	0	129
16	0	129
16	0	129
16	0	129
16	0	129.01
16	0	129.22
16	0	129.35
16	0	129.47
16	0	129.5
16	0	130
16	0	130
16	0	130
16	0	130
16	0	130.31

16	0	130.62
16	0	130.74
16	0	130.76
16	0	131
16	0	131
16	0	131
16	0	131.28
16	0	131.32
16	0	131.4
16	0	131.62
16	0	131.68
16	0	131.78
16	0	132
16	0	132
16	0	132.01
16	0	132.38
16	0	132.6
16	0	133
16	0	133
16	0	133
16	0	133
16	0	133
16	0	133
16	0	133
16	0	133.03
16	0	133.18
16	0	133.51
16	0	133.75
16	0	133.99
16	0	134
16	0	134.22
16	0	134.81
16	0	134.91
16	0	135
16	0	135
16	0	135
16	0	135.13
16	0	135.18
16	0	135.25
16	0	135.43
16	0	135.71
16	0	135.9
16	0	136
16	0	136
16	0	136.25
16	0	136.31
16	0	136.41

16	0	136.65
16	0	137
16	0	137.12
16	0	137.4
16	0	137.5
16	0	138
16	0	138
16	0	138
16	0	138.01
16	0	138.21
16	0	138.4
16	0	138.41
16	0	138.54
16	0	138.6
16	0	138.62
16	0	138.69
16	0	138.74
16	0	138.75
16	0	138.91
16	0	138.94
16	0	139.04
16	0	139.12
16	0	139.25
16	0	139.34
16	0	139.81
16	0	140
16	0	140
16	0	140.21
16	0	140.49
16	0	140.51
16	0	140.63
16	0	140.68
16	0	140.81
16	0	141
16	0	141
16	0	141
16	0	141.18
16	0	141.54
16	0	141.63
16	0	141.65
16	0	141.94
16	0	142
16	0	142
16	0	142.99
16	0	143
16	0	143
16	0	143.68

16	0	144
16	0	144.04
16	0	144.06
16	0	144.28
16	0	145.44
16	0	145.93
16	0	146
16	0	146.1
16	0	146.43
16	0	147
16	0	147.21
16	0	147.66
16	0	148.28
16	0	148.37
16	0	148.6
16	0	149.79
16	0	150
16	0	151
16	0	152.04
16	1	89.73
16	1	94.51
16	1	95.44
16	1	97.53
16	1	101.8
16	1	102.7
16	1	103.44
16	1	103.84
16	1	106.3
16	1	106.4
16	1	106.62
16	1	106.73
16	1	107.57
16	1	107.58
16	1	108.12
16	1	108.41
16	1	108.66
16	1	108.96
16	1	109.44
16	1	109.52
16	1	109.59
16	1	109.91
16	1	109.93
16	1	109.94
16	1	110.02
16	1	110.19
16	1	110.25
16	1	110.28

16	1	110.48
16	1	110.66
16	1	111
16	1	111.34
16	1	111.46
16	1	111.49
16	1	111.53
16	1	111.82
16	1	111.93
16	1	112.03
16	1	112.03
16	1	112.08
16	1	112.18
16	1	112.24
16	1	112.47
16	1	112.85
16	1	112.92
16	1	113.35
16	1	113.46
16	1	113.5
16	1	113.6
16	1	113.81
16	1	113.91
16	1	114
16	1	114.1
16	1	114.16
16	1	114.23
16	1	114.33
16	1	114.37
16	1	114.5
16	1	114.55
16	1	115.1
16	1	115.18
16	1	115.27
16	1	115.29
16	1	115.63
16	1	115.64
16	1	115.67
16	1	115.8
16	1	115.8
16	1	115.85
16	1	116.01
16	1	116.13
16	1	116.61
16	1	116.79
16	1	116.84
16	1	116.86

16	1	116.94
16	1	116.94
16	1	116.95
16	1	117.22
16	1	117.23
16	1	117.25
16	1	117.26
16	1	117.34
16	1	117.35
16	1	117.44
16	1	117.44
16	1	117.6
16	1	117.72
16	1	117.76
16	1	117.81
16	1	117.81
16	1	117.98
16	1	117.98
16	1	117.99
16	1	118.19
16	1	118.35
16	1	118.35
16	1	118.39
16	1	118.44
16	1	118.49
16	1	118.52
16	1	118.52
16	1	118.57
16	1	118.62
16	1	118.65
16	1	118.86
16	1	118.87
16	1	118.95
16	1	118.97
16	1	119.04
16	1	119.05
16	1	119.11
16	1	119.23
16	1	119.34
16	1	119.38
16	1	119.52
16	1	119.57
16	1	119.58
16	1	119.66
16	1	119.97
16	1	120.01
16	1	120.17

16	1	120.37
16	1	120.43
16	1	120.58
16	1	120.64
16	1	120.67
16	1	120.71
16	1	120.81
16	1	121
16	1	121.04
16	1	121.1
16	1	121.11
16	1	121.12
16	1	121.24
16	1	121.33
16	1	121.41
16	1	121.6
16	1	121.62
16	1	121.7
16	1	121.89
16	1	122
16	1	122.21
16	1	122.31
16	1	122.64
16	1	122.71
16	1	122.79
16	1	122.81
16	1	123.16
16	1	123.23
16	1	123.34
16	1	123.53
16	1	123.7
16	1	123.74
16	1	123.82
16	1	123.86
16	1	123.9
16	1	124.14
16	1	124.14
16	1	124.26
16	1	124.31
16	1	124.32
16	1	124.46
16	1	124.54
16	1	124.56
16	1	124.62
16	1	124.8
16	1	124.94
16	1	125.17

16	1	125.25
16	1	125.27
16	1	125.35
16	1	125.4
16	1	125.41
16	1	125.58
16	1	125.62
16	1	125.82
16	1	125.89
16	1	125.91
16	1	126.02
16	1	126.16
16	1	126.24
16	1	126.25
16	1	126.34
16	1	126.43
16	1	126.63
16	1	126.78
16	1	126.83
16	1	126.87
16	1	126.89
16	1	127.07
16	1	127.1
16	1	127.16
16	1	127.24
16	1	127.41
16	1	127.42
16	1	127.42
16	1	127.47
16	1	127.92
16	1	128
16	1	128
16	1	128
16	1	128
16	1	128.01
16	1	128.06
16	1	128.13
16	1	128.26
16	1	128.44
16	1	128.49
16	1	129
16	1	129
16	1	129
16	1	129
16	1	129.12
16	1	129.24
16	1	129.4

16	1	129.43
16	1	129.47
16	1	130
16	1	130
16	1	130
16	1	130.34
16	1	130.62
16	1	130.88
16	1	131
16	1	131
16	1	131.1
16	1	131.34
16	1	131.76
16	1	131.82
16	1	131.85
16	1	131.97
16	1	132
16	1	132
16	1	132
16	1	132.06
16	1	132.22
16	1	132.24
16	1	132.46
16	1	132.68
16	1	132.94
16	1	133
16	1	133
16	1	133
16	1	133.63
16	1	133.65
16	1	133.74
16	1	133.9
16	1	134
16	1	134
16	1	134.06
16	1	134.16
16	1	134.26
16	1	134.44
16	1	134.47
16	1	134.57
16	1	134.85
16	1	134.9
16	1	135
16	1	135
16	1	135
16	1	135
16	1	135.06

16	1	135.06
16	1	135.18
16	1	135.26
16	1	135.34
16	1	135.34
16	1	135.41
16	1	136.18
16	1	136.29
16	1	136.35
16	1	137
16	1	137
16	1	142
16	1	142.63
16	1	142.78
16	1	155.46
16	1	156
16	1	159.51
16	1	163.75
17	0	77.89
17	0	82.24
17	0	83.4
17	0	83.54
17	0	83.94
17	0	84.61
17	0	84.88
17	0	85.11
17	0	85.45
17	0	86.04
17	0	86.25
17	0	86.37
17	0	87.08
17	0	87.18
17	0	87.25
17	0	87.26
17	0	87.63
17	0	88.1
17	0	88.41
17	0	88.47
17	0	88.58
17	0	88.64
17	0	89.96
17	0	90.65
17	0	91.78
17	0	91.88
17	0	91.94
17	0	92.19
17	0	92.26

17	0	92.28
17	0	92.37
17	0	92.6
17	0	92.74
17	0	93.19
17	0	93.34
17	0	94.39
17	0	94.79
17	0	94.83
17	0	95.51
17	0	95.57
17	0	95.65
17	0	95.75
17	0	96.23
17	0	96.41
17	0	96.43
17	0	96.44
17	0	96.45
17	0	96.64
17	0	96.67
17	0	96.94
17	0	97.14
17	0	97.35
17	0	97.37
17	0	97.67
17	0	98.28
17	0	98.6
17	0	98.67
17	0	98.78
17	0	99.08
17	0	99.11
17	0	99.23
17	0	99.28
17	0	99.37
17	0	99.62
17	0	99.9
17	0	99.91
17	0	100.34
17	0	100.74
17	0	101.41
17	0	101.48
17	0	101.51
17	0	101.51
17	0	101.67
17	0	101.7
17	0	101.92
17	0	102.19

17	0	102.95
17	0	103.2
17	0	103.43
17	0	103.45
17	0	103.57
17	0	103.89
17	0	104.4
17	0	104.8
17	0	104.97
17	0	105.02
17	0	105.09
17	0	105.1
17	0	105.16
17	0	105.24
17	0	105.6
17	0	105.68
17	0	105.76
17	0	105.77
17	0	105.88
17	0	105.89
17	0	106.22
17	0	106.22
17	0	106.65
17	0	106.88
17	0	106.9
17	0	107.03
17	0	107.36
17	0	107.4
17	0	107.45
17	0	107.5
17	0	107.53
17	0	107.67
17	0	107.68
17	0	107.81
17	0	107.83
17	0	107.91
17	0	108.59
17	0	108.67
17	0	108.82
17	0	109.01
17	0	109.05
17	0	109.21
17	0	109.27
17	0	109.51
17	0	110.12
17	0	110.22
17	0	110.57

17	0	110.58
17	0	110.85
17	0	111.22
17	0	111.62
17	0	111.73
17	0	111.92
17	0	112.07
17	0	112.66
17	0	113.29
17	0	113.55
17	0	113.79
17	0	113.82
17	0	116.09
17	0	117.49
17	0	118.29
17	0	118.93
17	0	119.13
17	0	120.13
17	0	120.64
17	0	120.75
17	0	121.51
17	0	123.25
17	0	123.29
17	0	123.45
17	0	124.67
17	0	125
17	0	127.52
17	0	128
17	0	128
17	0	129
17	0	131.19
17	0	138.5
17	0	139
17	0	140.65
17	0	142.85
17	0	148.88
17	0	151
17	0	152.18
17	1	89.91
17	1	92.06
17	1	93.01
17	1	93.92
17	1	94.17
17	1	94.64
17	1	95.44
17	1	95.76
17	1	96.17

17	1	96.34
17	1	96.35
17	1	96.36
17	1	96.78
17	1	96.91
17	1	97.64
17	1	97.76
17	1	97.78
17	1	98.27
17	1	98.54
17	1	99.06
17	1	99.19
17	1	99.49
17	1	99.86
17	1	99.96
17	1	100.18
17	1	100.44
17	1	100.48
17	1	100.67
17	1	101.34
17	1	101.4
17	1	101.67
17	1	101.89
17	1	101.99
17	1	102.28
17	1	102.32
17	1	102.49
17	1	102.57
17	1	102.65
17	1	102.66
17	1	102.67
17	1	102.74
17	1	102.85
17	1	102.91
17	1	102.92
17	1	103.05
17	1	103.22
17	1	103.38
17	1	103.6
17	1	103.73
17	1	103.86
17	1	104.21
17	1	104.39
17	1	104.74
17	1	104.82
17	1	104.85
17	1	104.92

17	1	104.92
17	1	104.93
17	1	104.94
17	1	105.92
17	1	105.99
17	1	105.99
17	1	106.09
17	1	106.14
17	1	106.27
17	1	106.28
17	1	106.29
17	1	106.36
17	1	106.37
17	1	106.45
17	1	106.5
17	1	106.64
17	1	106.64
17	1	106.65
17	1	106.7
17	1	106.83
17	1	107.03
17	1	107.32
17	1	107.37
17	1	107.95
17	1	108.1
17	1	108.35
17	1	108.39
17	1	108.46
17	1	108.46
17	1	108.56
17	1	108.72
17	1	108.92
17	1	108.95
17	1	109.03
17	1	109.19
17	1	109.25
17	1	109.26
17	1	109.28
17	1	109.35
17	1	109.5
17	1	109.53
17	1	109.59
17	1	109.67
17	1	109.75
17	1	109.79
17	1	110.18
17	1	110.19

17	1	110.19
17	1	110.3
17	1	110.34
17	1	110.56
17	1	110.56
17	1	110.61
17	1	110.62
17	1	110.67
17	1	110.74
17	1	110.86
17	1	110.89
17	1	110.89
17	1	111.01
17	1	111.14
17	1	111.16
17	1	111.16
17	1	111.25
17	1	111.29
17	1	111.33
17	1	111.59
17	1	111.74
17	1	111.9
17	1	111.93
17	1	112.06
17	1	112.2
17	1	112.23
17	1	112.3
17	1	112.34
17	1	112.34
17	1	112.42
17	1	112.47
17	1	112.55
17	1	112.67
17	1	113.39
17	1	113.85
17	1	113.9
17	1	114.06
17	1	114.09
17	1	114.12
17	1	114.21
17	1	114.23
17	1	114.36
17	1	114.37
17	1	114.38
17	1	114.38
17	1	114.49
17	1	114.52

17	1	114.63
17	1	114.66
17	1	114.67
17	1	114.95
17	1	115.09
17	1	115.1
17	1	115.14
17	1	115.17
17	1	115.3
17	1	115.32
17	1	115.34
17	1	115.44
17	1	115.52
17	1	115.64
17	1	115.68
17	1	115.76
17	1	115.78
17	1	115.8
17	1	115.83
17	1	115.84
17	1	115.87
17	1	115.9
17	1	116.1
17	1	116.13
17	1	116.22
17	1	116.27
17	1	116.41
17	1	116.43
17	1	116.51
17	1	116.53
17	1	116.54
17	1	116.54
17	1	116.55
17	1	116.58
17	1	116.59
17	1	116.68
17	1	116.71
17	1	116.73
17	1	116.75
17	1	116.75
17	1	116.8
17	1	116.98
17	1	117.03
17	1	117.05
17	1	117.05
17	1	117.06
17	1	117.14

17	1	117.36
17	1	117.4
17	1	117.67
17	1	117.68
17	1	117.8
17	1	117.8
17	1	117.89
17	1	118
17	1	118.11
17	1	118.2
17	1	118.35
17	1	118.36
17	1	118.59
17	1	118.6
17	1	118.63
17	1	118.67
17	1	118.81
17	1	118.82
17	1	119.13
17	1	119.25
17	1	119.41
17	1	119.41
17	1	119.47
17	1	119.55
17	1	119.68
17	1	119.76
17	1	119.83
17	1	119.83
17	1	119.97
17	1	120.32
17	1	120.35
17	1	120.53
17	1	120.64
17	1	120.65
17	1	120.73
17	1	120.81
17	1	120.85
17	1	120.86
17	1	121.11
17	1	121.24
17	1	121.28
17	1	121.32
17	1	121.44
17	1	121.47
17	1	121.52
17	1	121.73
17	1	121.83

17	1	121.95
17	1	121.98
17	1	122.2
17	1	122.23
17	1	122.53
17	1	122.56
17	1	122.66
17	1	122.87
17	1	122.91
17	1	123.13
17	1	123.73
17	1	123.74
17	1	123.74
17	1	123.74
17	1	123.89
17	1	124.31
17	1	124.62
17	1	124.64
17	1	124.74
17	1	125.13
17	1	125.39
17	1	125.43
17	1	125.47
17	1	125.6
17	1	125.6
17	1	126.04
17	1	126.17
17	1	126.22
17	1	126.33
17	1	126.47
17	1	126.56
17	1	126.57
17	1	126.57
17	1	126.97
17	1	127.13
17	1	127.3
17	1	127.52
17	1	127.63
17	1	128
17	1	128
17	1	128.35
17	1	128.38
17	1	128.62
17	1	129
17	1	129.68
17	1	130
17	1	130.57

17	1	131
17	1	131.31
17	1	132
17	1	132.28
17	1	132.43
17	1	132.91
17	1	133
17	1	133.07
17	1	133.76
17	1	133.94
17	1	134
17	1	134.03
17	1	134.28
17	1	135
17	1	135.16
17	1	135.43
17	1	135.54
17	1	137.01
17	1	137.51
17	1	142
17	1	142.01
19	0	65
19	0	71
19	0	71
19	0	73
19	0	73.39
19	0	73.81
19	0	74.31
19	0	74.33
19	0	75.06
19	0	76
19	0	76
19	0	76.86
19	0	81.56
19	0	81.72
19	0	81.94
19	0	82.11
19	0	82.65
19	0	82.8
19	0	84.38
19	0	85.9
19	0	85.93
19	0	86.11
19	0	86.19
19	0	86.39
19	0	86.46
19	0	86.49

19	0	86.56
19	0	86.61
19	0	86.99
19	0	87.12
19	0	87.13
19	0	87.34
19	0	87.48
19	0	87.49
19	0	87.52
19	0	87.62
19	0	87.84
19	0	87.89
19	0	88.04
19	0	88.4
19	0	88.46
19	0	88.56
19	0	89.52
19	0	90.01
19	0	90.18
19	0	90.31
19	0	90.5
19	0	90.52
19	0	91.24
19	0	91.67
19	0	93.39
19	0	93.49
19	0	93.53
19	0	94
19	0	94.56
19	0	94.58
19	0	95.58
19	0	96.11
19	0	96.27
19	0	96.54
19	0	97.37
19	0	97.5
19	0	97.68
19	0	98.39
19	0	98.7
19	0	98.73
19	0	98.86
19	0	100.51
19	0	100.71
19	0	101.47
19	0	101.54
19	0	101.7
19	0	101.94

19	0	102.01
19	0	102.11
19	0	102.72
19	0	102.73
19	0	102.83
19	0	102.86
19	0	103.39
19	0	103.45
19	0	103.49
19	0	103.52
19	0	103.73
19	0	103.87
19	0	103.87
19	0	104.04
19	0	104.26
19	0	104.56
19	0	104.56
19	0	104.95
19	0	105
19	0	105.06
19	0	105.15
19	0	105.48
19	0	105.59
19	0	105.75
19	0	105.75
19	0	105.78
19	0	105.8
19	0	106.2
19	0	106.63
19	0	106.68
19	0	107.01
19	0	107.21
19	0	107.42
19	0	107.52
19	0	107.6
19	0	107.69
19	0	108.23
19	0	108.44
19	0	108.65
19	0	108.69
19	0	108.69
19	0	108.77
19	0	108.85
19	0	109.92
19	0	109.95
19	0	110.13
19	0	110.52

19	0	110.8
19	0	110.83
19	0	110.95
19	0	111.13
19	0	111.79
19	0	111.92
19	0	111.99
19	0	112.27
19	0	112.32
19	0	112.32
19	0	113.22
19	0	113.54
19	0	113.55
19	0	117.47
19	0	117.93
19	0	119.51
19	0	119.73
19	0	120.43
19	0	120.65
19	0	121.16
19	0	121.62
19	0	122.89
19	0	124.03
19	0	124.3
19	0	124.56
19	0	130
19	0	136.4
19	0	136.63
19	0	139.29
19	0	139.78
19	0	140
19	0	141.37
19	0	152.1
19	1	78
19	1	78
19	1	78.36
19	1	78.67
19	1	78.73
19	1	79
19	1	79.55
19	1	80
19	1	80.13
19	1	80.81
19	1	81
19	1	81
19	1	81
19	1	81.3

19	1	83.13
19	1	83.29
19	1	83.55
19	1	83.99
19	1	84.27
19	1	84.9
19	1	85.08
19	1	85.11
19	1	85.17
19	1	85.35
19	1	85.45
19	1	85.47
19	1	89.03
19	1	89.1
19	1	89.21
19	1	89.91
19	1	90.41
19	1	90.52
19	1	90.7
19	1	90.97
19	1	91.01
19	1	91.63
19	1	91.68
19	1	91.9
19	1	91.92
19	1	91.97
19	1	92.15
19	1	92.4
19	1	92.41
19	1	92.44
19	1	92.61
19	1	92.72
19	1	92.72
19	1	92.73
19	1	92.81
19	1	92.91
19	1	92.97
19	1	93.01
19	1	93.12
19	1	93.27
19	1	93.39
19	1	93.5
19	1	93.58
19	1	93.65
19	1	94.1
19	1	94.11
19	1	94.2

19	1	94.37
19	1	94.39
19	1	94.53
19	1	94.6
19	1	94.77
19	1	94.8
19	1	95.1
19	1	95.12
19	1	95.14
19	1	95.15
19	1	95.35
19	1	95.51
19	1	95.52
19	1	95.52
19	1	95.6
19	1	95.6
19	1	95.61
19	1	95.78
19	1	95.96
19	1	96.21
19	1	96.22
19	1	96.29
19	1	96.43
19	1	96.47
19	1	96.6
19	1	96.71
19	1	96.77
19	1	96.78
19	1	96.89
19	1	97.4
19	1	97.65
19	1	97.8
19	1	97.9
19	1	98.08
19	1	98.12
19	1	98.13
19	1	98.17
19	1	98.2
19	1	98.23
19	1	98.45
19	1	98.56
19	1	98.57
19	1	98.75
19	1	98.8
19	1	98.81
19	1	98.9
19	1	98.92

19	1	99.05
19	1	99.15
19	1	99.19
19	1	99.27
19	1	99.34
19	1	99.35
19	1	99.41
19	1	99.45
19	1	99.61
19	1	99.78
19	1	99.88
19	1	100.19
19	1	100.68
19	1	100.71
19	1	101
19	1	101.1
19	1	101.11
19	1	101.13
19	1	101.28
19	1	101.45
19	1	101.46
19	1	101.71
19	1	101.89
19	1	101.91
19	1	102.07
19	1	102.2
19	1	102.4
19	1	102.62
19	1	102.71
19	1	103.26
19	1	103.31
19	1	103.31
19	1	103.32
19	1	103.45
19	1	103.5
19	1	103.66
19	1	103.85
19	1	103.89
19	1	104.06
19	1	104.08
19	1	104.09
19	1	104.5
19	1	104.5
19	1	104.59
19	1	104.9
19	1	106.07
19	1	106.24

19	1	106.41
19	1	106.44
19	1	106.47
19	1	106.79
19	1	107.08
19	1	107.13
19	1	107.23
19	1	107.24
19	1	107.39
19	1	107.6
19	1	107.71
19	1	107.71
19	1	108.28
19	1	108.53
19	1	108.9
19	1	109.28
19	1	109.5
19	1	109.63
19	1	109.71
19	1	109.8
19	1	109.89
19	1	109.92
19	1	110.09
19	1	110.17
19	1	110.32
19	1	110.35
19	1	110.93
19	1	110.95
19	1	110.97
19	1	110.99
19	1	111.13
19	1	111.25
19	1	111.71
19	1	111.98
19	1	112.1
19	1	112.36
19	1	112.39
19	1	112.7
19	1	112.73
19	1	112.77
19	1	112.98
19	1	113.27
19	1	113.42
19	1	113.9
19	1	113.97
19	1	114.06
19	1	114.07

19	1	114.7
19	1	114.7
19	1	114.78
19	1	114.81
19	1	114.91
19	1	115.03
19	1	115.13
19	1	115.21
19	1	115.54
19	1	115.55
19	1	115.55
19	1	115.72
19	1	115.8
19	1	115.84
19	1	116.16
19	1	116.45
19	1	116.47
19	1	117.01
19	1	117.33
19	1	117.34
19	1	118.47
19	1	118.56
19	1	119.11
19	1	119.32
19	1	119.39
19	1	119.53
19	1	119.61
19	1	119.65
19	1	119.98
19	1	120.1
19	1	120.11
19	1	120.14
19	1	120.17
19	1	120.19
19	1	120.93
19	1	121.01
19	1	121.04
19	1	121.1
19	1	122.59
19	1	123.32
19	1	123.84
19	1	123.85
19	1	123.96
19	1	123.98
19	1	124.02
19	1	124.41
19	1	124.61

19	1	124.62
19	1	125.35
19	1	125.42
19	1	125.68
19	1	126.26
19	1	126.43
19	1	126.68
19	1	127.03
19	1	127.28
19	1	127.36
19	1	127.59
19	1	127.73
19	1	127.94
19	1	129.01
19	1	129.56
19	1	130.6
19	1	130.65
19	1	131.66
19	1	131.99
19	1	132
19	1	132.6
19	1	132.91
19	1	133.54
19	1	133.79
19	1	133.97
19	1	134
19	1	135.47
19	1	137
19	1	137
19	1	137.47
19	1	137.47
20	0	78
20	0	83.78
20	0	86
20	0	86.12
20	0	86.44
20	0	86.45
20	0	86.59
20	0	86.7
20	0	86.95
20	0	86.97
20	0	87.35
20	0	87.6
20	0	87.71
20	0	88.27
20	0	89.12
20	0	89.99

20	0	90.02
20	0	90.16
20	0	90.66
20	0	90.71
20	0	92.31
20	0	92.33
20	0	94.34
20	0	94.82
20	0	96.04
20	0	97.57
20	0	98.67
20	0	98.69
20	0	98.86
20	0	98.98
20	0	99.1
20	0	100.33
20	0	100.82
20	0	101.07
20	0	101.1
20	0	101.3
20	0	101.31
20	0	101.36
20	0	101.71
20	0	101.73
20	0	102.05
20	0	102.21
20	0	102.28
20	0	102.37
20	0	102.76
20	0	103.05
20	0	103.52
20	0	103.76
20	0	104.21
20	0	104.26
20	0	104.47
20	0	104.72
20	0	104.93
20	0	104.96
20	0	105.03
20	0	105.05
20	0	105.19
20	0	105.53
20	0	105.54
20	0	105.77
20	0	105.83
20	0	105.84
20	0	107.57

20	0	108.29
20	0	108.31
20	0	108.34
20	0	108.5
20	0	108.62
20	0	108.77
20	0	108.92
20	0	109.27
20	0	109.48
20	0	109.59
20	0	109.62
20	0	109.7
20	0	109.73
20	0	109.8
20	0	110.07
20	0	110.26
20	0	110.54
20	0	110.7
20	0	110.87
20	0	110.89
20	0	111.19
20	0	111.27
20	0	111.29
20	0	111.38
20	0	111.7
20	0	112.02
20	0	112.72
20	0	112.85
20	0	112.9
20	0	113.3
20	0	113.33
20	0	113.36
20	0	113.4
20	0	113.42
20	0	113.76
20	0	113.83
20	0	117.51
20	0	117.54
20	0	117.67
20	0	118.22
20	0	118.27
20	0	119.46
20	0	119.54
20	0	119.67
20	0	119.76
20	0	119.79
20	0	120.52

20	0	121.07
20	0	122.26
20	0	122.63
20	0	122.91
20	0	123.47
20	0	123.88
20	0	124.56
20	0	124.7
20	0	126.1
20	0	126.16
20	0	126.5
20	0	126.76
20	0	129
20	0	129.63
20	0	130.57
20	0	130.59
20	0	133
20	0	134.96
20	0	138.49
20	0	139
20	0	140.41
20	0	140.46
20	0	142
20	0	144
20	0	147.19
20	0	148
20	1	81
20	1	83.48
20	1	89.64
20	1	89.74
20	1	89.76
20	1	89.85
20	1	90.35
20	1	90.48
20	1	90.73
20	1	91.33
20	1	91.54
20	1	92.3
20	1	92.34
20	1	92.36
20	1	92.55
20	1	93.29
20	1	93.49
20	1	93.8
20	1	94.02
20	1	94.16
20	1	94.28

20	1	95.32
20	1	96.28
20	1	96.7
20	1	96.8
20	1	96.84
20	1	97.05
20	1	97.12
20	1	97.15
20	1	97.38
20	1	97.71
20	1	97.71
20	1	97.81
20	1	98.18
20	1	98.55
20	1	98.89
20	1	99.22
20	1	99.57
20	1	100.11
20	1	100.95
20	1	101
20	1	101.08
20	1	101.63
20	1	101.82
20	1	102.32
20	1	103.07
20	1	103.27
20	1	103.44
20	1	103.82
20	1	104
20	1	104.03
20	1	104.03
20	1	104.37
20	1	104.49
20	1	104.73
20	1	104.9
20	1	106.32
20	1	106.35
20	1	106.38
20	1	106.43
20	1	106.46
20	1	107.1
20	1	107.19
20	1	107.23
20	1	107.59
20	1	107.8
20	1	107.98
20	1	108

20	1	108.04
20	1	108.12
20	1	108.27
20	1	108.5
20	1	108.56
20	1	109.16
20	1	109.49
20	1	110.01
20	1	110.17
20	1	110.3
20	1	111.94
20	1	112.04
20	1	112.31
20	1	113.34
20	1	113.61
20	1	114.05
20	1	114.1
20	1	114.31
20	1	114.35
20	1	114.43
20	1	114.49
20	1	114.62
20	1	114.73
20	1	114.83
20	1	114.96
20	1	115.05
20	1	115.11
20	1	115.12
20	1	115.45
20	1	115.48
20	1	115.52
20	1	116.37
20	1	116.47
20	1	116.54
20	1	116.85
20	1	116.94
20	1	118.1
20	1	119.84
20	1	120.39
20	1	120.95
20	1	121.2
20	1	122.6
20	1	123.67
20	1	124.66
20	1	125.27
20	1	125.65
20	1	128.09

20	1	130.59
20	1	182.47

Scots pine measurement

anvil	prob.predation	length	type apophyses
1	1	49.15	1
1	1	49.04	1
1	1	46.48	1
1	1	38.53	1
1	1	40	1
1	1	50.17	2
1	1	52.38	1
1	1	47.63	2
1	1	46.74	2
1	1	40.08	2
1	1	42.04	2
1	1	49.16	1
1	1	40.96	1
1	1	39	1
1	1	37	2
1	1	35.92	1
1	1	47.64	1
1	1	38.08	1
1	1	35	1
1	1	36	2
1	1	38	2
1	1	41.1	2
1	1	39	1
1	1	35.06	1
1	1	40.93	1
1	1	40	1
1	1	45.15	1
1	1	46.45	1
1	1	45.23	2
1	1	43.32	1
1	1	46.09	1
1	1	47.03	1
1	1	43.26	2
1	1	51.03	2
1	1	45.36	1
1	1	48.38	1
1	1	47.08	1
1	1	45.47	1
1	1	38.93	1
1	1	38.22	2

1	1	46.94	1
1	1	35.19	1
1	1	36	1
1	1	37	1
1	1	48.06	1
1	1	44.89	1
1	1	47.1	2
1	1	49.33	1
1	1	45.56	1
1	1	53.58	1
1	1	37	1
1	1	47.33	1
1	1	52.97	1
1	1	36	1
1	1	43.31	1
1	1	47.76	1
1	1	51.25	1
1	1	49.81	2
1	1	43.31	2
1	1	44.47	2
1	1	42.15	2
1	1	34	2
1	1	42.85	2
1	1	47.98	1
1	1	32	1
1	1	51.04	1
1	1	42.99	1
1	1	41.84	1
1	1	38	1
1	1	47.85	1
1	1	31.48	1
1	1	38.21	2
1	1	43.24	1
1	1	44.72	1
1	1	42.25	1
1	1	38	2
1	1	44.31	1
1	1	48.22	1
1	1	46.57	1
1	1	46.58	2
1	1	41.99	1
1	1	43.3	2
1	1	40	1
1	1	41.44	1
1	1	48.7	1
1	1	38.39	1
1	1	40.42	1

1	1	35	2
1	1	43.09	1
1	1	40.15	1
1	1	37.93	1
1	1	46.09	1
1	1	44.79	2
1	0	45.88	1
1	0	45.68	2
1	0	40.74	1
1	0	42.31	1
1	0	42.27	2
1	0	44.17	1
1	0	36.43	2
1	0	37	1
1	0	38.78	1
1	0	42.82	2
1	0	39	1
1	0	38.82	2
1	0	47.55	2
1	0	41.03	2
1	0	41.86	2
1	0	51.12	2
1	0	43.51	2
1	0	50.21	2
1	0	36.72	1
1	0	42.44	2
1	0	40.98	1
1	0	40	1
1	0	48.71	2
1	0	44.69	2
1	0	47.48	2
1	0	44.54	2
1	0	47.65	2
1	0	37.19	2
1	0	49.38	2
1	0	46.13	2
1	0	49.74	1
1	0	46.82	2
1	0	38.94	2
1	0	44.4	2
1	0	39.47	2
1	0	48.37	2
1	0	49.44	2
1	0	39	2
1	0	46.38	1
1	0	42.82	1
1	0	40.74	2

1	0	42.54	2
1	0	48.4	2
1	0	45.01	1
1	0	44.49	2
1	0	47.01	2
1	0	42.32	2
1	0	45.13	2
1	0	47.2	2
1	0	39.75	2
1	0	46.74	2
1	0	46.86	2
1	0	44.97	2
1	0	46.36	2
1	0	44.65	2
1	0	52.33	2
1	0	38	2
1	0	37.14	1
1	0	48.41	1
1	0	42.53	2
1	0	41.5	2
1	0	39.21	1
1	0	40	2
1	0	44.56	2
1	0	44.75	2
1	0	44.42	2
1	0	44.7	2
1	0	37	1
1	0	45.22	2
1	0	41.67	2
1	0	38	2
1	0	44.92	2
1	0	44.98	2
1	0	43.4	2
1	0	39.65	2
1	0	35	2
1	0	48.4	2
1	0	40.19	1
1	0	42.07	1
1	0	40.14	2
1	0	44.02	2
1	0	42.49	2
1	0	41.06	1
1	0	43.78	2
1	0	42.45	1
1	0	35	1
1	0	41.03	2
1	0	43.53	2

1	0	45.44	2
1	0	43.65	1
1	0	41.81	2
1	0	40.81	2
1	0	38.89	1
1	0	51.08	2
1	0	37	2
1	0	46.6	2
1	0	45.07	2
1	0	37.22	1
1	0	40.43	2
1	0	40.92	2
1	0	46.27	1
1	0	41.86	2
1	0	42.11	2
1	0	41.44	2
1	0	37.46	1
1	0	44.39	2
1	0	43.3	2
1	0	42.15	2
1	0	45.46	2
1	0	42.61	2
1	0	43.1	2
1	0	43.36	2
1	0	44.53	2
1	0	43.58	2
1	0	41.86	2
1	0	39.28	2
1	0	40.85	2
1	0	41.09	2
1	0	41.4	2
1	0	42.5	2
1	0	35	2
1	0	34	2
1	0	40.11	2
1	0	42.55	2
1	0	47.92	2
1	0	40.5	1
1	0	46.64	2
1	0	36.53	2
1	0	46.77	1
1	0	44.06	1
1	0	44.03	2
1	0	42.44	2
1	0	36	2
1	0	42.01	2
1	0	43.89	2

1	0	41.6	2
1	0	39.57	1
1	0	44.09	2
1	0	40	2
1	0	36	2
1	0	42.48	2
1	0	36.5	2
1	0	36	2
1	0	42.09	2
1	0	37.53	1
1	0	35	2
1	0	37.67	2
1	0	43.09	2
1	0	44.66	2
1	0	37.53	2
2	1	41.65	1
2	1	40.79	1
2	1	44.56	1
2	1	35.53	1
2	1	35	1
2	1	34.72	1
2	1	40.53	1
2	1	32.28	1
2	1	33.86	2
2	1	39.25	1
2	1	37	2
2	1	40.6	1
2	1	36.35	1
2	1	37.58	1
2	1	33	1
2	1	41.14	1
2	1	39.58	1
2	1	38.83	1
2	1	35	1
2	1	31.59	1
2	1	29.66	1
2	0	44.88	2
2	0	44.41	2
2	0	43.57	2
2	0	42.44	2
2	0	42.96	2
2	0	39.18	2
2	0	43.17	2
2	0	48.44	2
2	0	44.77	2
2	0	46.41	2
2	0	48.45	2

2	0	45.39	2
2	0	42.17	2
2	0	38.21	1
2	0	41.93	2
2	0	42.85	2
2	0	41.24	1
2	0	38	2
2	0	37.71	1
2	0	35.39	1
2	0	31.61	2
2	0	37.35	2
2	0	44.04	2
2	0	41.25	2
2	0	41.87	2
2	0	39.33	1
2	0	41.33	2
2	0	32	1
2	0	43.18	2
2	0	36.4	1
2	0	34	1
2	0	35.15	2
2	0	44.17	2
2	0	37	1
2	0	48.87	2
2	0	48.31	2
2	0	43.61	1
2	0	31.4	1
2	0	39.76	2
2	0	38.43	2
2	0	41.27	2
2	0	34	1
2	0	40.26	2
2	0	32.29	2
2	0	42.29	2
2	0	42.4	2
2	0	38	2
2	0	37.75	2
2	0	37.35	2
2	0	34.29	2
2	0	34.24	2
2	0	40	2
2	0	38	2
2	0	34.49	1
2	0	27.61	1
2	0	42.73	2
2	0	40.17	2
2	0	40	2

2	0	39.32	2
2	0	41.24	2
2	0	46.18	2
2	0	41.33	2
2	0	40.71	2
2	0	37	2
2	0	42.3	2
2	0	41.02	2
2	0	48.27	2
2	0	38	2
2	0	38	2
2	0	41.17	1
2	0	41.5	2
2	0	46.73	2
2	0	41.61	2
2	0	41.78	2
2	0	38.6	2
2	0	45.46	2
2	0	38.1	2
2	0	38.99	1
2	0	45.39	2
2	0	44.11	2
2	0	39	2
2	0	37.6	2
2	0	46	2
2	0	40.24	1
2	0	35	2
2	0	41.29	1
2	0	36.71	1
2	0	34.5	2
2	0	42.78	2
2	0	41.66	2
2	0	36.26	2
2	0	46.27	2
2	0	36	1
2	0	37.83	2
2	0	41.5	2
2	0	40.49	2
2	0	39.54	2
2	0	36.76	2
2	0	40	2
2	0	33	1
2	0	44.62	2
2	0	37	1
2	0	42.46	2
2	0	44.01	2
2	0	40.64	2

2	0	37	2
2	0	36.03	2
2	0	43.62	2
2	0	38.58	1
2	0	39.83	2
2	0	41.48	2
2	0	45.51	2
2	0	40	2
2	0	42.29	2
2	0	38	2
2	0	37	2
2	0	47.41	2
2	0	35.4	2
2	0	38.75	2
2	0	51.93	2
2	0	41.46	2
2	0	47.56	1
2	0	44.61	2
2	0	37	2
2	0	42.66	2
2	0	37	1
2	0	32.36	1
2	0	45.22	2
2	0	38	2
2	0	45.73	2
2	0	40.71	2
2	0	46.14	2
2	0	41.65	1
2	0	34.99	2
2	0	40	2
2	0	44.06	2
2	0	33	1
2	0	37.78	2
2	0	42.27	2
2	0	41.07	2
2	0	42.28	2
2	0	44.06	2
2	0	36.61	2
2	0	43.15	2
2	0	41.15	2
2	0	32	1
2	0	37	2
2	0	36	2
2	0	34.53	1
2	0	39.22	2
2	0	36.1	2
2	0	41.71	1

2	0	31.62	2
2	0	36	1
2	0	44.82	2
2	0	40	2
2	0	35.96	1
2	0	41.8	2
2	0	43.06	2
2	0	35	1
2	0	43.94	2
2	0	37.43	1
2	0	44.96	2
2	0	36.15	2
2	0	37.42	2
2	0	38.94	2
2	0	43.96	2
2	0	36.15	2
2	0	43.12	2
2	0	39	2
2	0	48.73	2
2	0	39.56	2
2	0	46.19	2
2	0	43.98	2
2	0	49.32	2
2	0	49.21	2
2	0	46.33	2
2	0	44.6	2
2	0	41.36	2
2	0	50.64	2
2	0	41.67	2
2	0	37.07	2
2	0	42.74	2
2	0	53.37	2
2	0	45.53	2
2	0	49.81	2
2	0	45.56	2
2	0	45.86	2
2	0	45.48	2
2	0	45.01	2
2	0	46	2
2	0	39	2
2	0	42.83	2
2	0	48.05	2
2	0	40.33	2
2	0	49.94	2
2	0	48.11	2
2	0	45.19	2
2	0	52.18	2

2	0	48.29	2
2	0	41.6	2
2	0	49.68	2
2	0	40.47	2
2	0	45.79	2
2	0	39.46	2
2	0	45.23	2
2	0	40.01	2
2	0	34.39	2
2	0	45.53	2
2	0	42.59	2
2	0	39.53	2
2	0	44.37	2
2	0	40.61	2
2	0	48.98	2
2	0	49.22	2
2	0	43.97	2
2	0	41.16	2
2	0	46.82	2
2	0	42.81	2
2	0	42.53	2
2	0	45.74	2
2	0	47.19	2
2	0	48.75	2
2	0	43.74	2
2	0	33.65	2
2	0	45.42	2
2	0	37.26	2
2	0	49.03	2
2	0	36	2
2	0	47.93	2
2	0	49.33	2
2	0	42.52	2
2	0	40.74	2
2	0	43.32	2
2	0	41.95	2
2	0	47.3	2
2	0	41.4	2
2	0	42.34	2
2	0	41.92	2
2	0	44.16	2
2	0	47.68	2
2	0	45.11	2
2	0	44.31	2
2	0	41.79	2
2	0	32	2
2	0	40.96	2

2	0	42.51	2
2	0	42.44	2
2	0	47.38	2
2	0	43.4	2
2	0	39	2
2	0	43.63	2
2	0	37.15	2
2	0	35	2
2	0	40.39	2
2	0	41.97	2
2	0	44.31	2
2	0	40.47	2
2	0	41.97	2
2	0	46.16	2
2	0	43.86	2
2	0	46.06	2
2	0	50.25	2
2	0	44.56	2
2	0	40.56	2
2	0	50.27	2
2	0	45.75	2
2	0	39.42	2
2	0	47.39	2
2	0	38.76	2
2	0	41.68	2
2	0	31.87	2
2	0	46.89	2
2	0	32.56	2
2	0	50.76	2
2	0	43.05	2
2	0	41.31	2
2	0	47.84	2
2	0	45.16	2
2	0	39.07	2
2	0	46.35	2
2	0	34.74	2
2	0	44.09	2
2	0	43.61	2
2	0	44.89	2
2	0	47.3	2
2	0	39	2
2	0	43.35	2
2	0	40.53	2
2	0	46.3	2
2	0	54.82	2
2	0	48.28	2
2	0	39	2

2	0	37.93	2
2	0	41.94	2
2	0	36.32	2
2	0	42.58	2
2	0	51.24	2
2	0	48.2	2
2	0	36.14	2
3	1	35.5	1
3	1	41.39	1
3	1	41.43	2
3	1	38	2
3	1	37.75	2
3	1	48.77	1
3	1	41.04	1
3	1	41.01	1
3	1	40.24	1
3	1	54.88	1
3	1	34.86	2
3	1	35.17	1
3	0	45.25	2
3	0	44.1	2
3	0	47	2
3	0	39	2
3	0	43.48	2
3	0	47.09	2
3	0	42.61	2
3	0	42.59	2
3	0	46.23	2
3	0	39.9	2
3	0	39.81	2
3	0	47.34	2
3	0	41.68	2
3	0	41.27	2
3	0	41.14	2
3	0	36.17	2
3	0	39.11	2
3	0	48.58	2
3	0	46.57	2
3	0	46.97	2
3	0	47.3	2
3	0	34.24	2
3	0	38.99	2
3	0	45.94	2
3	0	35	2
3	0	37.89	2
3	0	35.51	2
3	0	35	2

3	0	38.36	2
3	0	46.61	2
3	0	46.8	2
3	0	37	2
3	0	34.43	2
3	0	35	2
3	0	39	2
3	0	37	2
3	0	33	2
3	0	37.47	2
3	0	37.51	2
3	0	37.93	2
3	0	33.83	2
3	0	42.58	2
3	0	43.7	2
3	0	43.5	2
3	0	43.22	2
3	0	39.89	2
3	0	42.03	2
3	0	41.57	2
3	0	32.15	2
3	0	38.39	2
3	0	41.15	2
3	0	39	2
3	0	49.29	2
3	0	49.17	2
3	0	50.37	2
3	0	36.53	2
3	0	36.25	1
3	0	37.33	2
3	0	35.89	2
3	0	44.04	2
3	0	51.58	2
3	0	39.58	2
3	0	40.22	2
3	0	37.92	2
3	0	46.94	2
3	0	37.15	2
3	0	38.54	2
3	0	45.8	2
3	0	50.05	2
3	0	36	2
3	0	36.54	2
3	0	32	2
3	0	40	1
3	0	35.53	2
3	0	37	2

3	0	45.94	2
3	0	38.9	2
3	0	34.78	2
3	0	40	2
3	0	34.93	2
3	0	39.21	2
3	0	49.6	2
3	0	41.02	2
3	0	37.42	2
3	0	49.09	2
3	0	38.57	2
3	0	33.72	2
3	0	34	2
3	0	41.49	2
3	0	44.93	2
3	0	32.43	2
3	0	33.44	2
3	0	34	2
3	0	30.7	2
3	0	43.07	2
3	0	35.31	2
3	0	41.76	2
3	0	42.19	2
3	0	37	2
3	0	34.35	2
3	0	47.02	2
3	0	33.61	2
3	0	30.25	2
3	0	38.4	2
3	0	30.93	2
3	0	37.72	2
3	0	38	2
3	0	34	2
3	0	37	2
3	0	37	2
3	0	45.54	2
3	0	37.93	2
3	0	37	2
3	0	33.24	2
3	0	42.54	2
3	0	48.77	2
3	0	36.42	2
3	0	36	2
3	0	47.18	2
3	0	40	2
3	0	39	2
3	0	44.57	2

3	0	35.71	2
3	0	34	2
3	0	50	2
3	0	37	2
3	0	40.08	2
3	0	39.68	2
3	0	41.35	2
3	0	39.25	2
3	0	38	2
3	0	38.17	2
3	0	38.79	2
3	0	40	2
3	0	46.09	2
3	0	45.42	2
3	0	40.51	2
3	0	41.03	2
3	0	37.68	2
3	0	38.86	2
3	0	40.71	2
3	0	38	2
3	0	44.28	2
3	0	35.69	2
3	0	42.7	2
3	0	42.86	2
3	0	37.51	2
3	0	36.5	1
3	0	39	2
3	0	41.59	2
3	0	47.94	2
3	0	37	2
3	0	35	2
3	0	40.26	2
3	0	53.96	2
3	0	40	2
3	0	50.31	2
3	0	38.78	2
3	0	43.37	2
3	0	35	2
3	0	47.19	1
3	0	41.81	2
3	0	43.98	2
3	0	38.74	2
3	0	41.98	2
3	0	40.51	2
3	0	50.2	2
3	0	44.8	2
3	0	44.1	2

3	0	53.81	2
3	0	39.56	2
3	0	32.76	2
3	0	38	2
3	0	38	2
3	0	42.24	2
3	0	43.13	2
3	0	36.89	2
3	0	40	2
3	0	38.54	2
3	0	38.96	2
3	0	38.43	2
3	0	30.96	2
3	0	47.5	2
3	0	38	2
3	0	45.77	2
3	0	39	2
3	0	45.99	2
3	0	36.36	2
3	0	36	2
3	0	47.93	2
3	0	44.86	2
3	0	35.46	2
3	0	42.21	2
3	0	39.42	2
3	0	37.89	2
3	0	44.86	2
3	0	33.54	2
3	0	55.88	2
3	0	46.76	2
3	0	41.52	2
3	0	44.22	2
3	0	39.78	2
3	0	32.82	2
3	0	50.98	2
3	0	33	2
3	0	42.1	2
3	0	53.59	2
3	0	38	2
3	0	29.28	2
3	0	31.95	2
3	0	42.1	2
3	0	44.76	2
3	0	40	2
3	0	47.74	2
3	0	48.89	2
3	0	49.52	2

3	0	42.68	2
3	0	41.17	2
3	0	30.66	2
3	0	42.69	2
3	0	41.22	2
3	0	34	2
3	0	44.5	2
3	0	35.56	2
3	0	40.35	2
3	0	36	2
3	0	33.61	2
3	0	41.73	2
3	0	36.64	2
3	0	34.81	2
3	0	35	2
3	0	31.45	2
3	0	37.49	2
3	0	36.39	2
3	0	38.21	2
3	0	50.78	2
3	0	41.65	2
3	0	35	2
3	0	36	2
3	0	42.88	2
3	0	38.53	2
3	0	33.15	2
3	0	38.25	2
3	0	34.28	2
3	0	42.01	2
3	0	31.35	2
3	0	45.24	2
3	0	37.81	2
3	0	36	2
3	0	33	2
3	0	44.36	2
3	0	35	2
3	0	34	2
3	0	37.72	2
3	0	36.28	2
3	0	35.54	2
3	0	39	2
3	0	32.4	2
3	0	27.37	2
3	0	32	2
4	1	40.49	2
4	1	47.17	1
4	1	30.11	1

4	1	43.33	1
4	1	46.69	1
4	1	40	1
4	1	51.19	1
4	1	38.83	1
4	1	44.92	1
4	1	46.16	1
4	1	36.42	1
4	1	44.58	1
4	1	46.03	1
4	1	35.42	2
4	1	43.78	2
4	1	45.14	1
4	1	40.58	1
4	1	47.62	1
4	1	38	1
4	1	41.92	1
4	1	47.69	1
4	1	40.29	1
4	1	38.43	2
4	1	46.01	1
4	1	37.57	1
4	1	43.76	1
4	1	45.28	2
4	1	33.85	2
4	1	33	2
4	1	40.96	1
4	1	41.84	1
4	1	32.57	1
4	1	41.78	2
4	1	44.77	1
4	1	34.21	1
4	1	39.58	1
4	1	37.39	1
4	1	34.94	1
4	1	42.57	1
4	1	42.06	1
4	1	41.86	1
4	1	36	1
4	1	44.03	1
4	1	46.51	1
4	1	40.97	2
4	1	34	2
4	1	37.1	2
4	1	33.51	2
4	1	37	2
4	1	36	2

4	1	37	2
4	1	39.21	1
4	1	39	1
4	1	36.47	1
4	1	35.31	1
4	1	35.36	1
4	1	37	1
4	1	38.64	1
4	1	41.06	1
4	1	39.53	2
4	1	37	2
4	1	41.46	1
4	1	36.83	1
4	1	40.9	1
4	1	38	2
4	1	35	2
4	1	39.89	2
4	1	38.51	1
4	1	36.6	2
4	1	37.42	1
4	1	33.36	2
4	1	39	2
4	1	38.61	1
4	1	36.11	1
4	1	40.01	1
4	1	34.69	1
4	1	37	1
4	1	39	1
4	1	34.36	1
4	1	35.89	2
4	1	34	1
4	1	39.96	2
4	1	37	2
4	1	42.48	2
4	1	35	1
4	1	38.83	1
4	1	40	1
4	1	37	1
4	1	33	2
4	1	39.49	2
4	1	34.53	2
4	1	37.82	2
4	1	40.58	1
4	1	45.51	2
4	1	39	1
4	1	36.28	2
4	1	32.92	1

4	1	34.47	1
4	1	38.29	1
4	1	42.88	2
4	1	40.11	1
4	1	38	2
4	1	35	2
4	1	39	1
4	1	34.67	1
4	1	33.64	1
4	1	34	2
4	1	29.79	2
4	1	40	1
4	1	41.13	1
4	1	26.55	2
4	1	36.08	2
4	1	35.29	2
4	1	39.4	2
4	1	30.04	1
4	1	40.89	1
4	1	37.4	1
4	1	38	2
4	1	36.31	1
4	1	39.61	2
4	1	35	2
4	1	33.04	1
4	1	33	2
4	1	37.4	1
4	1	35.42	2
4	1	45.91	2
4	1	39	2
4	1	39.15	2
4	1	38	1
4	1	35.72	2
4	1	37.47	1
4	1	37.5	1
4	1	38.08	1
4	1	41.44	1
4	1	34.54	2
4	1	37.28	1
4	1	34	2
4	1	37.5	2
4	1	34.96	2
4	1	35.65	1
4	1	41.16	2
4	1	36.99	1
4	1	38.47	1
4	1	39	1

4	1	43.54	2
4	1	36.72	1
4	1	34.61	1
4	1	32.69	1
4	1	50.14	2
4	1	35	1
4	1	41.34	2
4	1	33.18	1
4	1	49.29	2
4	1	40	2
4	1	44.55	2
4	1	33	1
4	1	40.57	2
4	1	35	1
4	1	39.35	1
4	1	41.95	2
4	1	34.47	1
4	1	31.92	1
4	1	41.51	2
4	1	35.71	1
4	1	33.17	1
4	1	38.42	2
4	1	34	1
4	1	36.25	1
4	1	32.21	1
4	1	35.44	1
4	1	33.97	1
4	1	34.9	1
4	1	38.43	1
4	1	49.3	2
4	1	42.45	2
4	1	37	1
4	1	35.64	1
4	1	38.25	1
4	1	38.49	1
4	1	39.57	1
4	1	41.99	2
4	1	34.56	1
4	1	40.29	2
4	1	39.89	1
4	1	41.39	2
4	1	32	1
4	1	42.32	1
4	1	33.44	1
4	1	38	1
4	1	41.62	2
4	1	34.69	1

4	1	37.08	2
4	1	37	2
4	1	31.23	2
4	1	36.74	1
4	1	39.32	2
4	1	38.25	2
4	1	41.21	2
4	1	40	2
4	1	34	2
4	1	36	1
4	1	41.79	2
4	1	42.73	2
4	1	40	1
4	1	35.28	1
4	1	44.34	1
4	1	33.49	1
4	1	38.72	1
4	1	42.08	1
4	1	37	1
4	1	39.33	1
4	1	42.77	2
4	1	40.56	1
4	1	41.76	2
4	1	37.67	1
4	1	40.21	1
4	1	39	2
4	1	40.97	2
4	1	38.33	2
4	1	36.5	1
4	1	43.27	1
4	1	32	2
4	1	41.3	2
4	1	46.3	2
4	1	33.54	2
4	1	40.97	1
4	1	39.04	1
4	1	39.36	2
4	1	35.97	1
4	1	41.42	1
4	1	33.32	2
4	1	46.3	1
4	1	29.57	1
4	1	40	1
4	1	37.42	2
4	1	37	1
4	1	43.11	1
4	1	36.35	1

4	1	35	1
4	1	37.81	1
4	1	33	2
4	1	40	2
4	0	47.58	2
4	0	42.82	2
4	0	47.78	1
4	0	39.68	1
4	0	45.39	2
4	0	46.29	1
4	0	44.54	2
4	0	40	1
4	0	40.96	2
4	0	48.17	2
4	0	43.41	1
4	0	45.15	1
4	0	48.15	2
4	0	48.68	2
4	0	45.89	1
4	0	45.59	2
4	0	51.35	2
4	0	41.17	2
4	0	42.76	1
4	0	46.45	2
4	0	52.73	2
4	0	47.43	2
4	0	46.92	2
4	0	41.86	1
4	0	32.32	2
4	0	38.29	1
4	0	47	2
4	0	39.56	1
4	0	39.53	1
4	0	44.55	2
4	0	42.37	1
4	0	51.27	2
4	0	46.74	2
4	0	37.03	1
4	0	44.71	1
4	0	42.54	2
4	0	47.72	2
4	0	47.35	2
4	0	37	1
4	0	51.73	1
4	0	44.07	2
4	0	43.66	1
4	0	44.49	2

4	0	46.77	2
4	0	46.34	2
4	0	40	2
4	0	48.45	2
4	0	38	2
4	0	41.36	1
4	0	48.75	2
4	0	46.53	2
4	0	43.29	1
4	0	44.16	2
4	0	41.54	2
4	0	49.1	2
4	0	39.54	1
4	0	48.81	2
4	0	42.52	2
4	0	42.77	2
4	0	44.4	2
4	0	47.8	1
4	0	43.58	1
4	0	37.17	1
4	0	50.07	2
4	0	49.63	2
4	0	48.58	2
4	0	46.36	2
4	0	43.5	1
4	0	46.93	1
4	0	48.88	2
4	0	40	1
4	0	45.25	1
4	0	42.64	2
4	0	41.36	1
4	0	39	1
4	0	46.56	2
4	0	43.26	1
4	0	46.24	1
4	0	42.94	2
4	0	40.79	1
4	0	43.53	1
4	0	47.8	2
4	0	47.09	2
4	0	44.96	2
4	0	44.5	2
4	0	45.79	1
4	0	40.81	2
4	0	47.99	1
4	0	38.58	1
4	0	44.25	2

4	0	42.18	2
4	0	48.65	2
4	0	44.65	2
4	0	44.99	2
4	0	40	1
4	0	49.46	2
4	0	45.06	2
4	0	43.21	2
4	0	50.94	2
4	0	49.01	2
4	0	36.99	1
4	0	40.85	1
4	0	48.29	2
4	0	46.83	2
4	0	41.27	2
4	0	38	2
4	0	50.26	2
4	0	45.02	2
4	0	43.38	2
4	0	43.53	2
4	0	35	1
4	0	43.12	2
4	0	44.64	2
4	0	43.08	2
4	0	50	2
4	0	46.19	2
4	0	49.93	2
4	0	42.82	2
4	0	43.28	2
4	0	44.13	2
4	0	48.08	2
4	0	41.58	2
4	0	45.33	2
4	0	37	1
4	0	35.49	1
4	0	40.96	2
4	0	43.23	1
4	0	51.7	2
4	0	38.5	2
4	0	41.66	2
4	0	35	2
4	0	27.1	1
4	0	46.63	2
4	0	38	1
4	0	36.49	1
4	0	36	2
4	0	48.01	2

4	0	41.46	2
4	0	40.9	2
4	0	42.27	2
4	0	39.08	2
4	0	37.54	1
4	0	44.67	2
4	0	51.82	2
4	0	45.99	2
4	0	49.17	2
4	0	48.29	2
4	0	43.12	2
4	0	50.3	2
4	0	53.04	2
4	0	42.19	1
4	0	39	1
4	0	39.4	1
4	0	35	1
4	0	38.54	1
4	0	39	2
4	0	41.85	2
4	0	36.49	1
4	0	49.72	2
4	0	38	1
4	0	43.26	2
4	0	43.62	2
4	0	41.8	2
4	0	41.47	2
4	0	41.96	2
4	0	41.33	2
4	0	40	2
4	0	41.21	2
4	0	43.36	1
4	0	37.18	1
4	0	42.77	2
4	0	41.38	2
4	0	55.18	2
4	0	39.28	2
4	0	46.01	2
4	0	42.78	2
4	0	45.57	2
4	0	40.35	1
4	0	39	1
4	0	45.83	2
4	0	44.11	2
4	0	50.46	2
4	0	46.91	2
4	0	44.38	2

4	0	45.2	2
4	0	42.26	2
4	0	48.43	2
4	0	43.16	2
4	0	37.82	2
4	0	40	2
4	0	44.41	2
4	0	45.92	2
4	0	44.53	2
4	0	43.34	2
4	0	47.76	2
4	0	42.76	2
4	0	40	1
4	0	41.15	2
4	0	40.1	2
4	0	48.11	2
4	0	46.68	1
4	0	44.28	2
4	0	48.52	2
4	0	48.23	2
4	0	43.7	2
4	0	42.56	2
4	0	52.58	2
4	0	42.34	2
4	0	42.33	2
4	0	38.58	2
4	0	44.42	2
4	0	42.26	2
4	0	43.1	2
4	0	38.54	1
4	0	37.74	2
4	0	39.47	2
4	0	42.51	2
4	0	40.36	1
4	0	43.25	2
4	0	50.32	2
4	0	39	1
4	0	36	1
4	0	43.78	2
4	0	33.71	1
8	1	46.22	1
8	1	51.15	1
8	1	44.4	1
8	1	35.18	1
8	1	51.74	1
8	1	39.86	1
8	1	47.87	2

8	1	49.3	1
8	1	33.99	2
8	1	33.65	1
8	1	37	1
8	1	36.24	2
8	1	38.01	1
8	1	46.79	2
8	1	42.82	1
8	1	39.29	1
8	1	44.04	2
8	1	40.1	1
8	1	37	1
8	1	38.61	1
8	1	33.4	1
8	1	32	1
8	1	44.4	1
8	1	36	1
8	1	31.92	1
8	1	41.2	1
8	1	37	1
8	1	45.2	1
8	1	41.05	1
8	1	42.98	1
8	1	41.32	1
8	1	43.62	1
8	1	38.93	1
8	1	48.11	1
8	1	36.86	1
8	1	31.99	1
8	1	37	1
8	1	42.86	2
8	1	35.54	1
8	1	40	1
8	1	45.46	1
8	1	40.61	1
8	1	40	1
8	1	45.04	2
8	1	33.58	1
8	1	37.49	1
8	1	42.22	1
8	1	42.35	2
8	1	35.36	1
8	1	37.08	1
8	1	39.58	1
8	0	49	1
8	0	37.81	1
8	0	38.78	1

8	0	43.15	1
8	0	42.39	1
8	0	37.14	2
8	0	37.83	1
8	0	45.61	2
8	0	42.64	2
8	0	34.47	1
8	0	40.32	1
8	0	39.32	1
8	0	44.41	2
8	0	41.35	1
8	0	41.38	1
8	0	37	1
8	0	40.32	1
8	0	38.06	1
8	0	39.43	2
8	0	39.01	2
8	0	38.71	2
8	0	37.4	2
8	0	34.24	1
8	0	44.17	2
8	0	38	2
8	0	34.57	1
8	0	40.89	1
8	0	39.22	2
8	0	32.97	1
8	0	32	1
8	0	47.75	2
8	0	44.45	2
8	0	45.13	2
8	0	32	1
8	0	43.68	1
8	0	37.78	2
8	0	36.17	2
8	0	40	1
8	0	39.92	2
8	0	42.27	2
8	0	35.29	1
8	0	43.35	1
8	0	40.53	1
8	0	32.75	1
8	0	46.75	1
8	0	30.74	1
8	0	35.65	1
8	0	37	1
8	0	40	2
8	0	33.81	1

8	0	37.53	1
8	0	44.94	1
8	0	51.17	2
8	0	34.49	1
8	0	32.76	1
8	0	41.95	1
8	0	32.58	1
8	0	34.17	1
8	0	43.36	1
8	0	42.01	2
8	0	33.97	1
8	0	38	2
8	0	41.86	1
8	0	40.18	1
8	0	52.26	2
8	0	32	2
8	0	40	1
8	0	31.53	1
8	0	37.21	2
8	0	40.1	2
8	0	33	2
8	0	38	2
8	0	39	1
8	0	44.89	2
8	0	43.14	2
8	0	37.72	2
8	0	31.93	2
8	0	42.18	2
8	0	34.46	2
8	0	42.43	2
8	0	36	2
8	0	39	2
8	0	48.68	2
8	0	37.4	1
8	0	41.11	2
8	0	32	1
8	0	36.03	2
8	0	40.81	1
8	0	39.78	1
8	0	38.72	1
8	0	39.58	2
8	0	38	2
8	0	42.52	1
8	0	39.9	2
8	0	32	1
8	0	39.64	1
8	0	44.82	2

8	0	33	1
8	0	31.87	2
8	0	38.07	1
8	0	37	1
8	0	35	2
8	0	38	2
8	0	41.52	2
8	0	41.31	1
8	0	37.99	1
8	0	38	1
8	0	36.35	1
8	0	40	2
8	0	31.57	1
8	0	37	1
8	0	40	2
8	0	42.67	2
8	0	41.92	2
8	0	36.06	2
8	0	40.67	2
8	0	46.92	2
8	0	40	1
8	0	43.38	1
8	0	39.67	2
8	0	36	2
8	0	39	1
8	0	39.99	2
8	0	36	2
8	0	36.6	1
8	0	32	1
8	0	36.19	2
8	0	34.07	2
8	0	33.33	2
8	0	43.23	2
8	0	40	1
8	0	38.06	2
8	0	31.1	1
8	0	34.76	1
8	0	36.06	2
8	0	42.2	1
8	0	36	1
8	0	39.07	1
8	0	37	1
8	0	40.49	1
8	0	41.75	2
8	0	37	2
8	0	43	1
8	0	37	1

8	0	38.83	1
8	0	36.04	1
8	0	31.79	1
8	0	38.65	2
8	0	34.28	1
8	0	32.58	2
8	0	41.95	2
8	0	35.81	2
8	0	38	1
8	0	35.04	2
8	0	36.49	2
8	0	43.86	2
8	0	36	1
8	0	37	2
8	0	36	2
8	0	45.45	2
8	0	30.74	1
8	0	38.11	2
8	0	44.86	2
8	0	25.76	1
8	0	32.19	1
8	0	30.61	1
8	0	38	1
8	0	38	1
8	0	40.68	1
8	0	46.05	2
8	0	30.2	1
8	0	35.04	1
8	0	44.27	2
8	0	35	1
8	0	38.54	2
8	0	34	1
8	0	38.1	1
8	0	36	2
8	0	43.97	2
8	0	36.08	2
8	0	37.33	2
8	0	40.82	1
8	0	42.21	2
8	0	35.22	1
8	0	37.46	1
8	0	31.86	2
8	0	45.77	2
8	0	37.49	1
8	0	34.97	1
8	0	32.21	1
8	0	50.52	2

8	0	48.09	2
8	0	43.97	2
8	0	35.18	1
8	0	43.62	1
8	0	28.6	1
8	0	37	2
8	0	38.25	2
8	0	29.66	1
8	0	39	2
8	0	41.39	2
8	0	38	1
8	0	44.26	2
8	0	36.97	2
8	0	40.17	2
8	0	37	1
8	0	34.65	1
8	0	35.18	1
8	0	32	2
8	0	30.8	2
8	0	35	1
8	0	40.43	2
8	0	42.08	2
8	0	36.64	1
8	0	38	2
8	0	41.96	2
8	0	34.85	2
8	0	38	2
8	0	34.76	1
8	0	34	1
8	0	44.2	2
8	0	40.19	2
8	0	44.38	2
8	0	43.12	2
8	0	38.94	2
8	0	44.98	2
8	0	43.11	2
8	0	34	1
8	0	37.58	2
8	0	31.82	2
8	0	33	2
8	0	42.04	1
8	0	30	1
8	0	36	2
8	0	35	1
8	0	35	1
8	0	45.18	2
8	0	37	1

8	0	38	2
8	0	41.06	2
8	0	41.53	2
8	0	37	2
8	0	41.51	2
8	0	38	2
8	0	28.42	1
8	0	47.98	2
8	0	37.54	2
8	0	38	1
8	0	40	1
8	0	43.43	2
8	0	34	1
8	0	48.64	2
8	0	39.17	2
8	0	31.15	1
8	0	40.25	2
8	0	40	2
8	0	36.28	2
8	0	42.48	2
8	0	42.86	2
8	0	32	1
8	0	39.36	2
8	0	44.15	1
8	0	40	2
8	0	50.8	2
8	0	38.99	2
8	0	39.53	1
8	0	46.84	2
8	0	37	1
8	0	37.65	1
8	0	33	1
8	0	34.54	2
8	0	35	2
8	0	41.45	1
8	0	40	2
8	0	39.94	2
8	0	33.82	1
8	0	33.67	1
8	0	39.49	2
8	0	34.76	2
8	0	37.83	2
8	0	40.42	2
8	0	44.7	2
8	0	33	1
8	0	45.11	2
8	0	31.41	2

8	0	41.14	2
8	0	45.14	2
8	0	37.15	2
8	0	34	2
8	0	33.69	1
8	0	35.89	2
8	0	36.89	2
8	0	44.21	2
8	0	32	1
8	0	36	2
8	0	32.42	2
8	0	43.39	2
8	0	40.57	2
8	0	41.59	2
8	0	35.96	2
8	0	28.26	1
8	0	43.2	2
8	0	35.28	2
8	0	30.89	1
8	0	32.11	1
8	0	31.33	1
8	0	33	2
8	0	32.68	2
8	0	36.74	2
8	0	44.62	1
8	0	34.72	2
8	0	36.9	2
8	0	41.74	2
8	0	37.26	2
8	0	40.6	1
8	0	33	2
8	0	36.26	1
8	0	44.34	1
8	0	38.22	2
8	0	35	1
8	0	42.68	2
8	0	45.07	2
8	0	43.86	2
8	0	32	1
8	0	38.79	2
8	0	44.06	1
8	0	35	1
8	0	40.04	2
8	0	34.54	1
8	0	35.17	1
8	0	32.26	2
8	0	39.97	1

8	0	41.39	2
8	0	43.21	2
8	0	36.65	2
8	0	38	2
8	0	33.97	1
8	0	34.93	1
8	0	37	1
8	0	34	2
8	0	34.26	1
8	0	35.11	1
8	0	31.47	1
8	0	39.97	1
8	0	29.36	2
8	0	35.58	2
8	0	35	2
8	0	44.2	2
8	0	38.67	1
8	0	44.54	1
8	0	43.46	2
8	0	35.47	1
8	0	35.56	1
8	0	32.67	2
8	0	31.22	1
8	0	32.89	2
8	0	37.86	2
8	0	39.14	2
8	0	33.51	1
8	0	39.19	2
8	0	32.64	2
8	0	34.74	2
8	0	36.17	1
8	0	35.47	2
8	0	38.17	2
8	0	33.51	1
8	0	32.9	2
8	0	39	2
8	0	33	1
8	0	33	1
8	0	28.38	1
8	0	32.18	1
8	0	36.18	1
8	0	43.55	2
8	0	43.62	2
8	0	32.67	1
8	0	33.22	1
8	0	38	2
8	0	31.74	2

8	0	28.57	1
8	0	36	2
8	0	30.38	1
8	0	31.55	2
8	0	31.32	1
8	0	40.96	2
8	0	31.48	2
8	0	40	2
8	0	44	2
8	0	35.71	2
8	0	34.18	2
8	0	38	2
8	0	36.79	1
8	0	27.26	1
8	0	42.75	2
8	0	33.64	2
8	0	27.35	1
8	0	30.79	1
8	0	36.72	2
8	0	29.58	1
10	1	40.93	1
10	1	42.05	1
10	1	34	1
10	1	38	1
10	1	46.59	1
10	1	40.51	2
10	1	36	1
10	1	47.35	2
10	1	48.71	2
10	1	34.19	1
10	1	42.55	1
10	1	45.26	1
10	1	42.72	2
10	1	42.33	1
10	1	46.28	2
10	1	47.7	1
10	1	37	1
10	1	41.62	1
10	1	41.16	2
10	1	39.89	1
10	1	47.03	1
10	1	40.29	2
10	1	43.98	2
10	1	51.91	1
10	1	35.33	1
10	1	44.75	1
10	1	35.31	1

10	1	42.05	1
10	1	37	1
10	1	38	1
10	1	40	1
10	1	41.93	2
10	1	46.98	2
10	1	49.31	2
10	1	46.1	1
10	1	41.19	1
10	1	44.38	1
10	1	41.56	2
10	1	45.4	2
10	1	47.06	1
10	1	46.27	2
10	1	41.11	1
10	1	48.87	1
10	1	37.86	1
10	1	38.21	1
10	1	42.03	1
10	1	41.38	1
10	1	42.49	2
10	1	41.58	1
10	1	41.23	1
10	1	44.24	2
10	1	47.04	1
10	1	45.33	1
10	1	39	2
10	1	40.69	1
10	1	41.12	1
10	1	39.01	1
10	1	38.46	1
10	1	44	1
10	1	45.95	2
10	1	40.14	1
10	1	36.47	2
10	1	43.24	1
10	1	43.68	2
10	1	43.73	2
10	1	45.21	2
10	1	45.89	2
10	1	38.06	1
10	1	37.92	1
10	1	40	2
10	1	43.31	1
10	1	39	1
10	1	43.97	2
10	1	41.81	1

10	1	43.46	2
10	1	40	1
10	1	40.22	2
10	1	37	1
10	1	44.97	1
10	1	35.61	2
10	1	48.78	1
10	1	44.18	1
10	1	49.43	1
10	1	43.19	2
10	1	40.04	2
10	1	44.4	2
10	1	45.07	1
10	1	41.36	1
10	1	36.1	1
10	1	33.74	1
10	1	37	1
10	1	44.74	1
10	1	41.11	2
10	1	46.39	1
10	1	47.08	2
10	1	53.52	2
10	1	45.55	2
10	1	40	1
10	1	37	2
10	1	47.65	1
10	1	46.4	1
10	1	39	1
10	1	41.29	2
10	1	41.39	1
10	1	40.65	2
10	1	41.98	1
10	1	40	1
10	1	38.42	1
10	1	43.59	1
10	1	47.87	1
10	1	48.08	2
10	1	46.15	2
10	1	35.39	1
10	1	51.1	2
10	1	41.87	2
10	1	33	1
10	1	45.71	1
10	1	48.77	2
10	1	40.83	1
10	1	38.76	1
10	1	42.55	2

10	1	46.19	2
10	1	46.1	2
10	1	40.5	1
10	1	44.53	1
10	1	41.52	2
10	1	46.94	1
10	1	45.23	2
10	1	48.12	2
10	1	42.31	1
10	1	38.43	1
10	1	37	1
10	1	45.74	1
10	1	44.03	1
10	1	39	2
10	1	44.28	1
10	1	36.4	1
10	1	45.85	1
10	1	52.51	2
10	1	41.29	2
10	1	54.04	1
10	1	41.45	2
10	1	42.47	1
10	1	48.65	1
10	1	44.85	2
10	1	46.84	1
10	1	47.73	2
10	1	35.9	1
10	1	42.18	1
10	1	43.48	1
10	1	41.98	1
10	1	33	1
10	1	41.36	1
10	1	41.83	1
10	1	35.57	2
10	1	47.21	1
10	1	37.76	1
10	1	44.26	1
10	1	36.24	1
10	1	42.63	2
10	1	54.47	2
10	1	41.54	1
10	1	39.07	1
10	1	48.24	2
10	1	41.92	2
10	1	39.83	1
10	1	36.75	1
10	1	37	1

10	1	38.19	2
10	1	39.56	1
10	1	37.19	1
10	1	42.86	1
10	1	38	1
10	1	40	1
10	1	32.61	1
10	1	38.86	1
10	1	33.57	1
10	1	33.26	1
10	1	39.85	1
10	1	36.53	1
10	1	33.79	1
10	1	55.76	2
10	1	55.41	2
10	1	48.37	1
10	1	39	1
10	1	52.9	2
10	1	33	1
10	1	45.15	1
10	1	47.19	2
10	1	40	2
10	1	39.28	1
10	1	50.19	2
10	1	40.43	1
10	1	40.58	1
10	1	47.63	2
10	1	51.88	2
10	1	44.39	2
10	1	33.89	2
10	1	41.52	1
10	1	41.4	1
10	1	40.89	2
10	1	38.14	1
10	1	41.7	1
10	1	46.67	2
10	1	43.89	2
10	1	44.33	2
10	1	36.89	1
10	1	41.98	1
10	1	41.84	1
10	1	43.76	2
10	1	44.33	2
10	1	36.25	1
10	1	39	2
10	1	43.87	1
10	1	45.31	2

10	1	45.15	2
10	1	46.58	2
10	1	51.73	2
10	1	46.33	2
10	1	33.11	1
10	1	34.58	1
10	1	39.36	1
10	1	49.54	2
10	1	37.39	1
10	1	40	1
10	1	44.69	2
10	1	46.56	2
10	1	38.14	2
10	1	46.11	1
10	1	53.01	2
10	1	48.43	1
10	1	43.56	1
10	1	46.12	1
10	1	44.3	2
10	1	50.13	1
10	1	35.72	1
10	1	36	1
10	1	46.83	2
10	1	38.69	1
10	1	46.45	2
10	1	32.81	1
10	1	40.64	1
10	1	40.94	1
10	1	44.48	1
10	1	35.19	1
10	1	38.19	2
10	1	39.51	2
10	1	44.92	2
10	1	47.4	1
10	1	44.65	1
10	1	38.07	1
10	1	44.21	2
10	1	49.95	2
10	1	47.1	2
10	1	36	2
10	1	37.44	1
10	1	34.68	1
10	1	47.68	1
10	1	41.84	1
10	1	38.81	1
10	1	39.33	1
10	1	42.32	2

10	1	51.3	2
10	1	50.19	2
10	1	46.67	2
10	1	43.38	2
10	1	38	2
10	1	44.78	2
10	1	45.29	1
10	1	41.9	2
10	1	46.74	2
10	1	40	1
10	1	45.12	1
10	1	43.12	1
10	1	47	2
10	1	39.33	1
10	1	32.4	1
10	1	42.77	1
10	1	44.86	1
10	1	39.79	1
10	1	43.79	2
10	1	36	1
10	1	43.04	2
10	1	39.53	1
10	1	40.54	2
10	1	44.46	2
10	1	43.73	1
10	1	44.74	2
10	1	43.25	2
10	1	44.63	2
10	1	38	1
10	1	43.94	1
10	1	47.01	1
10	1	45.65	1
10	1	46.75	2
10	1	39.15	1
10	1	43.46	2
10	1	34.54	1
10	1	35.74	1
10	1	39.56	2
10	1	41.45	1
10	1	37	1
10	1	36	1
10	1	40	1
10	1	43.91	2
10	1	36.36	1
10	1	42.48	2
10	1	41.84	2
10	1	49.98	2

10	1	40	2
10	1	35	1
10	1	37.99	1
10	1	41.73	1
10	1	38.97	1
10	1	47.59	2
10	1	46.26	2
10	1	39	1
10	1	34.79	1
10	1	38	1
10	1	47.71	1
10	1	43.63	1
10	1	44.95	2
10	1	45.05	2
10	1	39.89	2
10	1	45.46	2
10	1	46.54	2
10	1	48.6	1
10	1	49.8	2
10	1	46.7	2
10	1	39.11	2
10	1	39	2
10	1	43.62	1
10	1	40.93	1
10	1	40	2
10	1	47.81	2
10	1	52.33	2
10	1	45.81	2
10	1	37.92	1
10	1	42.44	2
10	1	35.81	1
10	1	40	2
10	1	35.85	1
10	1	35	2
10	1	40.4	2
10	1	32.92	1
10	1	31.55	1
10	0	43.53	2
10	0	39	2
10	0	49.3	2
10	0	47.4	2
10	0	39	1
10	0	37.75	2
10	0	44.06	2
10	0	45.41	1
10	0	38.46	2
10	0	40.11	2

10	0	43.73	2
10	0	45.63	2
10	0	39.43	2
10	0	43.1	2
10	0	35.19	1
10	0	45.63	2
10	0	40	1
10	0	41.53	2
10	0	41.15	2
10	0	42.05	2
10	0	44.8	2
10	0	41.97	2
10	0	46.34	2
10	0	42.03	2
10	0	45.96	1
10	0	52.15	2
10	0	45.22	2
10	0	49.05	2
10	0	44.44	1
10	0	50.17	2
10	0	37.67	2
10	0	42.16	1
10	0	45.66	2
10	0	40.65	2
10	0	43.53	2
10	0	39	2
10	0	47.4	2
10	0	39	1
10	0	37.75	2
10	0	44.06	2
10	0	45.41	1
10	0	38.46	2
10	0	40.11	2
10	0	43.73	2
10	0	45.63	2
10	0	39.43	2
10	0	43.1	2
10	0	35.19	1
10	0	45.63	2
10	0	40	1
10	0	41.53	2
10	0	41.15	2
10	0	42.05	2
10	0	44.8	2
10	0	41.97	2
10	0	46.34	2
10	0	42.03	1

10	0	45.96	1
10	0	52.15	2
10	0	45.22	2
10	0	49.05	2
10	0	44.44	1
10	0	50.17	2
10	0	37.67	2
10	0	42.16	1
10	0	45.66	2
10	0	40.65	2
10	0	53.34	2
10	0	41.92	2
10	0	37.15	2
10	0	47.17	2
10	0	41.93	2
10	0	43.93	2
10	0	45.51	2
10	0	44.78	2
10	0	45.42	2
10	0	48.51	2
10	0	41.71	2
10	0	32.15	2
10	0	41.21	2
10	0	39	2
10	0	41.4	2
10	0	45.79	2
10	0	44	2
10	0	44.97	2
10	0	43.17	2
10	0	41.16	2
10	0	39	2
10	0	32.17	1
10	0	44.8	2
10	0	43	2
10	0	44.18	2
10	0	37	2
10	0	41.71	2
10	0	45.99	2
10	0	42.91	2
10	0	48.52	1
10	0	43.49	2
10	0	41.92	2
10	0	46.17	2
10	0	48.88	2
10	0	48.81	2
10	0	46.5	2
10	0	40.75	1

10	0	43.49	1
10	0	48.55	2
10	0	40.56	2
10	0	41.59	2
10	0	40	1
10	0	40	2
10	0	42.74	2
10	0	41.98	2
10	0	38	2
10	0	39.86	1
10	0	48.1	2
10	0	41.47	1
10	0	47.8	2
10	0	45.77	2
10	0	40.86	1
10	0	41.19	1
10	0	43.61	2
10	0	45.93	2
10	0	46.02	2
10	0	40.9	2
10	0	45.35	1
10	0	43.69	2
10	0	39.54	2
10	0	42.98	2
10	0	38.21	2
10	0	38.65	2
10	0	46.31	2
10	0	41.64	2
10	0	38.64	1
10	0	49.54	2
10	0	38.11	2
10	0	44	2
10	0	49.28	2
10	0	31.1	2
10	0	41.19	2
10	0	42.52	2
10	0	37	1
10	0	42.98	2
10	0	36.29	2
10	0	39	2
10	0	47	1
10	0	43.52	2
10	0	35.19	2
10	0	43.1	2
10	0	37	1
10	0	45.66	1
10	0	48.84	2

10	0	40.68	1
10	0	39	2
10	0	43.2	2
10	0	42.95	2
10	0	40	1
10	0	43.77	2
10	0	41.18	2
10	0	37.08	1
10	0	39.65	2
10	0	43.41	2
10	0	49.53	2
10	0	46.1	2
10	0	34	1
10	0	45.02	2
10	0	39.43	1
10	0	44.95	2
10	0	42.5	2
10	0	51.2	2
10	0	41.92	1
10	0	41.82	2
10	0	39.67	2
10	0	43.29	1
10	0	36	2
10	0	54.51	2
10	0	40	2
10	0	36	2
10	0	43.55	2
10	0	52.46	2
10	0	39	1
10	0	42.58	2
10	0	40.53	1
10	0	34	1
10	0	42.07	1
10	0	38.57	1
10	0	39.57	2
10	0	45.09	2
10	0	32.89	1
10	0	43.8	1
10	0	41.79	2
10	0	42.29	2
10	0	46.71	2
10	0	32.28	2
10	0	46.4	2
10	0	54.33	2
10	0	45.33	2
10	0	38	2
10	0	42.51	2

10	0	38.17	1
10	0	47.42	2
10	0	42.78	2
10	0	44.07	2
10	0	45.78	2
10	0	42.88	1
10	0	44.46	2
10	0	37	1
10	0	37.24	1
10	0	35.17	1
10	0	36.04	1
10	0	43.66	2
10	0	41.62	1
10	0	44	1
10	0	43.64	2
10	0	39.93	1
10	0	40.74	2
10	0	37	2
10	0	42.03	2
10	0	48.59	2
10	0	41.22	1
10	0	42.5	2
10	0	42.1	2
10	0	43.12	1
10	0	42.1	2
10	0	39.18	2
10	0	43.92	1
10	0	42.7	2
10	0	44.01	2
10	0	36	1
10	0	39	2
10	0	39.17	2
10	0	39	2
10	0	41.71	1
10	0	44.67	2
10	0	37	1
10	0	42.98	2
10	0	39.29	2
10	0	40.9	2
10	0	36	2
10	0	41.2	1
10	0	45.31	2
10	0	42.11	2
10	0	43.27	2
10	0	47.97	2
10	0	40.68	2
10	0	39.22	2

10	0	48.22	2
10	0	38.53	1
10	0	29.44	1
10	0	27.17	2
10	0	36.36	2
10	0	42.41	2
10	0	44.61	2
10	0	41.94	2
10	0	37.58	2
10	0	39	2
10	0	42.51	2
10	0	45.09	2
10	0	34	1
10	0	33	1
10	0	43.65	2
10	0	42.12	2
10	0	39	1
10	0	45.95	2
10	0	38	2
10	0	46.74	1
10	0	43.19	1
10	0	41.87	2
10	0	42.66	2
10	0	44.3	2
10	0	41.53	1
10	0	39.6	1
10	0	40	2
10	0	33	1
10	0	35	2
10	0	44.7	1
10	0	44.39	2
10	0	54.95	2
10	0	36	1
10	0	43.11	1
10	0	38.56	2
10	0	37.11	1
10	0	40	2
10	0	46.82	2
10	0	39.36	2
10	0	48.15	2
10	0	35	1
10	0	44.15	1
10	0	35.56	2
10	0	44.13	2
10	0	46.51	2
10	0	29.55	1
10	0	47.69	2

10	0	44.4	2
10	0	44.12	2
10	0	46.62	2
10	0	37.1	2
10	0	38.01	2
10	0	36.53	1
10	0	37.89	2
10	0	46.18	2
10	0	44.45	2
10	0	43.62	2
10	0	45.18	2
10	0	46.73	2
10	0	40	1
10	0	40.96	2
10	0	42.14	2
10	0	39.57	1
10	0	49.08	2
10	0	45.76	2
10	0	37.46	1
10	0	40.64	2
10	0	38.18	1
10	0	54.84	2
10	0	41.4	2
10	0	35.85	1
10	0	41.75	2
10	0	43.5	2
10	0	47.3	2
10	0	37.99	1
10	0	41.08	1
10	0	37	2
10	0	40.92	2
10	0	38.01	1
10	0	47.82	2
10	0	38.86	1
10	0	44.82	2
10	0	44.15	2
10	0	46.97	2
10	0	31.22	2
10	0	36.99	2
10	0	41.34	2
10	0	41.46	2
10	0	36.71	2
10	0	48.54	2
10	0	41.72	2
10	0	42.98	1
10	0	32	1
10	0	39.97	2

10	0	40.53	1
10	0	47.39	1
10	0	43.72	2
10	0	45.66	2
10	0	39.43	1
10	0	41.84	1
10	0	40.67	2
10	0	46	2
10	0	41.03	2
10	0	41.35	1
10	0	41.71	1
10	0	45.92	2
10	0	35.9	2
10	0	38.65	1
10	0	37	1
10	0	37.68	2
10	0	42.81	1
10	0	45.17	2
10	0	35	1
10	0	42.53	2
10	0	51.12	2
10	0	50.47	2
10	0	42.97	2
10	0	33.54	1
10	0	40	2
10	0	42.67	2
10	0	40.61	2
10	0	45.36	1
10	0	38	2
10	0	48.38	2
10	0	38	2
10	0	39	1
10	0	44.98	1
10	0	42.52	2
10	0	44.87	2
10	0	40.79	1
10	0	45.38	1
10	0	37.68	1
10	0	32.68	1
10	0	35	1
10	0	47.63	2
10	0	44.14	2
10	0	52.63	2
10	0	40.18	2
10	0	44.66	2
10	0	36.5	1
10	0	44.67	1

10	0	49.89	2
10	0	37	2
10	0	40.78	1
10	0	41.47	2
10	0	45.06	2
10	0	42.82	2
10	0	35.31	1
10	0	35.78	1
10	0	41.18	2
10	0	37.33	1
10	0	42.32	2
10	0	43.78	2
10	0	40	2
10	0	39.82	2
10	0	38.86	2
10	0	46.67	2
10	0	44.56	2
10	0	39	2
10	0	37.14	1
10	0	41.41	1
10	0	39.4	2
10	0	32.69	1
10	0	40.47	1
10	0	50.03	2
10	0	48.51	2
10	0	47.26	2
10	0	35	1
10	0	39.47	2
10	0	40	1
10	0	45.52	2
10	0	42.9	2
10	0	41.57	2
10	0	37.67	2
10	0	45.88	2
10	0	45.35	1
10	0	45	2
10	0	37.14	1
10	0	43.87	1
10	0	45.43	1
10	0	45.19	2
10	0	39	1
10	0	42.34	1
10	0	39.65	2
10	0	39.94	1
10	0	42.64	2
10	0	43.53	2
10	0	41.79	1

10	0	44.49	2
10	0	39.96	1
10	0	43.45	2
10	0	40.01	1
10	0	40	1
10	0	42.3	2
10	0	45.55	2
10	0	37	1
10	0	50.74	2
10	0	38.58	1
10	0	42.57	2
10	0	31.13	1
10	0	37.06	1
10	0	39.46	2
10	0	45.34	2
10	0	41.36	2
10	0	42.01	2
10	0	51.7	2
10	0	51.1	2
10	0	44.63	2
10	0	44.07	2
10	0	43.21	2
10	0	39.33	2
10	0	39	2
10	0	41.05	1
10	0	52.36	2
10	0	39	1
10	0	45.19	2
10	0	43.9	1
10	0	44.22	2
10	0	36	1
10	0	39.43	2
10	0	46.9	2
10	0	45.59	2
10	0	41.65	2
10	0	46.3	2
10	0	47.39	2
10	0	55.23	2
10	0	36.5	1
10	0	46.95	2
10	0	52.33	2
10	0	43.79	2
10	0	42.67	2
10	0	48.53	2
10	0	38	1
10	0	39.31	2
10	0	40.99	2

10	0	40.51	1
10	0	40.56	1
10	0	46.58	2
10	0	43.59	2
10	0	36.1	1
10	0	42.3	2
10	0	42.03	2
10	0	33.4	1
10	0	42.08	2
10	0	42.12	1
10	0	38.54	2
10	0	47.07	1
10	0	35.99	2
10	0	51.43	2
10	0	44.6	2
10	0	41.51	2
10	0	40.28	2
10	0	50.39	2
10	0	43.79	2
10	0	41.34	1
10	0	42.77	1
10	0	48.22	2
10	0	45.71	2
10	0	34.54	2
10	0	43.41	2
10	0	44.88	2
10	0	44.97	2
10	0	39	2
10	0	37	1
10	0	39.76	2
10	0	39.89	2
10	0	42.55	2
10	0	48.29	1
10	0	35.31	1
10	0	47.72	2
10	0	42.39	2
10	0	37.25	2
10	0	47.34	2
10	0	30.73	1
10	0	36	1
10	0	36.39	2
10	0	33.15	2
10	0	40.1	2
10	0	42.26	2
10	0	39.64	1
10	0	43.64	1
10	0	46.95	2

10	0	46.76	1
10	0	39.19	2
10	0	39	1
10	0	40.26	2
10	0	48.44	2
10	0	34	2
10	0	48.49	2
10	0	43.22	2
10	0	39	2
10	0	37.64	2
10	0	42.7	2
10	0	48.9	1
10	0	37.54	1
10	0	38.28	1
10	0	43.85	2
10	0	46.85	2
10	0	40.9	1
10	0	44.79	2
10	0	36.9	1
10	0	36	2
10	0	46.62	2
10	0	48.16	2
10	0	48.82	2
10	0	37.03	2
10	0	48.42	2
10	0	44.01	2
10	0	35.76	2
10	0	42.46	2
10	0	44.3	2
10	0	46.97	2
10	0	52.58	2
10	0	34.71	2
10	0	39	2
10	0	39.94	1
10	0	45.7	1
10	0	44.93	2
10	0	44.03	2
10	0	40	1
10	0	45.94	2
10	0	37.92	2
10	0	45.28	1
10	0	48.5	2
10	0	47.71	2
10	0	42.7	1
10	0	43.85	1
10	0	41.39	1
10	0	45.12	2

10	0	43.46	2
10	0	41.92	2
10	0	42.5	2
10	0	42.47	1
10	0	46.14	2
10	0	37	2
10	0	43.39	2
10	0	47.39	2
10	0	44.17	1
10	0	47.73	2
10	0	41.16	1
10	0	42.29	2
10	0	37.78	2
10	0	31.93	1
10	0	34	1
10	0	43.81	2
10	0	38.46	1
10	0	40.96	1
10	0	38	2
10	0	46.4	2
10	0	42.99	2
10	0	34.5	1
10	0	49.14	2
10	0	39.75	2
10	0	40.96	1
10	0	42.01	1
10	0	48.76	2
10	0	38	1
10	0	34.22	1
10	0	32	1
10	0	32.65	2
10	0	42.86	2
10	0	45.46	2
10	0	41.19	2
10	0	42.74	2
10	0	39	1
10	0	35.43	1
10	0	38	1
10	0	41.43	2
10	0	47.58	2
10	0	41.36	1
10	0	40	2
10	0	37	2
10	0	49.33	2
10	0	50.14	2
10	0	34.26	1
10	0	44.5	2

10	0	43.94	1
10	0	42.04	1
10	0	34.33	1
10	0	39.25	1
10	0	33.74	1
10	0	39	1
10	0	40	2
10	0	47.42	2
10	0	40.06	2
10	0	31.49	1
10	0	42.07	2
10	0	37.81	1
10	0	49.23	2
10	0	46.05	2
10	0	38.82	1
10	0	39.15	1
10	0	38	1
10	0	49.14	2
10	0	37.51	1
10	0	45.69	1
10	0	39.6	2
10	0	44.18	2
10	0	39.53	2
10	0	41.21	2
10	0	47.82	2
10	0	41.13	1
10	0	44.17	2
10	0	35	2
10	0	43.97	2
10	0	42.08	2
10	0	38.96	2
10	0	36.07	2
10	0	45.25	1
10	0	37.26	2
10	0	40.61	1
10	0	38.92	1
10	0	40.21	2
10	0	43.72	2
10	0	40.9	2
10	0	40	2
10	0	41.05	2
10	0	40.68	2
10	0	33.76	1
10	0	42.46	2
10	0	36	1
10	0	43.74	2
10	0	45.68	2

10	0	40	2
10	0	43.79	2
10	0	43.31	1
10	0	43.45	1
10	0	37.24	2
10	0	31.54	1
10	0	41.99	2
10	0	43.12	1
10	0	36.24	1
10	0	45.79	2
10	0	39	1
10	0	42.87	2
10	0	36	1
10	0	41.01	2
10	0	44.43	2
10	0	39.01	2
10	0	38.71	2
10	0	42.92	1
10	0	41.48	2
10	0	32.81	1
10	0	34.18	2
10	0	31.48	1
10	0	39	2
10	0	40.17	2
10	0	35.11	2
10	0	39	2
10	0	43	2
10	0	38	2
10	0	44.47	2
10	0	44.84	2
10	0	40.43	1
10	0	37.4	1
10	0	41.13	2
10	0	41.39	2
10	0	41.17	2
10	0	41.64	2
10	0	42.68	1
10	0	53.81	2
10	0	43.26	1
10	0	45.02	2
10	0	42.64	2
10	0	51.47	2
10	0	51.22	2
10	0	36.28	1
10	0	39	2
10	0	39.43	2
10	0	47.1	2

10	0	44.29	2
10	0	38.5	2
10	0	46	2
10	0	44.55	2
10	0	39	2
10	0	38.81	1
10	0	36.6	1
10	0	45.23	2
10	0	47.36	2
10	0	36.04	2
10	0	45.84	2
10	0	36	2
10	0	34	1
10	0	36.28	2
10	0	36	2
10	0	37.11	2
10	0	45.16	2
10	0	44.23	2
10	0	38.39	2
10	0	38	2
10	0	37.26	1
10	0	48.49	2
10	0	38.31	2
10	0	39.43	1
10	0	41.66	1
10	0	38.42	2
10	0	47.03	2
10	0	44.57	2
10	0	42.92	2
10	0	39.42	1
10	0	37	1
10	0	41.5	1
10	0	38	2
10	0	41.37	2
10	0	43.37	2
10	0	39	2
10	0	36	1
10	0	37.56	1
10	0	41.31	2
10	0	35	1
10	0	35.06	1
10	0	36.04	1
10	0	41.58	2
10	0	40.32	1
10	0	44.99	2
10	0	39.68	1
10	0	40.04	2

10	0	39	2
10	0	30.51	1
10	0	36.26	2
10	0	42.95	2
10	0	40.65	2
10	0	45.64	2
10	0	34.15	2
10	0	41.44	2
10	0	40.01	1
10	0	42.98	2
10	0	36.32	2
10	0	47.16	2
10	0	40.46	1
10	0	35	2
10	0	31.96	1
10	0	38.53	2
10	0	29.49	1
10	0	42.54	2
10	0	45.98	2
10	0	38.93	1
10	0	41.92	2
10	0	41.73	1
10	0	33.25	1
10	0	40.72	2
10	0	43.16	1
10	0	49.78	2
10	0	41.95	2
10	0	41.16	2
10	0	40	1
10	0	40.69	2
10	0	42.95	1
10	0	40	1
10	0	36.47	1
10	0	43.48	2
10	0	44.99	2
10	0	34.43	1
10	0	49.31	2
10	0	48.09	2
10	0	45.98	2
10	0	43.11	1
10	0	43.9	2
10	0	47.68	2
10	0	36.58	2
10	0	42.18	2
10	0	36.33	2
10	0	35.78	1
10	0	40.78	1

10	0	42.44	2
10	0	42.29	1
10	0	45.31	2
10	0	40.64	1
10	0	47.09	2
10	0	35	1
10	0	44.27	2
10	0	45.09	2
10	0	33	1
10	0	41.87	2
10	0	40	2
10	0	36	1
10	0	37.99	2
10	0	42.99	1
10	0	36.03	2
10	0	46.22	2
10	0	39.49	1
10	0	39.46	2
10	0	38.68	2
10	0	38	1
10	0	33.53	1
10	0	40	1
10	0	41.17	2
10	0	43.54	2
10	0	33.51	1
10	0	34	1
10	0	37.43	2
10	0	43.77	2
10	0	36	2
10	0	41.9	2
10	0	40.24	1
10	0	29.63	1
10	0	41.78	2
10	0	31.11	1
10	0	35.24	1
10	0	42.41	1
10	0	35	2
10	0	34.65	2
10	0	37	1
10	0	37	2
10	0	48.13	2
10	0	41.16	2
10	0	42.1	2
10	0	38.01	1
10	0	44.51	2
10	0	42.71	1
10	0	46.71	2

10	0	35	2
10	0	42.27	2
10	0	38.58	2
10	0	51.06	2
10	0	52.73	2
10	0	43.07	2
10	0	43.48	2
10	0	45.33	2
10	0	37	1
10	0	43.31	2
10	0	39	2
10	0	33.26	2
10	0	31.06	1
10	0	38.58	1
10	0	43.58	2
10	0	34	1
10	0	33.43	2
10	0	43.76	2
10	0	36	2
10	0	37.22	1
10	0	37.1	2
10	0	31.98	1
10	0	34.39	1
10	0	40.31	2
10	0	39.24	1
10	0	39	2
10	0	40.78	2
10	0	43.2	2
10	0	34	1
10	0	40.53	2
10	0	41.71	2
10	0	52.42	2
10	0	37.97	1
10	0	45.26	2
10	0	47.65	2
10	0	44.9	2
10	0	41.37	2
10	0	42.21	2
10	0	41.81	2
10	0	44.95	2
10	0	45.66	2
10	0	36	1
10	0	34.93	2
10	0	34.6	2
10	0	40	2
10	0	47.38	2
10	0	38.86	2

10	0	37.9	1
10	0	37.28	1
10	0	41.71	2
10	0	44.76	2
10	0	42.73	2
10	0	33.96	2
10	0	41.75	2
10	0	40.98	2
10	0	39.72	1
10	0	47.97	2
10	0	40.86	2
10	0	35.72	1
10	0	32	1
10	0	42.82	2
10	0	39.79	2
10	0	38	2
10	0	44.97	2
10	0	48.97	2
10	0	44.46	2
10	0	48.21	2
10	0	40.78	2
10	0	39	2
10	0	43.14	2
10	0	42.77	1
10	0	42.1	2
10	0	38.35	2
10	0	44.54	2
10	0	41.73	1
10	0	37.36	1
10	0	43.91	2
10	0	36.31	2
10	0	47.88	2
10	0	39.89	1
10	0	34.93	2
10	0	41.19	2
10	0	42.41	2
10	0	33.67	1
10	0	39	2
10	0	43.08	2
10	0	34.65	2
10	0	39	2
10	0	43.44	2
10	0	44.63	2
10	0	34.43	2
10	0	39.61	2
10	0	44.58	2
10	0	51.61	2

10	0	34.94	1
11	1	44.91	2
11	1	41.39	1
11	1	37	1
11	1	36.46	1
11	1	38.08	1
11	1	51.37	1
11	1	41.34	2
11	1	36.81	1
11	1	43.42	1
11	1	41.25	2
11	1	50.95	2
11	1	41.08	2
11	1	54.86	1
11	1	47.26	1
11	1	45.01	2
11	1	35.08	1
11	1	51.09	1
11	1	50.17	2
11	1	42.41	1
11	1	32.71	1
11	1	42.99	2
11	1	40.89	1
11	1	44.55	2
11	1	40.86	2
11	1	51.68	1
11	1	44.63	2
11	1	42.73	2
11	1	51.49	2
11	1	45.7	2
11	1	43.44	1
11	1	49.6	1
11	1	57.47	2
11	1	32.17	1
11	1	31.7	1
11	1	42.55	1
11	1	37.22	2
11	1	35.94	1
11	1	37.14	2
11	1	37.74	1
11	1	45.34	1
11	1	36.86	2
11	1	44.99	2
11	1	30.96	2
11	1	47.24	2
11	1	37.31	1
11	1	36.24	1

11	1	37.19	1
11	1	42.44	2
11	1	36	1
11	1	43.92	1
11	1	42.16	2
11	1	40.1	2
11	1	39.93	2
11	1	35.79	1
11	1	34.06	1
11	1	33	1
11	1	54.35	2
11	1	48.3	2
11	1	42	1
11	1	35.33	1
11	1	31.84	1
11	1	29.54	2
11	1	46.56	2
11	1	38.54	2
11	1	40	1
11	1	46.26	2
11	1	47.74	2
11	1	42.39	2
11	1	40.96	1
11	1	48.59	1
11	1	44.95	1
11	1	50.98	1
11	1	30.19	2
11	1	40.51	2
11	1	29.58	2
11	1	39.61	1
11	1	40	1
11	1	44.62	1
11	1	40	1
11	1	34.33	1
11	1	28.97	1
11	1	42.58	1
11	0	45.89	2
11	0	40.25	2
11	0	41.35	2
11	0	42.7	2
11	0	42.49	2
11	0	42.2	2
11	0	35.53	1
11	0	49.85	2
11	0	37.79	1
11	0	43.59	2
11	0	38	1

11	0	47.94	2
11	0	50.42	2
11	0	44.4	2
11	0	38.89	2
11	0	49.59	2
11	0	40.33	2
11	0	37.11	1
11	0	51.1	2
11	0	55.52	2
11	0	35.24	1
11	0	42.74	2
11	0	37.26	1
11	0	42.27	1
11	0	40	2
11	0	44.97	2
11	0	46.8	1
11	0	48.12	2
11	0	39.67	2
11	0	43.94	2
11	0	47.93	2
11	0	46.66	2
11	0	41.52	2
11	0	34.99	2
11	0	38	2
11	0	41.22	2
11	0	53.04	2
11	0	50.11	2
11	0	37.97	2
11	0	48.44	2
11	0	43.01	2
11	0	41.18	1
11	0	54.05	2
11	0	43.02	2
11	0	44.75	2
11	0	34.74	2
11	0	41.28	2
11	0	33	2
11	0	48.68	2
11	0	47.65	2
11	0	37	2
11	0	55.33	2
11	0	40.68	2
11	0	49.15	2
11	0	40.89	2
11	0	49.73	2
11	0	45.41	2
11	0	39.85	2

11	0	55.16	2
11	0	36	2
11	0	44.56	2
11	0	42.04	2
11	0	41.91	2
11	0	39.86	2
11	0	46.38	2
11	0	41.13	2
11	0	33	1
11	0	37	2
11	0	49.94	2
11	0	35.54	2
11	0	51.73	2
11	0	39.57	1
11	0	29.54	2
11	0	36	2
11	0	39.33	2
11	0	46.2	2
11	0	46.43	2
11	0	46.95	2
11	0	60.13	2
11	0	45.04	2
11	0	39	2
11	0	50.81	2
11	0	41.45	2
11	0	48.01	2
11	0	48.56	2
11	0	41.65	2
11	0	51.18	1
11	0	49.96	2
11	0	44.95	2
11	0	35.86	2
11	0	41.01	2
11	0	45.61	2
11	0	37.14	2
11	0	45.86	2
11	0	47.83	2
11	0	48.2	2
11	0	45.35	2
11	0	54.97	2
11	0	48.88	2
11	0	36.65	2
11	0	39.39	2
11	0	48.38	2
11	0	52.1	1
11	0	61.17	2
11	0	56.41	2

11	0	43.46	2
11	0	44.29	2
11	0	49	2
11	0	54.42	2
11	0	42.66	2
11	0	47	2
11	0	51.46	2
11	0	42	2
11	0	39	2
11	0	45.61	2
11	0	42.14	2
11	0	40	2
11	0	52.45	2
11	0	49.91	2
11	0	37.28	2
11	0	46.06	2
11	0	41.85	2
11	0	43.19	2
11	0	46.28	2
11	0	45.46	2
11	0	42.15	2
11	0	45.44	2
11	0	44.46	2
11	0	47.99	2
11	0	38.65	2
11	0	44.7	2
11	0	45.27	2
11	0	42.98	2
11	0	32.47	2
11	0	42.87	2
11	0	54.72	2
11	0	35.65	2
11	0	39	1
11	0	43.02	1
11	0	45.41	2
11	0	33.68	2
11	0	41.34	2
11	0	37	2
11	0	36.76	2
11	0	39.44	2
11	0	34.97	1
11	0	45.9	2
12	1	45.21	1
12	1	45.4	1
12	1	39	1
12	1	58.23	1
12	1	45.24	1

12	1	37.26	1
12	1	37.64	1
12	1	34	1
12	1	37.47	1
12	1	37.72	1
12	1	31.9	1
12	1	33.9	2
12	1	35.51	1
12	1	31.69	1
12	1	27.66	1
12	1	38.24	1
12	1	35.01	1
12	1	35.15	1
12	1	32.42	1
12	1	28.98	1
12	1	37.4	1
12	1	42.65	1
12	1	36	1
12	1	39.64	1
12	1	41.33	1
12	1	38.9	1
12	1	41.14	1
12	1	37	1
12	1	41.29	1
12	1	26.03	1
12	1	32	1
12	1	32	1
12	1	32	1
12	1	35	1
12	1	37.33	1
12	1	44.33	2
12	1	36.4	1
12	1	42.4	1
12	1	41.68	2
12	1	31.5	1
12	1	36	1
12	1	31.76	1
12	1	35	1
12	1	43.9	2
12	1	44.05	2
12	1	28.06	1
12	1	36.11	1
12	0	48.16	1
12	0	51.7	2
12	0	40.57	2
12	0	45.76	2
12	0	47.77	2

12	0	43.91	2
12	0	38.72	1
12	0	51.31	2
12	0	44.17	2
12	0	54.83	2
12	0	52.07	1
12	0	48.66	1
12	0	46.89	2
12	0	40.29	1
12	0	39.54	1
12	0	46.18	2
12	0	52.4	2
12	0	40.06	1
12	0	50.67	2
12	0	37	1
12	0	40.58	1
12	0	42.76	2
12	0	48.7	2
12	0	41.07	1
12	0	42.81	1
12	0	35.43	1
12	0	39.53	1
12	0	40.22	2
12	0	45.48	2
12	0	42.21	1
12	0	38	1
12	0	44.33	2
12	0	45.7	1
12	0	46.81	2
12	0	41.76	2
12	0	39.5	2
12	0	47.76	2
12	0	45.91	1
12	0	47.66	2
12	0	47.97	2
12	0	39	2
12	0	43.66	2
12	0	43.78	2
12	0	43.04	1
12	0	47.2	1
12	0	44.14	2
12	0	48.54	2
12	0	41.95	1
12	0	49.01	2
12	0	39.96	1
12	0	46.93	2
12	0	38.54	2

12	0	47.24	2
12	0	42.32	2
12	0	39.53	1
12	0	38.5	2
12	0	33	1
12	0	43.41	2
12	0	51.58	2
12	0	36.54	1
12	0	46.28	2
12	0	44.3	1
12	0	43.8	1
12	0	39.68	2
12	0	38.35	2
12	0	56.34	2
12	0	44.58	1
12	0	48.52	2
12	0	44.16	1
12	0	28.53	1
12	0	44.25	2
12	0	41.79	2
12	0	45.53	2
12	0	41.74	2
12	0	49.18	2
12	0	42.58	2
12	0	44.84	2
12	0	38.39	1
12	0	43.04	1
12	0	43.73	1
12	0	40.1	1
12	0	45.05	1
12	0	45.39	2
12	0	42.71	1
12	0	41.03	1
12	0	39.4	2
12	0	44.58	2
12	0	43.67	2
12	0	44.28	2
12	0	47.54	2
12	0	41.9	1
12	0	42.56	2
12	0	34.15	2
12	0	40.9	2
12	0	34.82	2
12	0	40.24	2
12	0	54.35	2
12	0	49.43	2
12	0	41.21	1

12	0	42.44	2
12	0	43.23	2
12	0	40.29	2
12	0	40	2
12	0	44.74	1
12	0	42.96	2
12	0	41.64	2
12	0	41.23	2
12	0	37	2
12	0	45.96	2
12	0	45.54	2
12	0	52.46	2
12	0	37.28	1
12	0	39	2
12	0	53.05	2
12	0	41.25	2
12	0	37.97	1
12	0	41.42	2
12	0	40.46	2
12	0	44.17	2
12	0	34.72	1
12	0	46.04	2
12	0	50.33	2
12	0	39.93	2
12	0	40	2
12	0	37.39	1
12	0	38.53	2
12	0	46.61	2
12	0	46.23	2
12	0	36.26	1
12	0	40.1	1
12	0	50.31	2
12	0	48.01	2
12	0	36.94	2
12	0	36.35	2
12	0	50.11	2
12	0	46.57	2
12	0	40.9	2
12	0	46.5	2
12	0	42.54	2
12	0	40.26	1
12	0	47.38	2
12	0	43.54	2
12	0	37	2
12	0	50.22	2
12	0	44.88	2
12	0	42.52	2

12	0	42.83	2
12	0	49.57	2
12	0	41.57	2
12	0	48.15	2
12	0	44	2
12	0	43.95	2
12	0	37.58	1
12	0	41.98	1
12	0	45.96	2
12	0	40	1
12	0	39	1
12	0	43.92	2
12	0	50.62	2
12	0	48.12	2
12	0	46.24	2
12	0	42.92	1
12	0	38.58	2
12	0	39.1	2
12	0	48.55	2
12	0	47.16	2
12	0	53.93	2
12	0	41.19	2
12	0	42.58	2
12	0	38	2
12	0	55.07	2
12	0	45.4	1
12	0	38.4	2
12	0	41.82	2
12	0	46.12	2
12	0	45.7	2
12	0	47.3	2
12	0	45.27	2
12	0	42.82	2
12	0	49.46	2
12	0	40.18	1
12	0	41.7	1
12	0	36.97	1
12	0	43.33	2
12	0	38.47	2
12	0	40.24	2
12	0	39.54	2
12	0	35	1
12	0	50.78	2
12	0	45.42	2
12	0	44.32	2
12	0	44.91	2
12	0	42.77	2

12	0	41.4	2
12	0	38.33	1
12	0	45.58	2
12	0	50.45	2
12	0	43.99	2
12	0	41.06	2
12	0	43.33	2
12	0	46.56	2
12	0	41.08	1
12	0	38.35	2
12	0	52.39	2
12	0	49.03	2
12	0	37.72	2
12	0	45.61	2
12	0	41.17	2
12	0	46.62	1
12	0	38.72	2
12	0	37.1	1
12	0	42.16	2
12	0	54.6	2
12	0	47.61	2
12	0	44.19	2
12	0	40	2
12	0	32.15	2
12	0	38.94	2
12	0	54.72	2
12	0	34	1
12	0	40.19	1
12	0	48.75	2
12	0	40.47	2
12	0	37.74	2
12	0	39.08	1
12	0	42.57	1
12	0	43.02	1
12	0	47.4	2
12	0	38	2
12	0	49.74	2
12	0	44.53	2
12	0	51.61	2
12	0	51	2
12	0	48.61	2
12	0	46.6	2
12	0	34.93	2
12	0	40.86	2
12	0	34.1	2
12	0	47.26	2
12	0	46.36	2

12	0	43.79	2
12	0	41.39	1
12	0	40.89	2
12	0	48.32	2
12	0	48.29	2
12	0	43.4	2
12	0	46.55	1
12	0	39.72	1
12	0	47.36	2
12	0	45.92	1
12	0	50.16	2
12	0	48.59	2
12	0	41.56	2
12	0	42.28	2
12	0	41.64	2
12	0	37.29	2
12	0	43.61	2
12	0	44.99	2
12	0	40.19	2
12	0	45.33	2
12	0	42.54	2
12	0	39.6	2
12	0	41.8	2
12	0	41.96	2
12	0	47.35	2
12	0	43.22	2
12	0	34	1
12	0	36	2
12	0	37.65	2
12	0	39	2
12	0	44.55	2
12	0	43.97	2
12	0	36.6	1
12	0	34.97	1
12	0	39	2
12	0	38.4	2
12	0	40	2
12	0	40.54	2
12	0	38.68	1
12	0	43.78	2
12	0	49.15	2
12	0	48.49	2
12	0	39.04	2
12	0	40	2
12	0	47.27	2
12	0	50.89	2
12	0	39	2

12	0	39.5	2
12	0	41.66	2
12	0	39.11	2
12	0	37.93	2
12	0	51.07	2
12	0	43.74	2
12	0	43.41	2
12	0	38.54	2
12	0	40	2
12	0	34.81	1
12	0	40.44	1
12	0	38.31	2
12	0	42.45	2
14	1	55.1	2
14	1	52.55	2
14	1	46.43	2
14	1	43.57	1
14	1	44.34	2
14	1	47.79	2
14	1	40.22	1
14	1	45.64	1
14	1	40.74	1
14	1	44.12	2
14	1	46.25	2
14	1	33	1
14	1	44.83	2
14	1	34	2
14	1	43.41	1
14	1	44.39	1
14	1	47.22	2
14	1	40.35	2
14	1	39.32	1
14	1	32.74	1
14	1	42.52	2
14	1	38.65	2
14	1	42.6	2
14	1	40	2
14	1	51.16	2
14	1	28.77	1
14	0	51.42	2
14	0	44.33	2
14	0	50.37	2
14	0	49.03	2
14	0	44.13	2
14	0	45.29	2
14	0	45.25	1
14	0	46.41	2

14	0	40.78	2
14	0	53.54	2
14	0	42.11	2
14	0	50.19	2
14	0	50.79	2
14	0	38.9	1
14	0	52.14	2
14	0	48.54	2
14	0	49.22	1
14	0	48.97	2
14	0	43.27	2
14	0	50.46	1
14	0	44.55	2
14	0	45.69	2
14	0	49.7	2
14	0	43.51	2
14	0	47.88	2
14	0	45.4	1
14	0	55.21	2
14	0	53.96	2
14	0	56.37	2
14	0	44.14	2
14	0	52	2
14	0	41.78	2
14	0	35	1
14	0	48.44	2
14	0	41.49	2
14	0	48.28	2
14	0	51.96	2
14	0	50.68	2
14	0	45.63	2
14	0	42.95	2
14	0	55.12	2
14	0	50.07	2
14	0	48.75	1
14	0	55.71	2
14	0	43.85	1
14	0	51.68	1
14	0	48.43	2
14	0	44.11	2
14	0	43.8	2
14	0	51	2
14	0	50.69	2
14	0	49.16	2
14	0	55.03	2
14	0	38.51	1
14	0	45.9	2

14	0	49.45	2
14	0	51.54	2
14	0	46.68	2
14	0	47.6	2
14	0	47.14	2
14	0	35	2
14	0	52.78	2
14	0	45.3	1
14	0	46.34	2
14	0	44.1	2
14	0	48.14	2
14	0	43.4	2
14	0	34.44	2
14	0	48.21	1
14	0	47.02	2
14	0	50.72	2
14	0	54.72	2
14	0	50.48	2
14	0	38.81	2
14	0	54.94	2
14	0	42.74	2
14	0	48.45	2
14	0	46.68	2
14	0	41.72	2
14	0	48.98	2
14	0	48.81	2
14	0	40.17	1
14	0	51	2
14	0	42.87	2
14	0	39.64	2
14	0	50.46	2
14	0	38.51	2
14	0	43.9	1
14	0	45.8	2
14	0	38	2
14	0	47.35	2
14	0	53.6	2
14	0	45.27	2
14	0	47.28	2
14	0	50.31	2
14	0	46.85	2
14	0	47.09	1
14	0	54.44	2
14	0	42.74	2
14	0	42.03	2
14	0	47.93	1
14	0	38	2

14	0	53.01	2
14	0	39.78	2
14	0	35	2
14	0	52.15	2
14	0	40.07	2
14	0	52.62	2
14	0	39.67	1
14	0	37	2
14	0	49.39	2
14	0	47.7	2
14	0	49.96	2
14	0	46.97	2
14	0	46.97	2
14	0	44.31	1
14	0	56.91	2
14	0	44.55	2
14	0	54.82	2
14	0	47.49	1
14	0	46.58	2
14	0	48.07	2
14	0	47.93	2
14	0	38	1
14	0	47.44	2
14	0	36.89	2
14	0	39.82	2
14	0	50.06	2
14	0	43.11	2
14	0	49.05	2
14	0	40	2
14	0	42.93	2
14	0	52.71	2
14	0	45.98	2
14	0	47.42	2
14	0	43.83	1
14	0	44.78	2
14	0	49.88	2
14	0	43.01	2
14	0	37	1
14	0	50.1	2
14	0	49.52	2
14	0	51.2	2
14	0	42.94	2
14	0	40	2
14	0	48.18	2
14	0	51.55	2
14	0	42.08	1
14	0	41.48	2

14	0	35.93	2
14	0	48.96	2
14	0	50.76	2
14	0	45.41	2
14	0	38.15	1
14	0	49.78	2
14	0	44.3	2
14	0	46.94	2
14	0	37	2
14	0	45.9	2
14	0	44.29	2
14	0	54.41	2
14	0	45.08	2
14	0	52.09	2
14	0	45.66	2
14	0	55.2	2
14	0	54.9	2
14	0	40	2
14	0	39.01	2
14	0	49.91	2
14	0	44.34	2
14	0	52.61	2
14	0	51.31	2
14	0	43.8	2
14	0	46.6	2
14	0	43.33	2
14	0	39	2
14	0	41.25	2
14	0	50.8	2
14	0	32	2
14	0	43.97	2
14	0	52.95	2
14	0	50.58	2
14	0	41.95	2
14	0	39	2
14	0	45.21	2
14	0	50.9	2
14	0	45.27	2
14	0	45.49	2
14	0	47.47	2
14	0	44.12	2
14	0	45.34	2
14	0	40.47	2
14	0	42.35	2
14	0	48.9	2
14	0	37.86	2
14	0	47.11	2

14	0	47.49	2
14	0	48.9	2
14	0	41.47	2
14	0	34.36	2
14	0	35	2
14	0	44.1	2
14	0	35	2
17	1	49.8	2
17	1	41.8	1
17	1	42.45	1
17	1	42.04	1
17	1	49.98	2
17	1	39.93	1
17	1	44.34	2
17	1	42.09	2
17	1	44.8	2
17	1	59.55	2
17	1	50.13	2
17	1	37.74	1
17	1	48.26	2
17	1	41.19	1
17	1	42.44	2
17	1	45.98	1
17	1	41.47	2
17	1	43.56	2
17	1	35	2
17	1	43.2	2
17	1	44.35	1
17	1	47.16	1
17	1	47.11	2
17	1	43.75	2
17	1	40.57	2
17	1	54.16	2
17	1	47.6	2
17	1	40.82	2
17	1	40.28	1
17	1	47	1
17	1	55.4	2
17	1	44.81	2
17	1	47.78	2
17	1	50.32	2
17	1	42.6	1
17	1	50.02	2
17	1	39.93	2
17	1	50.62	1
17	1	36	1
17	1	44.84	2

17	1	37.54	2
17	1	43.6	2
17	1	49.26	1
17	1	44.24	2
17	1	46.23	1
17	1	32	1
17	1	34.35	1
17	1	40.21	2
17	1	42.58	2
17	1	43.17	1
17	1	48.04	2
17	1	48.84	2
17	1	48.64	1
17	1	36	2
17	1	42.87	2
17	1	37.25	2
17	1	47.28	2
17	1	43.78	2
17	1	47.45	2
17	1	46.39	2
17	1	40.67	1
17	1	38	1
17	1	50.2	2
17	1	43.1	2
17	1	37.76	2
17	1	37.6	2
17	1	34.56	2
17	1	49.04	2
17	1	40.71	1
17	1	49.95	2
17	1	51.71	2
17	1	47.52	2
17	1	49.52	2
17	1	37	2
17	1	41.63	2
17	1	46.41	2
17	1	51.52	1
17	1	52.85	2
17	1	39.04	2
17	1	38	1
17	1	37.24	1
17	1	49.29	1
17	1	37.25	1
17	1	48.64	2
17	1	46.92	1
17	1	43.93	1
17	1	41.24	2

17	1	47.39	1
17	1	53.64	1
17	1	53.73	2
17	1	38.9	2
17	1	46.28	1
17	1	40.5	1
17	1	45.77	2
17	1	43.55	2
17	1	61.45	2
17	1	43.65	2
17	1	43.98	1
17	1	37	1
17	1	42.62	1
17	1	46.51	2
17	1	46.51	2
17	1	35.72	1
17	1	53.2	2
17	1	48.33	2
17	1	44.24	2
17	1	42	2
17	1	38	2
17	1	43.55	2
17	1	41.65	1
17	0	54.53	2
17	0	47.39	1
17	0	44.92	2
17	0	48.47	2
17	0	44.01	2
17	0	53.85	2
17	0	47.11	1
17	0	42.47	2
17	0	41.31	2
17	0	40.32	1
17	0	47.48	2
17	0	46.24	2
17	0	43.32	2
17	0	48.68	2
17	0	41.67	2
17	0	49.03	1
17	0	43.61	1
17	0	43.9	1
17	0	46.6	2
17	0	48.3	2
17	0	42.6	2
17	0	41.81	1
17	0	46.86	1
17	0	47.2	2

17	0	45.53	1
17	0	42.28	2
17	0	44.06	1
17	0	49.08	2
17	0	48.93	1
17	0	45.36	2
17	0	47.67	2
17	0	46.79	2
17	0	45.33	2
17	0	46.45	2
17	0	46.37	2
17	0	43.97	2
17	0	41.18	2
17	0	43.68	2
17	0	39.75	2
17	0	39	1
17	0	40.43	2
17	0	45.04	2
17	0	51.12	2
17	0	43.12	2
17	0	46.21	2
17	0	47.84	2
17	0	53.16	2
17	0	45.81	1
17	0	42.9	1
17	0	41.48	2
17	0	50.81	2
17	0	48.83	2
17	0	39	2
17	0	45.34	2
17	0	45.44	2
17	0	40.39	1
17	0	42.08	1
17	0	39.24	2
17	0	44.64	2
17	0	41.13	2
17	0	36.47	2
17	0	40.98	2
17	0	44.15	2
17	0	42.48	2
17	0	42.06	2
17	0	42.51	2
17	0	46.4	2
17	0	48.89	2
17	0	48.01	2
17	0	45.16	2
17	0	41.91	2

17	0	52.12	2
17	0	46.15	2
17	0	42.81	1
17	0	43.18	2
17	0	45.03	2
17	0	33.85	2
17	0	41.67	2
17	0	40	2
17	0	43.03	2
17	0	45.55	2
17	0	49.84	1
17	0	40.54	2
17	0	46.17	2
17	0	46.48	2
17	0	50.75	2
17	0	50.38	2
17	0	52.4	2
17	0	48.16	2
17	0	42.99	1
17	0	42.51	2
17	0	46.7	1
17	0	44.45	2
17	0	44	2
17	0	46.95	1
17	0	36.97	2
17	0	39.76	2
17	0	43.35	2
17	0	52.13	2
17	0	42.49	2
17	0	49.61	2
17	0	47.65	2
17	0	42.17	1
17	0	41.86	1
17	0	50.29	2
17	0	51	2
17	0	45.74	2
17	0	45.41	2
17	0	37.26	1
17	0	37.03	1
17	0	43.96	2
17	0	40.33	2
17	0	37.96	1
17	0	46.26	2
17	0	49.51	2
17	0	49.82	2
17	0	40.54	1
17	0	48.56	2

17	0	44.96	2
17	0	47.09	2
17	0	49.64	2
17	0	50	2
17	0	50.42	2
17	0	49.16	2
17	0	50.55	2
17	0	42.67	2
17	0	46.15	2
17	0	45.35	2
17	0	45.13	2
17	0	38.11	1
17	0	41.5	2
17	0	44.21	2
17	0	44	1
17	0	38.96	1
17	0	46.33	2
17	0	51.1	2
17	0	44.34	1
17	0	46.76	2
17	0	47.96	1
17	0	48.67	1
17	0	49.3	2
17	0	51.14	1
17	0	47.91	2
17	0	45.15	1
17	0	39.78	2
17	0	41.26	1
17	0	41.15	2
17	0	47.48	2
17	0	45.47	2
17	0	40.26	2
17	0	53.56	2
17	0	43.46	2
17	0	49.38	1
17	0	47.14	1
17	0	47.13	2
17	0	52.87	2
17	0	47.86	2
17	0	44.1	1
17	0	46.53	2
17	0	47.56	2
17	0	36.35	1
17	0	45.45	2
17	0	44.88	2
17	0	42.73	2
17	0	42.45	1

17	0	46.72	2
17	0	44.17	2
17	0	42.72	1
17	0	49.61	2
17	0	46.39	2
17	0	51.71	2
17	0	49.56	2
17	0	43.49	2
17	0	50.28	2
17	0	50.1	2
17	0	47.53	2
17	0	38.14	1
17	0	49.3	2
17	0	52.46	2
17	0	40.6	1
17	0	41.12	2
17	0	41.77	1
17	0	44.28	1
17	0	43.56	2
17	0	44.51	2
17	0	36.69	2
17	0	50.72	2
17	0	40	1
17	0	39.65	1
17	0	38	1
17	0	50	2
17	0	38.71	2
17	0	45.27	2
17	0	41.82	2
17	0	46.22	1
17	0	46.1	2
17	0	51.56	2
17	0	41.56	1
17	0	51.36	2
17	0	45.76	2
17	0	49.66	2
17	0	41.79	1
17	0	41.14	2
17	0	43.67	2
17	0	37	1
17	0	43.7	2
17	0	45.13	2
17	0	49.03	2
17	0	37.42	1
17	0	38	1
17	0	44.52	2
17	0	52.5	2

17	0	41.7	2
17	0	46.48	1
17	0	45.21	2
17	0	33.92	1
17	0	49.98	2
17	0	55.2	2
17	0	34.24	1
17	0	51.61	2
17	0	40.06	2
17	0	43.52	2
17	0	41.89	2
17	0	46.84	2
17	0	44.8	2
17	0	47.83	2
17	0	43.64	2
17	0	48.97	2
17	0	42.47	2
17	0	42.61	2
17	0	45.45	2
17	0	47.41	2
17	0	46.82	2
17	0	38.26	1
17	0	49	1
17	0	48.69	2
17	0	44.89	1
17	0	47.87	2
17	0	49.36	2
17	0	47.18	2
17	0	43.91	2
17	0	51.8	2
17	0	42.52	2
17	0	49.22	2
17	0	48.4	1
17	0	40.82	1
17	0	45.32	1
17	0	35	1
17	0	47	2
17	0	39.29	2
17	0	44.17	2
17	0	42	1
17	0	48.44	2
17	0	41.39	2
17	0	44.34	2
17	0	43.05	2
17	0	36	2
17	0	41.1	2
17	0	41.47	2

17	0	41.83	2
17	0	40.01	1
17	0	50.57	2
17	0	45.63	2
17	0	41.72	2
17	0	42.29	2
17	0	39.65	1
17	0	48.63	1
17	0	37.4	2
17	0	40	2
17	0	44.5	1
17	0	52.17	2
17	0	45.9	2
17	0	37	1
17	0	45.94	1
17	0	37.54	1
17	0	49.25	2
17	0	44.77	1
17	0	48.58	2
17	0	42.57	1
17	0	41.47	2
17	0	44.42	2
17	0	41.6	2
17	0	44.7	2
17	0	48.45	2
17	0	38	2
17	0	43.62	2
17	0	39.4	2
17	0	41.4	1
17	0	47.18	2
17	0	42.27	2
17	0	49.06	2
17	0	42.75	2
17	0	38	2
17	0	42.64	2
17	0	41.15	2
17	0	43.16	2
17	0	47.24	2
17	0	44.2	1
17	0	44.68	2
17	0	48.62	2
18	1	32	2
18	1	56.93	2
18	1	40.11	1
18	1	42.39	2
18	1	49.26	2
18	1	49.88	1

18	1	45.25	2
18	1	38.57	2
18	1	50.51	1
18	1	43.01	2
18	1	37.94	1
18	1	43.02	2
18	1	45.86	2
18	1	40	1
18	1	47.1	1
18	1	37.36	2
18	1	43.62	1
18	1	48.95	2
18	1	37.69	2
18	1	40.86	2
18	1	42.36	2
18	1	44.01	1
18	1	42.01	2
18	1	40.65	1
18	1	43.94	2
18	1	36.1	1
18	1	39	1
18	1	37.76	2
18	1	47.91	1
18	1	35.93	2
18	1	52.23	2
18	1	43.3	2
18	1	35	1
18	1	38	2
18	1	46.41	2
18	1	39.22	2
18	1	45.24	2
18	1	37.64	1
18	1	41.8	1
18	1	46.62	2
18	1	47.3	2
18	1	32.82	1
18	1	43.2	1
18	1	33.79	2
18	1	41.85	2
18	1	45.94	2
18	1	47.78	1
18	1	38.36	2
18	1	40	1
18	1	37.85	1
18	1	47.62	1
18	1	43.77	1
18	1	36.9	2

18	1	40.29	2
18	1	38	1
18	1	33.82	1
18	1	37	1
18	1	39.04	1
18	1	38.61	2
18	1	52.43	2
18	1	38	1
18	1	39.54	2
18	1	40.82	2
18	1	48	1
18	1	44.18	2
18	1	37.83	1
18	1	45.49	2
18	1	46.84	1
18	1	43.32	2
18	1	36.26	1
18	1	37.51	2
18	1	38.65	2
18	1	43.67	1
18	1	34	2
18	1	40.61	1
18	1	35.42	2
18	1	39.74	2
18	1	44.2	2
18	1	39.83	1
18	1	36.54	1
18	1	38.11	2
18	1	40	2
18	1	39.94	2
18	1	32	1
18	1	49.74	2
18	1	49.36	1
18	1	32.14	1
18	1	32.64	2
18	1	38.71	1
18	1	35.31	1
18	1	37	1
18	1	31.75	1
18	1	49.49	1
18	1	46.12	1
18	1	31.11	2
18	1	33	2
18	1	44.72	1
18	1	42.06	2
18	1	48.39	2
18	1	35.4	1

18	1	43.19	2
18	1	42.98	2
18	1	37.97	2
18	1	32.92	2
18	1	37.36	2
18	1	38	2
18	1	49.05	2
18	1	42.06	2
18	1	41.07	1
18	1	35.07	2
18	1	41.69	2
18	1	36.61	2
18	1	37.79	1
18	1	34.04	1
18	1	35	2
18	1	35.6	2
18	1	40	2
18	1	46.81	1
18	1	32.86	1
18	1	42.17	2
18	1	42.2	2
18	1	36.9	1
18	1	36.51	1
18	1	39.61	1
18	1	37.72	2
18	1	30.74	1
18	1	45.89	2
18	1	33.92	2
18	1	38.33	2
18	1	34.51	1
18	1	35.01	2
18	1	44.55	2
18	1	42.39	1
18	1	35.75	1
18	1	46.89	1
18	1	49.44	1
18	1	43.63	2
18	1	35	1
18	1	40.89	1
18	1	44	1
18	1	40.65	1
18	1	50.12	2
18	1	43.68	2
18	1	37.22	1
18	1	35.75	1
18	1	40	2
18	1	39.5	1

18	1	31.45	1
18	1	38	1
18	1	40	1
18	1	39	1
18	1	37.89	1
18	1	31.95	2
18	1	42.63	1
18	1	30.66	2
18	1	35.33	1
18	1	40.86	1
18	1	42.32	1
18	1	32.24	2
18	1	41.64	2
18	1	35.39	2
18	1	35.97	2
18	1	36.54	2
18	1	35.58	2
18	1	35	1
18	1	34.76	1
18	1	40	1
18	1	37	2
18	1	37	2
18	1	44.81	2
18	1	35.86	2
18	1	40.83	2
18	1	40	2
18	1	39.75	2
18	1	36.46	1
18	1	42.77	2
18	1	42.32	2
18	1	44.8	2
18	1	41.17	2
18	1	37.07	1
18	1	37.29	2
18	1	39	1
18	1	35.46	1
18	1	35.85	2
18	1	38.99	2
18	1	38.29	2
18	1	39.71	2
18	1	40.93	2
18	1	32.07	1
18	1	38	1
18	1	43.72	2
18	1	33.43	1
18	1	31.32	2
18	1	36	2

18	1	31.75	1
18	1	36.33	1
18	1	37	2
18	1	43.04	2
18	1	40.17	1
18	1	46.44	2
18	1	38.06	2
18	1	33.25	1
18	1	46.43	2
18	1	33.82	2
18	1	34	2
18	1	44.61	2
18	1	37.22	1
18	1	49.51	1
18	1	46.57	2
18	1	41.68	2
18	1	34	2
18	1	35.75	2
18	1	35	1
18	1	40.44	2
18	1	40	2
18	1	35.85	2
18	1	35.9	2
18	1	29.13	1
18	1	39.11	2
18	1	35.74	2
18	1	38	2
18	1	32	1
18	1	41.35	2
18	1	44.36	2
18	1	33	2
18	1	44.2	2
18	1	40	2
18	1	35.64	2
18	1	46	2
18	1	40.33	1
18	1	37.29	2
18	1	38.18	2
18	1	40.61	1
18	1	36	2
18	1	35.28	1
18	1	46.21	2
18	1	31.94	1
18	1	33	2
18	1	31.9	2
18	1	32.47	1
18	1	37	1

18	1	45.84	2
18	1	36	1
18	1	38.43	1
18	1	43.1	2
18	1	35	2
18	1	37.82	1
18	1	45.33	2
18	1	34.51	2
18	1	45.1	1
18	1	35	2
18	1	47.98	2
18	1	34.89	2
18	1	41.69	1
18	1	34.82	2
18	1	32.26	1
18	1	40.29	2
18	1	39.22	1
18	1	34.97	1
18	1	36	2
18	1	33.25	1
18	1	40.71	1
18	1	42.3	2
18	1	31.93	2
18	1	36.25	1
18	1	30.68	1
18	1	34.76	2
18	1	42.75	2
18	1	33.32	1
18	1	39.33	1
18	1	33	2
18	1	36	2
18	1	34.61	1
18	1	33.32	2
18	1	31.39	2
18	1	38	2
18	1	31	1
18	1	35.64	2
18	1	37	2
18	1	32.68	1
18	1	32	1
18	1	37.4	2
18	1	30.24	2
18	1	32	1
18	1	34.47	1
18	1	42.35	2
18	1	38.07	2
18	1	32	1

18	1	30.59	1
18	1	29.22	1
18	1	34.4	1
18	1	37	2
18	1	33.35	2
18	1	39	2
18	1	37.32	1
18	1	33.22	2
18	1	35.43	2
18	1	41.25	2
18	1	37.14	2
18	1	31.25	1
18	0	36.57	2
18	0	34	2
18	0	35.53	2
18	0	48.13	2
18	0	44.48	2
18	0	36	2
18	0	34.57	2
18	0	43.12	2
18	0	45.66	2
18	0	36.31	2
18	0	38.24	2
18	0	38	2
18	0	42.05	2
18	0	36.61	2
18	0	37.51	2
18	0	39	2
18	0	40.96	1
18	0	33.61	2
18	0	43.12	2
18	0	34.46	2
18	0	35.6	2
18	0	38.08	2
18	0	34.68	2
18	0	42.92	2
18	0	32.74	2
18	0	41.47	2
18	0	35.85	2
18	0	33	2
18	0	50.2	2
18	0	47.46	1
18	0	36.92	1
18	0	33.33	2
18	0	36	2
18	0	39.08	1
18	0	35.15	2

18	0	35	2
18	0	35	1
18	0	31.68	2
18	0	39.21	1
18	0	30.61	2
18	0	34.64	2
18	0	56.56	2
18	0	39.9	2
18	0	41.42	2
18	0	54.66	2
18	0	36.4	2
18	0	33.53	2
18	0	45.09	1
18	0	46.63	2
18	0	41.45	2
18	0	41.2	2
18	0	38	2
18	0	50.03	1
18	0	33.07	2
18	0	41.17	2
18	0	43.43	2
18	0	44.8	1
18	0	37.28	1
18	0	34	2
18	0	34.74	2
18	0	33.08	2
18	0	43.44	2
18	0	35.18	2
18	0	36.07	2
18	0	38.79	1
18	0	36.06	2
18	0	42.63	2
18	0	47.95	2
18	0	36.42	2
18	0	30.42	2
18	0	33.96	2
18	0	53.37	2
18	0	39	2
18	0	33.61	2
18	0	36	1
18	0	33.47	2
18	0	38.82	2
18	0	31.3	2
18	0	35	2
18	0	34.06	1
18	0	39.72	2
18	0	43.4	2

18	0	35.65	2
18	0	32.36	2
18	0	42.58	2
18	0	41.85	2
18	0	46	2
18	0	53.76	2
18	0	36.82	2
18	0	33.9	2
18	0	36	2
18	0	32	2
18	0	36	2
18	0	38	2
18	0	31.11	2
18	0	36	2
18	0	33.93	2
18	0	34	2
18	0	39.9	2
18	0	35	1
18	0	32.15	2
18	0	29.16	2
18	0	29.42	2
18	0	39	1
18	0	37.17	1
18	0	35.19	2
18	0	40.61	2
18	0	41.26	2
18	0	41.6	2
18	0	35	2
18	0	35	2
18	0	45.75	1
18	0	32.17	2
18	0	51.79	2
18	0	37.47	2
18	0	39.67	2
18	0	44.57	1
18	0	39	2
18	0	38.47	2
18	0	30.56	2
18	0	45.9	1
18	0	32.93	2
18	0	32	2
18	0	36	2
18	0	39.76	1
18	0	40.49	2
18	0	30.77	2
18	0	33	2
18	0	34	2

18	0	43.91	1
18	0	43.84	2
18	0	38.29	2
18	0	38.03	2
18	0	35.47	2
18	0	37.14	2
18	0	43.62	2
18	0	32.6	2
18	0	39	2
18	0	32.04	2
18	0	29.89	1
18	0	37	2
18	0	30.48	1
18	0	40.75	2
18	0	37	2
18	0	43	2
18	0	41.77	2
18	0	46.9	2
18	0	39.81	2
18	0	38.04	2
18	0	38.18	2
18	0	38	2
18	0	38	2
18	0	39	2
18	0	40.76	2
18	0	34.33	2
18	0	45.02	1
18	0	56.96	2
18	0	38.93	2
18	0	31.96	2
18	0	38.65	2
18	0	36.99	2
18	0	35	2
18	0	34.11	2
18	0	39.14	2
18	0	32.53	2
18	0	39.21	2
18	0	31.06	2
18	0	34.26	2
18	0	43.57	2
18	0	37	1
18	0	43.08	2
18	0	36.44	2
18	0	33.39	2
18	0	30.36	2
18	0	44.01	2
18	0	32.24	2

18	0	39	2
18	0	37.44	2
18	0	32.72	2
18	0	36	2
18	0	34	2
18	0	37.6	2
18	0	34	2
18	0	34	2
18	0	42.37	2
18	0	31.5	2
18	0	36.97	2
18	0	48.41	2
18	0	37	2
18	0	32	2
18	0	37.92	2
18	0	36.36	2
18	0	36.86	2
18	0	37.24	2
18	0	35.24	2
18	0	37.28	2
18	0	36	2
18	0	36.58	2
18	0	41.4	2
18	0	35.75	2
18	0	36	2
18	0	41.13	1
18	0	43.4	2
18	0	45.95	2
18	0	38	2
18	0	31.82	2
18	0	35.18	1
18	0	34.15	2
18	0	42.54	1
18	0	39.56	2
18	0	49.42	2
18	0	39	2
18	0	33.5	2
18	0	36.18	2
18	0	38.78	2
18	0	31.72	2
18	0	46.33	2
18	0	41.15	1
18	0	41.32	2
18	0	34.56	1
18	0	38.53	2
18	0	39.08	2
18	0	35.89	2

18	0	35.24	2
18	0	28.87	2
18	0	29.56	2
18	0	30.65	2
18	0	36	1
18	0	38.06	2
18	0	34	2
18	0	38.03	2
18	0	33.03	2
18	0	48.75	2
18	0	39	2
18	0	35	2
18	0	40	2
18	0	34	2
18	0	50.91	2
18	0	36.61	2
18	0	37.76	2
18	0	44.15	2
18	0	35	2
18	0	33	2
18	0	34.57	2
18	0	41.22	2
18	0	34	1
18	0	39.97	2
18	0	33.67	2
18	0	38.54	2
18	0	36	2
18	0	30.96	2
18	0	40	1
18	0	32.78	2
18	0	51.48	2
18	0	41.08	2
18	0	35.32	2
18	0	30.93	1
18	0	34.81	2
18	0	35.15	2
18	0	33.04	2
18	0	34.06	2
18	0	32.29	2
18	0	28.51	2
18	0	36.18	2
18	0	31.13	2
18	0	34	2
18	0	40	2
18	0	42.64	2
18	0	48.64	2
18	0	35.11	2

18	0	30.86	2
18	0	36	2
18	0	38.61	2
18	0	38.21	2
18	0	40.79	2
18	0	41.73	2
18	0	40.71	2
18	0	31.27	2
18	0	38	2
18	0	37.74	2
18	0	35	2
18	0	36.14	1
18	0	34.92	2
18	0	45.46	2
18	0	30.4	2
18	0	34.07	2
18	0	34	2
18	0	33.85	2
18	0	30.78	1
18	0	33	2
18	0	46.61	2
18	0	38.32	2
18	0	33	2
18	0	40	2
18	0	41.06	2
18	0	38.82	2
18	0	41.42	1
18	0	35.51	2
18	0	42.3	2
18	0	48.52	1
9	1	50.09	1
9	1	40.16	2
9	1	49.02	1
9	1	46.21	1
9	1	48.31	1
9	1	45.29	1
9	1	43.24	1
9	1	48	1
9	1	45.4	1
9	1	47.83	2
9	1	46.23	2
9	1	53.14	1
9	1	42.3	2
9	1	51.57	1
9	1	50.92	1
9	1	45.89	2
9	1	44.73	2

9	1	49.7	2
9	1	46.34	2
9	1	50.79	2
9	1	49.54	2
9	1	50.33	1
9	1	47.52	1
9	1	49.63	1
9	1	47.78	1
9	1	48.1	1
9	1	46.06	2
9	1	49.45	1
9	1	43.49	2
9	1	51.62	1
9	1	47.52	2
9	1	44.22	2
9	1	49.66	1
9	1	46.2	1
9	1	49.98	1
9	1	51.48	2
9	1	49	2
9	1	47.36	2
9	1	48.85	2
9	1	45.78	2
9	1	47.8	2
9	1	53.44	2
9	1	55.53	2
9	1	47.08	1
9	1	48.43	1
9	1	47.51	1
9	1	46.73	2
9	1	43.82	1
9	1	42.71	1
9	1	46.91	1
9	1	48.13	1
9	1	50.27	1
9	1	46.33	1
9	1	45.72	1
9	1	48.03	1
9	1	52.45	1
9	1	47.37	2
9	1	48.36	1
9	1	45.6	1
9	1	41.11	2
9	1	39.48	1
9	1	45.12	1
9	1	33.32	1
9	1	42.28	1

9	1	35.99	1
9	1	43.9	2
9	1	49.5	1
9	1	46.96	1
9	1	51.26	1
9	1	43.03	1
9	1	36.24	2
9	1	50.57	1
9	1	53.9	1
9	1	48.68	1
9	1	49.08	2
9	1	48.27	1
9	1	43.16	1
9	1	45.14	2
9	1	49.68	1
9	1	48	2
9	1	43.48	2
9	1	59.95	2
9	1	47.17	2
9	1	52.79	1
9	1	45.81	1
9	1	45.68	1
9	1	41.22	1
9	1	46.07	1
9	1	51.03	2
9	1	58.61	2
9	1	48.14	1
9	1	47.87	1
9	1	47.65	1
9	1	33.42	1
9	1	45.24	2
9	1	45.4	2
9	1	51.97	1
9	1	51.71	1
9	1	55.53	1
9	1	45.21	2
9	1	37.6	2
9	1	49.24	1
9	1	46.43	2
9	1	48.06	2
9	1	38.53	2
9	1	51.32	1
9	1	46.76	1
9	1	43.23	2
9	1	53.18	1
9	1	45.79	2
9	1	46.97	2

9	1	44.45	1
9	1	45.86	1
9	1	45.23	1
9	1	48.49	2
9	1	45.6	1
9	1	50.1	1
9	1	45.83	1
9	1	56.26	2
9	1	48.29	1
9	1	42.27	2
9	1	46.69	1
9	1	44.83	1
9	1	36.05	1
9	1	43.76	1
9	1	46.22	1
9	1	58.14	2
9	1	58.86	2
9	1	49.8	1
9	1	53.41	2
9	1	53.03	1
9	1	47.23	1
9	1	46.23	1
9	1	50.08	1
9	1	52.41	1
9	1	50	2
9	1	42.3	2
9	1	48.15	2
9	1	50.28	1
9	1	48.23	1
9	1	53.09	1
9	1	56.95	1
9	1	55.07	1
9	1	44.83	1
9	1	44.42	1
9	1	43.78	1
9	1	44.07	1
9	1	47.19	2
9	1	50	1
9	1	47.63	2
9	1	45.93	2
9	1	44.45	2
9	1	48	1
9	1	38.8	2
9	1	57.46	2
9	1	45.88	2
9	1	45	1
9	1	52.35	1

9	1	45.98	2
9	1	48.64	2
9	1	46.56	1
9	1	42.86	2
9	1	49.65	2
9	1	41.15	2
9	1	49.66	1
9	1	51.57	2
9	1	48.71	2
9	1	44.92	1
9	1	52.87	2
9	1	45.87	1
9	1	52.72	2
9	1	44.73	1
9	1	42	1
9	1	50.33	1
9	1	47.23	1
9	1	43.33	1
9	1	52.37	1
9	1	53.5	1
9	1	52	1
9	1	46	1
9	1	48.96	1
9	1	44	2
9	1	50.57	1
9	1	49.1	1
9	1	53.59	2
9	1	51	1
9	1	50.1	1
9	1	50	1
9	1	45.06	1
9	1	54.22	2
9	1	40.27	2
9	1	43.19	1
9	1	49.92	1
9	1	50.5	2
9	1	41.59	2
9	1	44.84	2
9	1	41.5	1
9	1	47	1
9	1	53.35	1
9	1	46.83	2
9	1	41.5	1
9	1	45.89	1
9	1	55.43	1
9	1	51.69	1
9	1	44.97	2

9	1	44.37	2
9	1	50.12	1
9	1	50.1	1
9	1	50.56	2
9	1	49.25	1
9	1	45.84	1
9	1	49.54	1
9	1	49.38	2
9	1	47.35	2
9	1	43	1
9	1	48.96	2
9	1	47.1	1
9	1	43.21	2
9	1	45	2
9	1	47.77	2
9	1	50.63	1
9	1	47.45	1
9	1	47.41	2
9	1	55.84	2
9	1	45.3	1
9	1	47.63	1
9	1	50.46	1
9	1	49.83	1
9	1	44	1
9	1	40.75	1
9	1	46.56	1
9	1	41.97	1
9	0	44.3	1
9	0	42.46	1
9	0	42.46	1
9	0	48.39	1
9	0	39.15	1
9	0	40.5	1
9	0	45.95	1
9	0	43.76	1
9	0	40.6	1
9	0	46	1
9	0	42.19	1
9	0	43.52	1
9	0	46.9	1
9	0	41.54	1
9	0	35.23	1
9	0	40.7	1
9	0	50.78	1
9	0	47.94	1
9	0	46.89	1
9	0	41.16	1

9	0	39.55	1
9	0	39.98	1
9	0	49.74	1
9	0	50.08	1
9	0	36.5	1
9	0	42.28	1
9	0	41.9	1
9	0	53.73	1
9	0	40.02	1
9	0	42.93	1
9	0	36.77	1
9	0	38.5	1
9	0	46.48	1
9	0	49.05	1
9	0	44.6	1
9	0	44.27	1
9	0	50.63	1
9	0	48.26	1
9	0	39.98	1
9	0	48.3	1
9	0	48.5	2
9	0	45.25	2
9	0	44.24	2
9	0	51.6	2
9	0	50.47	2
9	0	44.36	2
9	0	45.56	2
9	0	47.17	2
9	0	48.43	2
9	0	46.8	2
9	0	39.4	2
9	0	45.17	2
9	0	53.09	2
9	0	47.4	2
9	0	47.98	2
9	0	47.99	2
9	0	49.9	2
9	0	44.33	2
9	0	49.3	2
9	0	48.12	2
9	0	45.33	2
9	0	44.55	2
9	0	44.73	2
9	0	48.79	2
9	0	44.08	2
9	0	46.65	2
9	0	45.09	2

9	0	47.55	2
9	0	44.83	2
9	0	48.47	2
9	0	38.71	2
9	0	42.66	2
9	0	44.69	2
9	0	49.7	2
9	0	46.56	2
9	0	50.44	2
9	0	45.86	2
9	0	44.36	2
9	0	47.17	2
9	0	38.4	2
9	0	45.86	2
9	0	39.67	2
9	0	37.39	2
9	0	46.13	2
9	0	53.15	2
9	0	44.6	2
9	0	43.52	2
9	0	43.7	2
9	0	42.6	2
9	0	49.66	2
9	0	55.04	2
9	0	48.9	2
9	0	51.83	2
9	0	41.78	2
9	0	60.54	2
9	0	42.04	2
9	0	45.96	2
9	0	43.1	2
9	0	43	2
9	0	35.63	2
9	0	38.25	2
9	0	53	2
9	0	42.59	2
9	0	47.32	2
9	0	48.9	2
9	0	48.96	2
9	0	54.52	2
9	0	43.25	2
9	0	46.29	2
9	0	48.43	2
9	0	53.74	2
9	0	47.5	2
9	0	48.37	2
9	0	48.27	2

9	0	44.74	2
9	0	48.97	2
9	0	46.85	2
9	0	46.93	2
9	0	45.59	2
9	0	51.7	2
9	0	37.6	2
9	0	57.85	2
9	0	52.04	2
9	0	50.1	2
9	0	50.46	2
9	0	53.04	2
9	0	50.57	2
9	0	46.86	2
9	0	52.03	2
9	0	45.78	2
9	0	52.89	2
9	0	55.11	2
9	0	46.8	2
9	0	40.7	2
9	0	50.94	2
9	0	54.27	2
9	0	47.45	2
9	0	53.41	2
9	0	47.96	2
9	0	55.47	2
9	0	48.91	2
9	0	56.26	2
9	0	44.57	2
9	0	41.6	2
9	0	54.22	2
9	0	54.02	2
9	0	46.83	2
9	0	44.64	2
9	0	47.49	2
9	0	53.25	2
9	0	40.98	2
9	0	47.68	2
9	0	48.17	2
9	0	54.9	2
9	0	52.37	2
9	0	50.8	2
9	0	44.05	2
9	0	46.6	2
9	0	48.86	2
9	0	50.31	2
9	0	41.46	2

9	0	56.65	2
9	0	58.52	2
9	0	62.6	2
9	0	52	2
9	0	52.83	2
9	0	44.86	2
9	0	46.76	2
9	0	50.77	2
9	0	58.1	2
9	0	48.71	2
9	0	48.19	2
9	0	48.7	2
9	0	58.2	2
9	0	57.68	2
9	0	56.72	2
9	0	51.88	2
9	0	55.02	2
9	0	48.85	2
9	0	52.44	2
9	0	53.6	2
9	0	50.25	2
9	0	54.74	2
9	0	46.04	2
9	0	53	2
9	0	46.53	2
9	0	43.77	2
9	0	48.8	2
9	0	46	2
9	0	58.43	2
9	0	47.3	2
9	0	48.26	2
9	0	47.4	2
9	0	52.25	2
9	0	53.06	2
9	0	51.76	2
9	0	64.84	2
9	0	58.93	2
9	0	46.24	2
9	0	50.54	2
9	0	44.96	2
9	0	38.14	1
9	0	38.07	2
9	0	50.69	2
9	0	44.56	2
9	0	39.22	2
9	0	34.79	2
9	0	44.17	2

9	0	49.71	2
9	0	46.02	1
9	0	46.2	2
9	0	35.26	1
9	0	44.12	1
9	0	36.62	2
9	0	41.05	2
9	0	52.12	2
9	0	45.04	2
9	0	38.08	1
9	0	42.49	1
9	0	45.67	1
9	0	58.68	2
9	0	43.2	2
9	0	46.21	1
9	0	40.2	2
9	0	41.71	2
9	0	50.04	2
9	0	43.52	2
9	0	46.96	1
9	0	45.56	2
9	0	51.64	2
9	0	43.01	2
9	0	43.6	2
9	0	40.92	2
9	0	44.89	1
9	0	42.88	2
9	0	43.65	1
9	0	54.94	2
9	0	30.07	1
9	0	47.97	1
9	0	47.03	2
9	0	50.83	2
9	0	45.3	1
9	0	44.1	2
9	0	40.21	2
9	0	42.88	1
9	0	39.51	1
9	0	47.29	2
9	0	47.15	2
9	0	38.48	1
9	0	42.32	2
9	0	43.24	2
9	0	45.45	2
9	0	48.13	1
9	0	38.91	2
9	0	45.72	1

9	0	43.82	1
9	0	39.21	2
9	0	40.05	2
9	0	50.21	2
9	0	45.29	2
9	0	46.54	2
9	0	45.64	1
9	0	43.59	2
9	0	42.93	2
9	0	41.14	1
9	0	54.86	2
9	0	45.54	1
9	0	50.05	2
9	0	46.64	1
9	0	45.2	2
9	0	39.58	2
9	0	47.13	2
9	0	46.09	2
9	0	48.37	2
9	0	41.93	2
9	0	40.58	2
9	0	53.48	2
9	0	40.31	2
9	0	40	1
9	0	39.28	2
9	0	44.73	2
9	0	36.55	2
9	0	42.22	2
9	0	43.7	1
9	0	40	1
9	0	51.73	2
9	0	39.7	2
9	0	37.88	2
9	0	45.24	1
9	0	46.4	1
9	0	47.55	1
9	0	40.55	2
9	0	52.96	2
9	0	47.39	1
9	0	45.09	1
9	0	31.36	2
9	0	39.93	2
9	0	46.67	2
9	0	38.88	2
9	0	43.64	2

Scots Pine measurement in 2014

anvil	length	apophyses
2	46.64	0
2	46.84	0
2	41.21	0
2	48.46	0
2	44.34	0
2	43.48	0
2	32.47	0
2	44.94	0
2	38.84	0
2	47.16	0
2	33.77	0
2	45.42	0
2	45.67	0
2	37.18	0
2	46.22	0
2	44.53	0
2	45.1	0
2	41.72	0
2	46.85	0
2	47.4	0
2	38.49	0
2	41.48	0
2	42.95	0
2	43.9	0
2	39.57	0
2	46.22	0
2	45.19	0
2	45.12	0
1	43.27	0
1	39.27	0
1	40.09	0
1	41.35	0
1	40.04	0
1	44.39	0
1	44.79	0
1	36.32	0
1	41.05	0
1	46.93	0
1	36.3	0
1	34.54	0
1	30.92	0
1	38.28	0
1	41.47	0
1	40.99	0

1	43.74	0
1	30.58	0
1	42.95	0
1	38.32	0
1	43.68	0
1	36.83	0
1	38.64	0
1	45.84	0
1	41.59	0
11	31.42	0
11	42.05	0
11	32.23	0
11	35.97	0
11	37.19	0
11	44.61	0
11	37.28	0
11	45	0
11	37.15	0
11	35.74	0
11	41.01	0
11	35.11	0
11	32.38	0
11	35.68	0
11	33.46	0
11	42.02	0
11	39.67	0
11	41.02	0
11	32.94	0
11	39.68	0
11	40.62	0
11	35.11	0
11	40.92	0
11	41.5	0
11	37.91	0
11	34.91	0
11	42.95	0
11	46.42	0
11	35.05	0
11	39.86	0
11	32.35	0
11	38.65	0
11	41.43	0
11	32.3	0
11	35.11	0
11	38.39	0
11	42.51	0
11	35.88	0

11	35.67	0
3	46.5	0
3	50.96	0
3	40.93	0
3	47.3	0
3	29.27	0
3	32.86	0
3	46.15	0
3	42.15	0
3	44.68	0
3	44.68	0
3	39.86	0
3	45.53	0
3	49.43	0
3	37.98	0
3	43.6	0
3	48.13	0
3	39.7	0
3	47.55	0
3	37.44	0
3	44.61	0
3	41.09	0
3	46.9	0
3	40.89	0
3	38.96	0
3	42.59	0
3	43	0
8	38.86	0
8	50.8	0
8	48.57	0
8	47.85	0
8	53.8	0
8	42.15	0
8	44.45	0
8	40.11	0
8	42.33	0
8	32.98	0
8	48.23	0
8	45.81	0
8	46.89	0
8	47.65	0
8	45.2	0
8	46	0
8	45.32	0
8	34.14	0
8	45.95	0
8	43.2	0

8	41.2	0
17	44.3	0
17	42.46	0
17	42.46	0
17	48.39	0
17	39.15	0
17	40.5	0
17	45.95	0
17	43.76	0
17	40.6	0
17	46	0
17	42.19	0
17	43.52	0
17	46.9	0
17	41.54	0
17	35.23	0
17	40.7	0
17	50.78	0
17	47.94	0
17	46.89	0
17	41.16	0
17	39.55	0
17	39.98	0
17	49.74	0
17	50.08	0
17	36.5	0
17	42.28	0
17	41.9	0
17	53.73	0
17	40.02	0
17	42.93	0
17	36.77	0
17	38.5	0
17	46.48	0
17	49.05	0
17	44.6	0
17	44.27	0
17	50.63	0
17	48.26	0
17	39.98	0
17	48.3	0
18	39.81	0
18	38.22	0
18	40.32	0
18	38.5	0
18	42.05	0
18	52.76	0

18	51.26	0
18	43.45	0
18	40.76	0
18	34.98	0
18	40.06	0
18	45.36	0
18	46.49	0
18	41.59	0
18	37.59	0
18	40.32	0
18	48.86	0
18	40.76	0
18	34.82	0
18	40.08	0
18	41.23	0
18	36.74	0
18	38.42	0
18	36.87	0
18	43.13	0
18	45.71	0
18	34.19	0
18	41.96	0
18	51.44	0
18	39.02	0
18	47.15	0
18	42.68	0
18	49	0
18	35.48	0
18	46.47	0
18	40.68	0
18	43.95	0
18	45.65	0
18	52.33	0
18	46.56	0
18	37.82	0
18	34.12	0
10	37.78	0
10	43.04	0
10	45.84	0
10	38.39	0
10	42.17	0
10	43.54	0
10	35.82	0
10	37.5	0
10	47.07	0
10	42.38	0
10	50.58	0

10	44.09	0
10	45.2	0
10	46.46	0
10	43.42	0
10	41.63	0
10	43.29	0
10	39.61	0
10	45.43	0
10	45.81	0
10	48.05	0
10	41.25	0
10	42.14	0
10	36.8	0
10	33.77	0
10	39.95	0
10	37.49	0
10	39.03	0
10	47.02	0
10	36.51	0
10	44.19	0
10	41.56	0
10	41.69	0
10	45.94	0
10	43.14	0
10	39.97	0
10	38.63	0
10	37.39	0
10	43.41	0
10	42.28	0
10	36.15	0
10	35.17	0
10	46.94	0
10	41.17	0
10	46.21	0
10	45.39	0
10	47.43	0
10	35.33	0
10	34.44	0
10	40.81	0
10	45.96	0
10	33.77	0
10	44.03	0
10	40.63	0
10	40.2	0
10	37.02	0
10	35.33	0
10	37.25	0

10	46.59	0
10	36.72	0
10	38.06	0
10	41.46	0
10	39.75	0
10	42.87	0
10	40.08	0
10	40.54	0
10	58.07	0
10	36.24	0
10	37.98	0
10	39.09	0
10	33.09	0
10	48.18	0
10	44.78	0
10	32.75	0
2	42.37	1
2	38.64	1
2	41.24	1
2	41.05	1
2	40.36	1
2	42.78	1
2	42.59	1
2	41.65	1
2	45.08	1
2	43.66	1
2	41.92	1
2	43.49	1
2	45.45	1
2	38.65	1
2	43.4	1
2	32.2	1
2	43.42	1
2	42.69	1
2	42.38	1
2	44.52	1
2	43.83	1
2	42.76	1
2	37.41	1
2	39.44	1
2	38.8	1
2	41.49	1
2	44.86	1
2	38.21	1
2	39.82	1
2	47.84	1
2	43.9	1

2	41.64	1
2	42.93	1
2	36.6	1
2	49.27	1
2	41.28	1
2	42.82	1
2	43.3	1
2	40.77	1
2	39.62	1
2	43.51	1
2	46.54	1
2	40.51	1
2	42.79	1
2	39.94	1
2	36.32	1
2	39.82	1
2	43.38	1
2	42.31	1
2	42.62	1
2	43.16	1
2	41.37	1
2	47.98	1
2	46.85	1
2	38.2	1
2	34.63	1
2	39.26	1
2	47.76	1
2	44.97	1
2	38.6	1
2	39.42	1
2	46.19	1
1	43.89	1
1	42.89	1
1	34.08	1
1	38.92	1
1	47.36	1
1	49.52	1
1	41.67	1
1	45.35	1
1	43.32	1
1	43.12	1
1	42.17	1
1	34.23	1
1	49.05	1
1	39.17	1
1	33.68	1
1	42.34	1

1	42.6	1
1	35.44	1
1	43.85	1
1	47.03	1
1	33.7	1
1	43.86	1
1	37.77	1
1	46.16	1
1	42.42	1
1	38.36	1
1	36.22	1
1	42.01	1
1	36.89	1
1	36.75	1
1	41.38	1
1	41.98	1
1	42.46	1
1	48.61	1
1	42.58	1
1	41.23	1
1	43.39	1
1	34.53	1
1	42.97	1
1	44.52	1
1	40.92	1
1	38.53	1
1	43.66	1
1	36.09	1
1	39.01	1
1	40.94	1
1	44.62	1
1	43.94	1
1	36.98	1
1	45.76	1
1	43.45	1
1	35.12	1
1	46.37	1
1	38.13	1
1	39.2	1
1	37.11	1
1	43.21	1
1	44.39	1
1	37.54	1
1	37.42	1
1	38.56	1
1	37.11	1
1	39.78	1

1	47.58	1
1	41.97	1
1	45.49	1
1	46.48	1
1	47.49	1
1	49.58	1
1	43.93	1
1	40.18	1
1	34.05	1
1	46.97	1
1	42.51	1
1	39.02	1
1	46.79	1
1	41.78	1
1	47.68	1
1	44.37	1
1	46.29	1
1	41.01	1
1	50.53	1
1	43.03	1
1	40.27	1
11	45.35	1
11	45.31	1
11	47.61	1
11	35.6	1
11	50.47	1
11	41.23	1
11	37.22	1
11	41.08	1
11	39.15	1
11	35.9	1
11	30.74	1
11	40.51	1
11	30.28	1
11	50.79	1
11	44.36	1
11	48.04	1
11	44.49	1
11	39.35	1
11	35.93	1
11	40.56	1
11	39.85	1
11	39.88	1
11	33.28	1
11	30.95	1
11	47.76	1
11	43.1	1

11	47.56	1
11	50.43	1
11	44.77	1
11	38.94	1
11	39.42	1
11	48.82	1
11	42.93	1
11	33.55	1
11	35.11	1
11	33.32	1
11	34.68	1
11	47.33	1
11	31.84	1
11	27.53	1
11	48.03	1
11	33.44	1
11	38	1
11	36.29	1
11	36.59	1
11	50.38	1
11	46.08	1
11	30.07	1
11	36.09	1
11	45.62	1
11	39.63	1
11	42.16	1
11	35.8	1
11	38.48	1
11	38.61	1
11	37.28	1
11	31.41	1
11	33.54	1
11	37.46	1
11	41.61	1
11	42.6	1
11	36.55	1
11	31.8	1
11	44.96	1
11	36.4	1
11	43.64	1
11	39.51	1
3	35.61	1
3	42.46	1
3	41.33	1
3	40.29	1
3	38.64	1
3	43.35	1

3	39.5	1
3	41.83	1
3	42.26	1
3	40.91	1
3	37.5	1
3	39.04	1
3	44.64	1
3	38.7	1
3	36.41	1
3	41.56	1
3	42.4	1
3	43.32	1
3	45.57	1
3	40.63	1
3	42.86	1
3	38.15	1
3	43.35	1
3	49.5	1
3	35.75	1
3	39.58	1
3	42.12	1
3	36.98	1
3	43.25	1
3	40.49	1
3	38.96	1
3	40.57	1
3	39.3	1
3	44.39	1
3	43.63	1
3	40.85	1
3	46.16	1
3	40.93	1
3	37.77	1
3	48.1	1
3	47.1	1
3	45.54	1
3	43.57	1
3	41	1
3	40.66	1
3	48	1
3	38.89	1
3	45.18	1
3	42.33	1
3	51.51	1
3	40.3	1
3	38.26	1
3	40.3	1

3	37.14	1
3	42.8	1
3	48.43	1
3	47.15	1
8	46.15	1
8	39.03	1
8	42.66	1
8	52.37	1
8	39.23	1
8	41.5	1
8	38.14	1
8	42.65	1
8	36.79	1
8	37.26	1
8	39.24	1
8	34.8	1
8	40.15	1
8	47.61	1
8	45.11	1
8	36.69	1
8	42.09	1
8	40.71	1
8	42.72	1
8	37.04	1
8	42.88	1
8	39.02	1
8	45.61	1
8	32.31	1
8	38.96	1
8	32.58	1
8	34.56	1
8	44.66	1
8	38.9	1
8	36.28	1
17	48.5	1
17	45.25	1
17	44.24	1
17	51.6	1
17	50.47	1
17	44.36	1
17	45.56	1
17	47.17	1
17	48.43	1
17	46.8	1
17	39.4	1
17	45.17	1
17	53.09	1

17	47.4	1
17	47.98	1
17	47.99	1
17	49.9	1
17	44.33	1
17	49.3	1
17	48.12	1
17	45.33	1
17	44.55	1
17	44.73	1
17	48.79	1
17	44.08	1
17	46.65	1
17	45.09	1
17	47.55	1
17	44.83	1
17	48.47	1
17	38.71	1
17	42.66	1
17	44.69	1
17	49.7	1
17	46.56	1
17	50.44	1
17	45.86	1
17	44.36	1
17	47.17	1
17	38.4	1
17	45.86	1
17	39.67	1
17	37.39	1
17	46.13	1
17	53.15	1
17	44.6	1
17	43.52	1
17	43.7	1
17	42.6	1
17	49.66	1
17	55.04	1
17	48.9	1
17	51.83	1
17	41.78	1
17	60.54	1
17	42.04	1
17	45.96	1
17	43.1	1
17	43	1
17	35.63	1

17	38.25	1
17	53	1
17	42.59	1
17	47.32	1
17	48.9	1
17	48.96	1
17	54.52	1
17	43.25	1
17	46.29	1
17	48.43	1
17	53.74	1
17	47.5	1
17	48.37	1
17	48.27	1
17	44.74	1
17	48.97	1
17	46.85	1
17	46.93	1
17	45.59	1
18	43.79	1
18	44.11	1
18	53.89	1
18	49.05	1
18	48.92	1
18	41	1
18	45.84	1
18	38.08	1
18	48.58	1
18	49.26	1
18	42.16	1
18	40.42	1
18	51	1
18	46.84	1
18	43.38	1
18	40.08	1
18	42.24	1
18	42.06	1
18	43.05	1
18	47.69	1
18	43.84	1
18	45.78	1
18	47.11	1
18	45.48	1
18	49.33	1
18	46.42	1
18	36.36	1
18	35.31	1

18	47.64	1
18	52.06	1
18	40.6	1
18	48.29	1
18	49.73	1
18	41.31	1
18	49.68	1
18	46.58	1
18	47.43	1
18	49.05	1
18	50.15	1
18	38.16	1
18	38.24	1
18	34.68	1
18	40.69	1
18	39.69	1
18	34.41	1
18	43.56	1
18	48.46	1
18	51.65	1
18	52.21	1
18	42.82	1
18	44.07	1
18	50.42	1
18	44.96	1
18	45.8	1
18	42.75	1
18	41.9	1
18	36.86	1
18	38.37	1
18	48.5	1
18	42.76	1
18	44.87	1
18	40.86	1
18	42.39	1
18	38.6	1
18	46.35	1
18	39.76	1
18	46.29	1
18	43.69	1
18	41.02	1
18	46.62	1
18	40.75	1
18	42.43	1
18	37.08	1
18	39.55	1
18	48.72	1

18	46.27	1
18	44.76	1
18	34.4	1
18	44.14	1
18	45.99	1
18	56.21	1
18	45.36	1
18	45.03	1
18	48.1	1
18	48.3	1
18	29.89	1
18	44.47	1
18	34.25	1
18	49.37	1
18	46.64	1
18	35.94	1
18	43.57	1
18	38.47	1
18	37.69	1
18	51.68	1
18	56.05	1
18	39.29	1
18	42.09	1
18	40.41	1
18	40.07	1
18	40.18	1
18	41.74	1
18	43.51	1
18	51.64	1
18	41.17	1
18	40.45	1
18	33.43	1
18	45.02	1
18	40.57	1
18	43.83	1
18	46.82	1
18	49.3	1
18	35.76	1
18	41.21	1
18	41.65	1
18	37.11	1
18	46.7	1
18	51.34	1
18	37.44	1
18	46.77	1
18	44.98	1
18	37.65	1

18	36.79	1
18	44.3	1
18	47.01	1
18	45.84	1
18	40.09	1
18	35.71	1
18	36.91	1
18	38.96	1
10	48.56	1
10	38.1	1
10	37.55	1
10	44.8	1
10	35.25	1
10	42.07	1
10	35.65	1
10	53.2	1
10	33.82	1
10	44.51	1
10	40.35	1
10	41.97	1
10	41.7	1
10	36.17	1
10	42.17	1
10	40.66	1
10	38.08	1
10	40.7	1
10	28.72	1
10	39.88	1
10	45.15	1
10	46.69	1
10	47.98	1
10	41.73	1
10	41.46	1
10	49.89	1
10	40.53	1
10	43.19	1
10	40.14	1
10	49.78	1
10	39.06	1
10	45.07	1
10	34.92	1
10	40.31	1
10	49.26	1
10	36.02	1
10	37.49	1
10	40.62	1
10	44.07	1

10	33.53	1
10	42.57	1
10	37.04	1
10	42.29	1
10	40.09	1
10	46.04	1
10	36.63	1
10	39.79	1
10	46.1	1
10	34.89	1
10	38.86	1
10	42.83	1
10	50.94	1
10	49.21	1
10	47.11	1
10	39.26	1
10	41.93	1
10	44.99	1
2	45.06	1
2	43.36	1
2	49.58	1
2	45.36	1
2	44.63	1
2	38.26	1
2	45.47	1
2	36.04	1
2	39.43	1
2	41.76	1
2	41.33	1
2	44.96	1
2	42.66	1
2	42.15	1
2	45.74	1
2	45.4	1
2	39.95	1
2	44.89	1
2	44.02	1
2	46.01	1
2	46.02	1
2	44.24	1
2	43.21	1
2	52.27	1
2	40.49	1
2	40.83	1
2	34.77	1
2	42.05	1
2	44.83	1

2	46.29	1
2	41.96	1
2	39.19	1
2	44.1	1
2	37.28	1
2	33.32	1
2	43.66	1
2	46.42	1
2	36.45	1
2	40.83	1
2	46.91	1
2	38.84	1
2	41.04	1
2	42.05	1
2	44	1
2	46.17	1
2	40.77	1
2	47.25	1
2	46.36	1
2	39.81	1
2	39.02	1
2	41.65	1
2	46.8	1
2	41.94	1
2	47.62	1
2	41.42	1
2	53.26	1
2	45.52	1
2	38.88	1
2	44.53	1
2	42.9	1
2	46.78	1
2	41.49	1
2	44.59	1
2	45.75	1
2	47.32	1
2	40.22	1
2	42.36	1
2	45.98	1
2	49.1	1
2	46.68	1
2	41.09	1
2	40.66	1
2	45.87	1
2	47.02	1
2	41.07	1
2	45.96	1

2	37.25	1
2	39.84	1
2	45.07	1
2	38.64	1
2	46.84	1
2	36.15	1
2	47.19	1
2	40.94	1
2	51.1	1
2	43.64	1
2	48	1
2	44.49	1
2	41.86	1
2	42.21	1
2	46.04	1
2	50.62	1
2	45.05	1
2	49.13	1
2	43.18	1
2	39.13	1
2	42.17	1
2	39.17	1
2	44.65	1
2	43.19	1
2	44.55	1
2	43.88	1
2	43.86	1
2	45.4	1
2	37.72	1
2	46.63	1
2	39.03	1
2	40.88	1
2	47	1
2	37.23	1
1	42.93	1
1	42.09	1
1	39.8	1
1	41.84	1
1	41.93	1
1	42.89	1
1	38.27	1
1	45.45	1
1	41.19	1
1	42.24	1
1	43.89	1
1	45.08	1
1	42.87	1

1	44.63	1
1	45.31	1
1	42.91	1
1	37.69	1
1	40.43	1
1	35.91	1
1	32.36	1
1	39.22	1
1	41.37	1
1	43.69	1
1	47.8	1
1	39.06	1
1	43.2	1
1	42.49	1
1	44.42	1
1	48.03	1
1	44.63	1
1	38.53	1
1	41.52	1
1	42.51	1
1	42.48	1
1	44.91	1
1	44.47	1
1	35.84	1
1	48.72	1
1	44.06	1
1	50.94	1
1	40.46	1
1	41.52	1
1	41.49	1
1	43.6	1
1	44.39	1
1	45.69	1
1	41.67	1
1	44.12	1
1	40.05	1
1	46.95	1
1	44.61	1
1	40.9	1
1	42.48	1
1	40.96	1
1	39.1	1
1	40.3	1
1	43.99	1
1	43.05	1
1	43.66	1
1	39.42	1

1	38.76	1
1	45.86	1
1	41.16	1
1	44.88	1
1	47.93	1
1	47.14	1
1	48.1	1
1	46.04	1
1	36.95	1
1	44.47	1
1	44.81	1
1	36.72	1
1	46.04	1
1	44.48	1
1	41.42	1
1	38.27	1
1	45.84	1
1	42.16	1
1	50.86	1
1	44.04	1
1	47.74	1
1	49.07	1
1	50.14	1
1	46.42	1
1	38.53	1
1	41.86	1
1	43.29	1
1	44.18	1
1	49.12	1
1	39	1
1	48.51	1
11	50.79	1
11	43.06	1
11	48.1	1
11	42.22	1
11	47.66	1
11	48.33	1
11	49.3	1
11	47.46	1
11	49.43	1
11	48.06	1
11	51.05	1
11	32.88	1
11	48.92	1
11	42	1
11	46.45	1
11	33.38	1

11	44.61	1
11	49.4	1
11	29.39	1
11	41.17	1
11	43.98	1
11	50.55	1
11	34.52	1
11	39.47	1
11	37.81	1
11	47.34	1
11	36.9	1
11	48.49	1
11	43.81	1
11	32.37	1
11	52.43	1
11	48	1
11	42.86	1
11	34.5	1
11	37.23	1
11	44.5	1
11	52.29	1
11	42.62	1
11	46.54	1
11	39.83	1
11	35.76	1
11	44.02	1
11	34.4	1
11	45.61	1
11	47.97	1
11	44.76	1
11	44.55	1
11	35.68	1
11	41.94	1
11	40.16	1
11	36.57	1
11	47.5	1
11	38.13	1
11	31.68	1
11	45.46	1
11	40.11	1
11	45.65	1
11	42.89	1
11	42.09	1
11	45.34	1
11	53.63	1
11	47.36	1
11	40.72	1

11	51.62	1
11	49.33	1
11	42.49	1
11	32.7	1
11	53.22	1
11	55.04	1
11	43.98	1
11	47.14	1
11	40.91	1
11	50.41	1
11	49.07	1
11	35.98	1
11	33.03	1
11	50.42	1
11	51.79	1
11	29.37	1
11	39.69	1
11	42.24	1
11	32.13	1
11	46.02	1
11	34.24	1
11	49.5	1
11	47.55	1
11	37.24	1
11	50.14	1
11	49.34	1
11	48.04	1
11	51.82	1
11	42.18	1
11	43.92	1
11	34.44	1
3	53.29	1
3	49.24	1
3	52.57	1
3	45.6	1
3	52.98	1
3	53	1
3	45	1
3	39.8	1
3	40.65	1
3	47.7	1
3	48.74	1
3	37.42	1
3	43.15	1
3	39.36	1
3	51.56	1
3	48.65	1

3	42.99	1
3	35.36	1
3	46.48	1
3	43.76	1
3	31.25	1
3	41.2	1
3	41.4	1
3	51.26	1
3	55.72	1
3	47.3	1
3	43.5	1
3	39.11	1
3	45.9	1
3	52.27	1
3	45.96	1
3	43.45	1
3	42.42	1
3	44.03	1
3	49.66	1
3	51.17	1
3	43.91	1
3	40.29	1
3	55.02	1
3	47.08	1
3	45.65	1
3	38.79	1
3	40.88	1
3	52.77	1
3	46.55	1
3	49	1
3	48.15	1
3	47.38	1
3	46.58	1
3	43.8	1
3	42.96	1
3	42	1
3	43.28	1
3	33.74	1
3	59.43	1
3	46.76	1
3	52.93	1
3	42.75	1
3	40.95	1
3	46.46	1
3	49.7	1
3	47.56	1
3	51.37	1

3	37.6	1
3	43.59	1
3	42.77	1
3	43.21	1
3	54.56	1
3	46.09	1
3	41.82	1
3	44.51	1
3	41	1
3	33.58	1
3	38.97	1
3	44.96	1
3	48.15	1
3	45.7	1
3	46.09	1
3	48.16	1
3	44.4	1
3	45.08	1
3	44.97	1
3	46.37	1
3	34.76	1
3	48.77	1
3	49.74	1
3	45.77	1
3	39.16	1
3	46.97	1
3	40.1	1
3	47.83	1
3	35.19	1
3	43.81	1
3	41.52	1
3	43.35	1
3	48.26	1
3	47.27	1
3	49.72	1
3	47	1
3	49.2	1
3	39	1
3	42.94	1
3	41.35	1
3	46.52	1
3	48.9	1
3	44	1
3	46.51	1
3	40.6	1
3	46.35	1
3	43.53	1

3	42.48	1
3	46.32	1
3	41.78	1
3	50.21	1
3	45.37	1
3	41.96	1
3	41.68	1
8	40.64	1
8	45.44	1
8	56.65	1
8	43.66	1
8	41.24	1
8	41.94	1
8	47.91	1
8	38.27	1
8	45.2	1
8	43.47	1
8	47.05	1
8	46.03	1
8	38.66	1
8	40.8	1
8	45.3	1
8	49.96	1
8	43.77	1
8	44.23	1
8	42.74	1
8	39.14	1
8	44.1	1
8	44.38	1
8	48.21	1
8	46.93	1
8	34.9	1
8	45.34	1
8	39.32	1
8	53.1	1
8	42.78	1
8	48.39	1
8	42.69	1
8	44.83	1
8	46.83	1
8	48	1
8	40.84	1
8	40.93	1
8	45.5	1
8	47.91	1
8	41.5	1
8	48.06	1

8	50.23	1
8	43.07	1
8	47.09	1
8	41.25	1
8	38.49	1
8	47.14	1
8	49.13	1
8	41.4	1
8	43.17	1
8	44.69	1
8	46.09	1
8	44.65	1
8	48.9	1
8	45.62	1
8	45.5	1
8	39.8	1
8	45.79	1
8	51.44	1
8	42.19	1
8	43.6	1
8	42.58	1
8	42.07	1
8	51.86	1
8	47.33	1
8	49.28	1
8	42.65	1
8	43.28	1
8	43.92	1
8	38.85	1
8	40.75	1
8	50.21	1
8	46.22	1
8	47.96	1
8	46.17	1
8	52.81	1
8	39.57	1
8	50.08	1
8	40.47	1
8	45.63	1
8	43.02	1
8	53.65	1
8	40.64	1
8	43.1	1
8	53.83	1
8	51.5	1
8	48.51	1
8	40.98	1

8	51.24	1
8	41.83	1
8	50.93	1
8	40.6	1
8	50.87	1
8	51.4	1
8	47.97	1
8	40.33	1
8	43.5	1
8	47	1
8	53.6	1
8	47.74	1
8	44.5	1
8	40.66	1
8	53.01	1
8	64.69	1
8	44.16	1
8	51.74	1
8	49.2	1
8	47.83	1
8	35.92	1
8	57.96	1
8	55.06	1
8	52	1
8	40.08	1
8	42.29	1
8	49.94	1
8	43.46	1
8	42.63	1
8	52.33	1
8	45.94	1
8	54.7	1
8	46.33	1
8	34.15	1
8	41.16	1
8	48.37	1
8	38.31	1
8	46.9	1
8	48.25	1
8	45.25	1
8	52.93	1
8	45.9	1
8	45.6	1
8	48.98	1
8	50.61	1
8	43.18	1
8	37.12	1

8	54.79	1
8	44.66	1
8	48.43	1
8	50.63	1
8	33.27	1
8	49.55	1
8	49.09	1
8	44.69	1
8	48	1
8	56.52	1
8	46.4	1
8	43.62	1
8	46.3	1
8	40.9	1
8	51.5	1
17	51.7	1
17	37.6	1
17	57.85	1
17	52.04	1
17	50.1	1
17	50.46	1
17	53.04	1
17	50.57	1
17	46.86	1
17	52.03	1
17	45.78	1
17	52.89	1
17	55.11	1
17	46.8	1
17	40.7	1
17	50.94	1
17	54.27	1
17	47.45	1
17	53.41	1
17	47.96	1
17	55.47	1
17	48.91	1
17	56.26	1
17	44.57	1
17	41.6	1
17	54.22	1
17	54.02	1
17	46.83	1
17	44.64	1
17	47.49	1
17	53.25	1
17	40.98	1

17	47.68	1
17	48.17	1
17	54.9	1
17	52.37	1
17	50.8	1
17	44.05	1
17	46.6	1
17	48.86	1
17	50.31	1
17	41.46	1
17	56.65	1
17	58.52	1
17	62.6	1
17	52	1
17	52.83	1
17	44.86	1
17	46.76	1
17	50.77	1
17	58.1	1
17	48.71	1
17	48.19	1
17	48.7	1
17	58.2	1
17	57.68	1
17	56.72	1
17	51.88	1
17	55.02	1
17	48.85	1
17	52.44	1
17	53.6	1
17	50.25	1
17	54.74	1
17	46.04	1
17	53	1
17	46.53	1
17	43.77	1
17	48.8	1
17	46	1
17	58.43	1
17	47.3	1
17	48.26	1
17	47.4	1
17	52.25	1
17	53.06	1
17	51.76	1
17	64.84	1
17	58.93	1

17	46.24	1
17	50.54	1
18	46.77	1
18	43.73	1
18	40.16	1
18	42.63	1
18	44.97	1
18	42.18	1
18	44.79	1
18	49.18	1
18	40.38	1
18	43.86	1
18	36.61	1
18	42.07	1
18	42.91	1
18	42.48	1
18	42.66	1
18	42.9	1
18	44.84	1
18	44.67	1
18	50.91	1
18	42.49	1
18	34.28	1
18	41.54	1
18	37.6	1
18	41	1
18	45.8	1
18	44.52	1
18	42.83	1
18	37.16	1
10	37.96	1
10	41.3	1
10	35.68	1
10	38.07	1
10	42.47	1
10	46.3	1
10	41.41	1
10	32.13	1
10	44.45	1
10	46.54	1
10	43.26	1
10	43.58	1
10	37.91	1
10	47.23	1
10	45.27	1
10	39.54	1
10	38.44	1

10	46.14	1
10	43.57	1
10	45.27	1
10	42.5	1
10	44.6	1
10	43.71	1
10	46.12	1
10	41.99	1
10	43.42	1
10	37.49	1
10	42.96	1
10	49.84	1
10	43.59	1
10	39.96	1
10	42.96	1
10	46.8	1
10	44.55	1
10	41.45	1
10	42.94	1
10	48.68	1
10	49.1	1
10	48.85	1
10	47.86	1
10	46.44	1
10	42.31	1
10	42.77	1
10	38.57	1
10	42.19	1
10	42.12	1
10	41.33	1
10	43.47	1
10	53.03	1
10	45.65	1
10	43.06	1
10	40.93	1
10	44.19	1
10	45.4	1
10	47.33	1
10	55.34	1
10	53.18	1
10	38.09	1
10	46.94	1
10	40.84	1
10	51.18	1
10	39.8	1
10	62.8	1
10	38.47	1

10	40.13	1
10	59.92	1
10	55.68	1
10	43.94	1
10	43.61	1
